# The *Streptococcus pyogenes* fibronectin/tenascin-binding protein PrtF.2 contributes to virulence in an influenza superinfection

**DOI:** 10.1038/s41598-018-29714-x

**Published:** 2018-08-14

**Authors:** Andrea L. Herrera, Haddy Faal, Danielle Moss, Leslie Addengast, Lauren Fanta, Kathleen Eyster, Victor C. Huber, Michael S. Chaussee

**Affiliations:** Division of Basic Biomedical Sciences, The Sanford School of Medicine of the University of South Dakota, Vermillion, South Dakota USA

## Abstract

Influenza A virus (IAV) and *Streptococcus pyogenes* (the group A Streptococcus; GAS) are important contributors to viral-bacterial superinfections, which result from incompletely defined mechanisms. We identified changes in gene expression following IAV infection of A549 cells. Changes included an increase in transcripts encoding proteins with fibronectin-type III (FnIII) domains, such as fibronectin (Fn), tenascin N (TNN), and tenascin C (TNC). We tested the idea that increased expression of TNC may affect the outcome of an IAV-GAS superinfection. To do so, we created a GAS strain that lacked the Fn-binding protein PrtF.2. We found that the wild-type GAS strain, but not the mutant, co-localized with TNC and bound to purified TNC. In addition, adherence of the wild-type strain to IAV-infected A549 cells was greater compared to the *prtF.2* mutant. The wild-type strain was also more abundant in the lungs of mice 24 hours after superinfection compared to the mutant strain. Finally, all mice infected with IAV and the *prtF.2* mutant strain survived superinfection compared to only 42% infected with IAV and the parental GAS strain, indicating that PrtF.2 contributes to virulence in a murine model of IAV-GAS superinfection.

## Introduction

Influenza A virus (IAV) is a highly contagious seasonal virus, which causes significant morbidity and mortality. While most IAV infections result in mild to moderate respiratory disease, secondary bacterial infections, or superinfections, substantially exacerbate the illness and increase mortality^1^. Superinfections associated with IAV are primarily caused by *Streptococcus pneumoniae*, *Staphylococcus aureus*, *Haemophilus influenzae*, and *Streptococcus pyogenes* (the Group A Streptococcus; GAS)^2^. Periodic IAV pandemics can affect up to 20% of the population and during past pandemics superinfections greatly increased IAV-associated mortality^3,4^. An analysis of lung biopsies from the 1918 influenza pandemic showed that *S. pneumoniae* and GAS were the most frequently observed bacteria in the lung and together contributed to approximately 90% of all IAV related deaths^5^. During the 2009 H1N1 IAV pandemic, *S. pneumoniae* and *S. aureus* were the most frequent causes of bacterial superinfection^6–9^; however, 27% of all deaths due to IAV-bacterial superinfections during this pandemic were associated with GAS, even though GAS is a relatively uncommon cause of pneumonia. Thus IAV-GAS superinfections are relatively uncommon, however the mortality is significant^6–10^. Studies done during the 1957 pandemic showed that bacteria were attached to lung tissues in areas where the epithelial cell layer had been destroyed by prior viral infection^5,11^. These and several additional studies (summarized by McCullers)^2^ indicate that IAV infection enhances bacterial adherence, which increases the severity of disease.

GAS binding to fibronectin (Fn) is among the many ways GAS can adhere to host cells and tissues. IAV infections increase the abundance and accessibility of Fn in several ways. First, IAV infections are thrombotic resulting in increased production in the lungs of Fn and fibrinogen (Fg)^12–15^, both of which are well characterized GAS ligands. Second, IAV neuraminidase removes sialic acid from the surfaces of host cells thereby increasing the accessibility of potential receptors for adherence, including Fn^16,17^. Third, IAV neuraminidase activates TGF-β through the cleavage of sialic acids on the secreted TGF-β protein^18^. This results in a signaling cascade, which induces Fn expression and enhances bacterial adherence (including the adherence of GAS)^19–25^. Moreover, treating IAV-infected A549 cells with inhibitors of TGF-β signaling decreased Fn expression and the adherence of GAS^25^.

A variety of GAS proteins bind to Fn and many isolates encode multiple Fn-binding proteins^26–33^. Mutants created in GAS that lack specific Fn-binding surface proteins are less virulent in animal models of infection, which is consistent with their role in colonization and pathogenesis^27,32,33^. At least 11 Fn-binding proteins have been identified in various clinical isolates of GAS including Protein F2 (PrtF.2)^28,29,31–38^. The *prtF.2* gene is present in the chromosomes of 36–80% of clinical isolates^39–41^ and the protein contributes to GAS attachment to, and entry into, epithelial cells^35,41^. PrtF.2 has 47% amino acid sequence identity with a similar protein named Protein F1 (encoded by *prtF.1*). Clinical isolates that have the *prtF.2* gene, such as strain MGAS315, typically lack *prtF.1*^42^. The carboxyl termini of both proteins have two separate high affinity Fn-binding domains^35,42^.

Fn is a dimeric protein composed of two identical monomers. Among each monomer there are three different types of repeating domains. The repeating domains include 12 fibronectin-type I (FnI) domains, 2 fibronectin-type II (FnII) domains, and 15–17 fibronectin-type III (FnIII) domains^43^. Each FnI domain is approximately 40 amino acid residues in length and is involved in homotypic Fn interactions, as well as the binding of fibrin and collagen. Each FnII domain is approximately 60 amino acids residues in length and binds collagen while each FnIII domain is approximately 90 amino acids residues in length and binds Fn, heparin, and integrins^44^. All three Fn-domains are present in a variety of functionally diverse proteins among both prokaryotes and eukaryotes.

Tenascin C (TNC) is a large hexameric extracellular matrix (ECM) protein which is often co-expressed with Fn in response to tissue damage or inflammation^45^. Similar to Fn, over half of each TNC monomer is composed of FnIII domains; however unlike Fn, TNC does not have FnI or FnII domains. Group D streptococci bind to TNC, but the streptococcal binding protein is unknown^46^. Frick *et al*. reported that the surface-localized GAS adhesion protein known as protein H, which was originally identified as an immunoglobulin-binding protein, binds to FnIII domains present in both TNC and Fn^47^. These studies indicate that at least some isolates of Streptococci may adhere to cells and tissues by binding to host proteins containing FnIII domains such as TNC.

In this study, we found that the abundance of transcripts encoding proteins with FnIII-domains, including TNC, was greater in IAV-infected A549 cells compared to controls. We created a mutant strain that lacked the Fn-binding protein PrtF.2, and found that the parental strain, but not the mutant, bound to purified TNC. Finally, we showed the mutant strain was attenuated for virulence in a murine model of IAV superinfection compared to the wild-type strain.

## Results

### IAV infection of A549 cells increases TNC expression

Viral infection modulates host cells in a variety of ways, some of which lead to an increased susceptibility to bacterial infection^11,48–53^. To identify host cell changes in response to infection with IAV HK68 (H3N2), we compared gene expression between IAV-infected A549 cells and non-infected cells. Two independent experiments were completed. First, we infected A549 cells with 5 × 10^2^ TCID_50_ (low dose) IAV for 24 hours. Compared to uninfected cells, 403 genes of known function were differentially expressed ≥2-fold (Tables 1 and S1). Second we infected A549 cells with either 5 × 10^2^ TCID_50_ (low dose) or 10^5^ TCID_50_ (high dose) IAV for 24 hours. Compared to the controls, 441 and 978 genes of known function were differentially expressed ≥2-fold, respectively (Tables 1 and S1). Many of the changes in gene expression were consistent with results obtained in previous studies. For example, IAV infection was previously found to trigger the production of IFN-α/β and IFN-inducible genes^54^. Similarly, in our experiments the expression of interferon-inducible genes including *ifit1* (237-fold increase)*, ifit3* (146-fold increase)*, ifi44* (131-fold increase), and *ifi-15K* (98-fold increase) was increased in IAV-infected cells compared to controls. The expression of antiviral cytokines including *il29* (86-fold increase) and *il28a* (42-fold increase), as well as chemokines *ccl5* (4-fold increase), *cxcl10* (62-fold increase), and *ccl20* (30-fold increase) was also increased in IAV-infected A549 cells compared to controls. The results indicated that infection of A549 cells with HK68 resulted in a cellular response similar to that observed by other labs using H3N2 IAV strains other than HK68^55–57^.

**Table 1:** Summary of differentially expressed genes (≥2 fold) in response to infection with HK68 IAV in A549 cell lines at 24 hours post infection.

	EXPERIMENT 1	EXPERIMENT 2
5 × 10^2^ TCID_50_ IAV	363 (+)	401 (+)
	40 (−)	40 (−)
10^5^ TCID_50_ IAV		942 (+)
		35 (−)

The number of transcripts which were increased (+) or decreased (−) in IAV-infected A549 cells compared to uninfected A549 cells.

**Figure 1:**
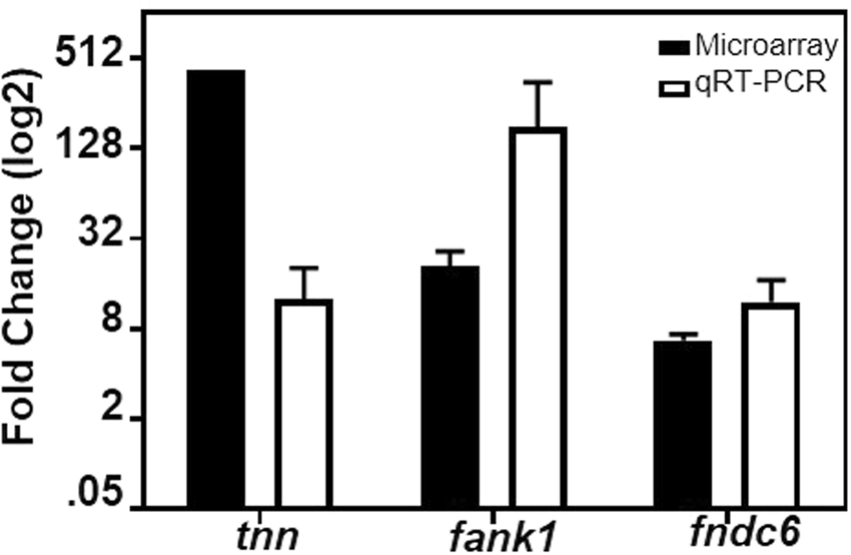
IAV infection of A549 cells increased expression of genes encoding FnIII domains. qRT-PCR (open bars) was used to measure transcripts of select genes that were differentially expressed in response to IAV infection (X-axis), as determined by using DNA microarrays (closed bars). The y-axis shows the fold-increase in transcripts associated with IAV-infected cells compared to uninfected controls. Transcripts encoding tenascin N (*tnn*), fibronectin type III and ankyrin repeat domains 1 (*fank1)*, and fibronectin type III domain containing 6 (*fndc6)* were measured. The means and sem are indicated.

We were particularly interested in the increased abundance of transcripts encoding proteins with FnIII-domains since a prior study^47^ showed FnIII-domains can facilitate the adherence of at least one strain of Streptococci. Our initial screen showed that expression of the genes *tnn* (432-fold increase), *fank1* (6.5-fold increase), and *fndc6* (21-fold increase) were among the most differentially expressed genes encoding FnIII domains. We confirmed the changes by using qRT-PCR (Fig. 1).

**Figure 2:**
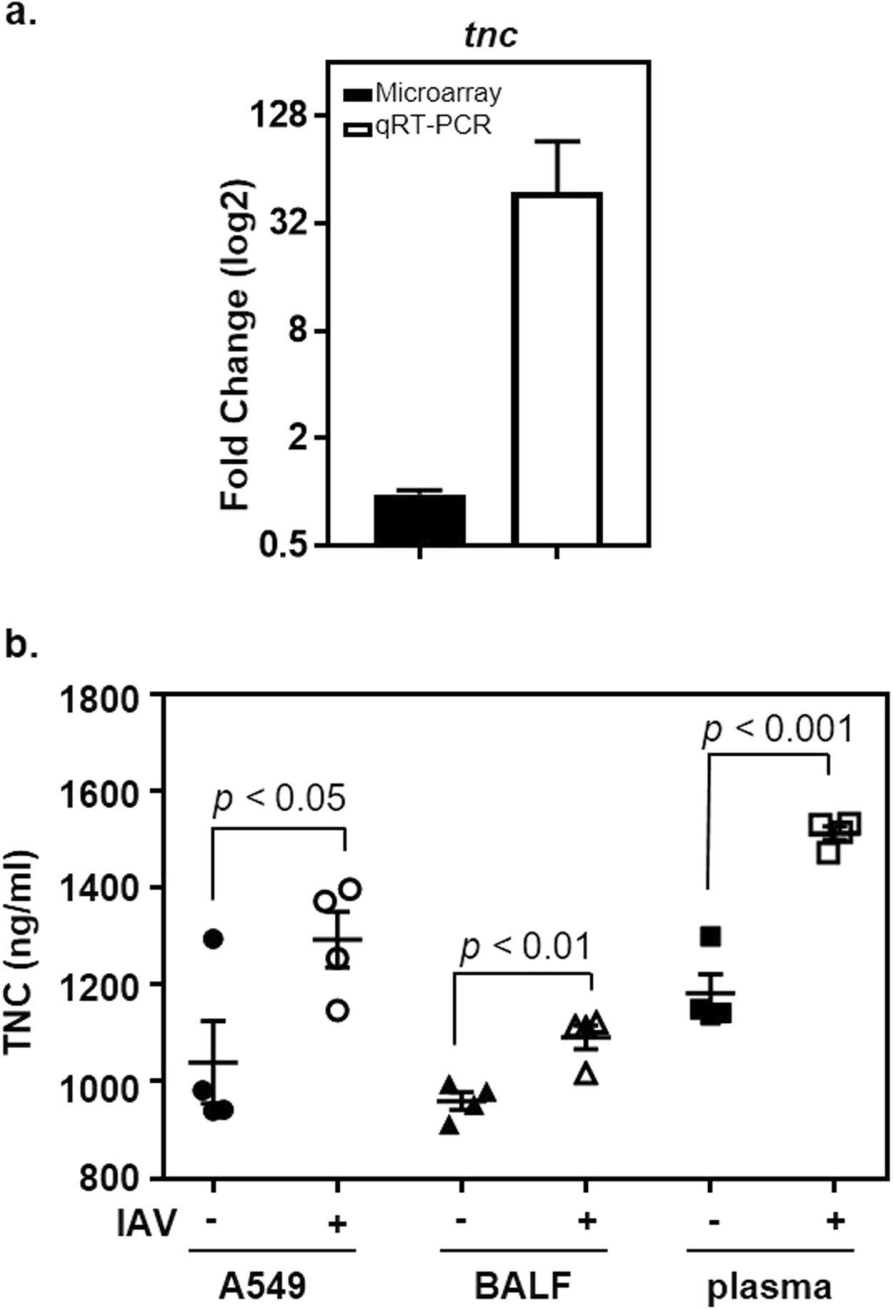
Increased expression of TNC following IAV infection of A549 cells. (**a**) The abundance of *tnc* transcripts was measured with qRT-PCR (open bars) 24 hours after IAV infection (IAV+) of A549 cells and compared to uninfected A549 cells (IAV−). The closed bar shows the results obtained with DNA microarrays. The y-axis shows the fold-increase in transcripts associated with IAV-infected cells compared to uninfected controls. (**b**) The abundance of TNC was measured in A549 cells 24 hours after IAV infection (IAV+) and compared to uninfected A549 cells (IAV−). Additionally, TNC was similarly measured in BALF and plasma collected from mice 8 days after being inoculated with IAV (IAV+; n = 4 mice) or allantoic fluid (IAV−; n = 4 mice). The mean and sem are indicated. A student’s t-test was used to measure statistical significance.

The tenascin family was of particular interest because the proteins have FnIII domains but lack FnI and FnII domains. Results with qRT-PCR showed that *tnn* transcripts (also known as *tnw*^58^) were approximately 13-fold more abundant in IAV-infected cells compared to controls (Fig. 1). While our screening revealed only a slight increase in the expression of the related gene *tnc*, it was of interest to measure its expression by qRT-PCR because previous reports indicated TNC was a ligand for Streptococcal adherence^46,47^. Results obtained with qRT-PCR showed that the expression of *tnc* was approximately 48-fold greater in IAV infected cells compared to uninfected controls (Fig. 2a), which was an increase even greater than that observed with *tnn*. Although it is unclear why there was a discrepancy in results obtained with arrays and qRT-PCR, qRT-PCR typically has greater specificity, sensitivity, and reproducibility^59–63^. Based on these results, we chose to explore the potential role of TNC in affecting the outcome of IAV-GAS superinfections.

To determine if the increase in *tnc* transcripts following IAV infection correlated with increased protein levels, we infected A549 cells with IAV, or not, for 24 hours and measured TNC using an enzyme linked immunosorbent assay (ELISA; Fig. 2b). The mean TNC level in uninfected A549 cells (IAV−) was 1,040 ± 171 ng/ml; in contrast, the level was significantly higher (p < 0.05; 1,293 ± 115 ng/ml) after IAV infection (IAV+; Fig. 2b). We also measured TNC in bronchoalveolar lavage fluid (BALF) and plasma obtained from mice 8 days after they were inoculated with either IAV or allantoic fluid as a control. The amount of TNC in BALF was greater in IAV-infected mice (1,092 ± 50 ng/ml) compared to the uninfected controls (960 ± 36 ng/ml; p < 0.01). Similarly, the amount of TNC in plasma was greater in IAV-infected mice (1,512 ± 28 ng/ml) compared to uninfected controls (1,182 ± 79 ng/ml; p < 0.001). Thus, while the magnitude of the increase in TNC abundance following IAV infection did not directly correlate with that of the corresponding transcripts for reasons that are not known, we did observe statistically significant increases in TNC in IAV infected A549 cells, as well as IAV infected mice, compared to uninfected controls.

### IAV infection of A549 cells increases the adherence of MGAS315

**Figure 3:**
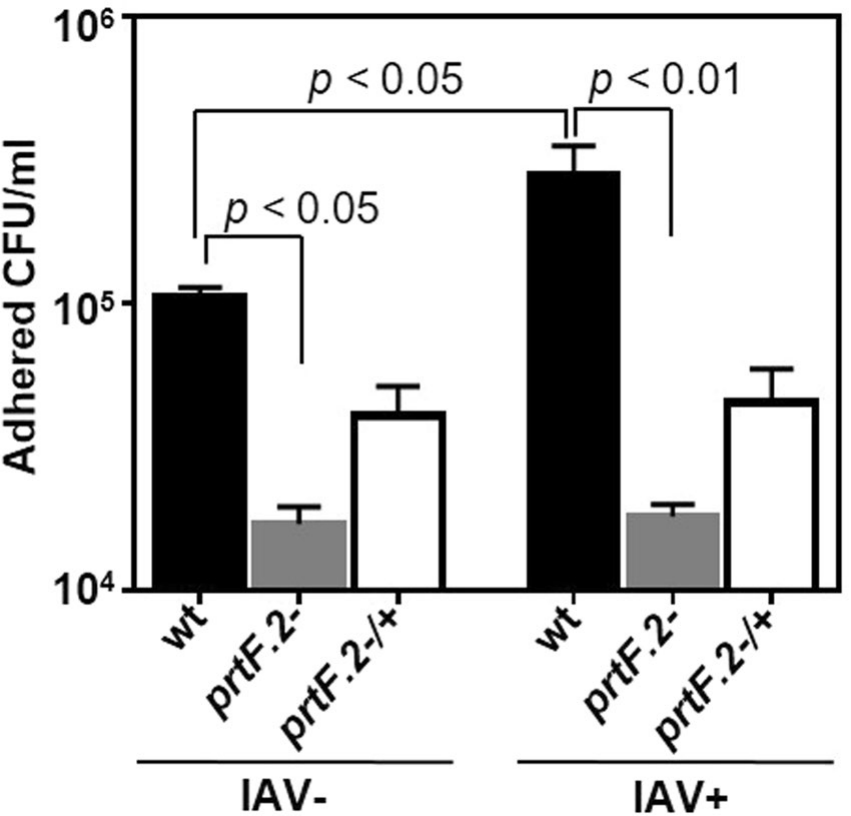
Inactivation of *prtF.2* decreased the adherence of GAS to IAV-infected A549 cells. The number of wild-type (wt), *prtF.2* mutant (*prtF.2*^−^), or the complemented *prtF.2* mutant (*prtF.2*^−/+^) bacteria adhered to A549 cells following 24 hours of IAV infection (IAV+) or not (IAV−) was determined by dilution plating. The means and sem are reported. Two-way ANOVA with Tukey’s post-test was used to measure the significance of the differences among the strains.

The increased expression of proteins with FnIII domains in IAV-infected A549 cells suggested a potential means for enhanced GAS adherence following viral infection. Multiple Fn-binding proteins have been characterized in GAS; however, PrtF.2 is the predominant Fn-binding protein in strain MGAS315^64^. We inactivated the *prtF.2* gene and measured the effect on adherence to IAV-infected A549 cells and uninfected controls. We also complemented the mutant by transforming it with a shuttle plasmid that expressed the *prtF.2* open reading frame from a well characterized GAS promoter (P_*rofA*_)^39,65–67^. A549 monolayers were infected with IAV, or not, for 24 hours and the MGAS315 wild-type strain, the *prtF.2* mutant strain (*prtF.2*^−^), or the *prtF.2* complemented mutant strain (*prtF.2*^−/+^) was added and incubated for 1 hour to allow the bacteria to bind to the monolayers. After extensive washing, attached and internalized bacteria were quantified by dilution plating. Among uninfected A549 cells (IAV−), an average of 1.1 × 10^5^ CFU/ml were recovered following the addition of wild-type GAS compared to 0.17 × 10^5^ CFU/ml following the addition of the *prtF.2* mutant strain (p < 0.05; Fig. 3). The results were consistent with previous work showing that PrtF.2 contributes to GAS adherence to epithelial cells^35,41^. Among IAV-infected (IAV+) A549 cells, 2.8 × 10^5^ CFU/ml were recovered following the addition of wild-type GAS compared to 0.18 × 10^5^ CFUs following the addition of the *prtF.2* mutant strain (*p* < 0.01; Fig. 3). Complementation of the mutant strain partially increased adherence, although not to the level of the parental strain and increased adherence to IAV infected cells was not observed for reasons that are not known (Fig. 3). The results indicated that the adherence of wild-type GAS was greater than that of the *prtF.2* mutant in the absence of IAV infection (6.5-fold increase), and that the difference was even more pronounced among IAV-infected cells (15.6-fold increase). As previously reported, adherence of GAS to IAV-infected A549 cells was also greater compared to adherence to cells not infected with IAV (*p* < 0.05) (Fig. 3)^13,68^. Together, the results confirmed that GAS adherence was partially dependent on PrtF.2 and that a preceding IAV infection increased adherence, some of which was dependent on PrtF.2.

### PrtF.2 is required for GAS adherence to immobilized TNC

**Figure 4:**
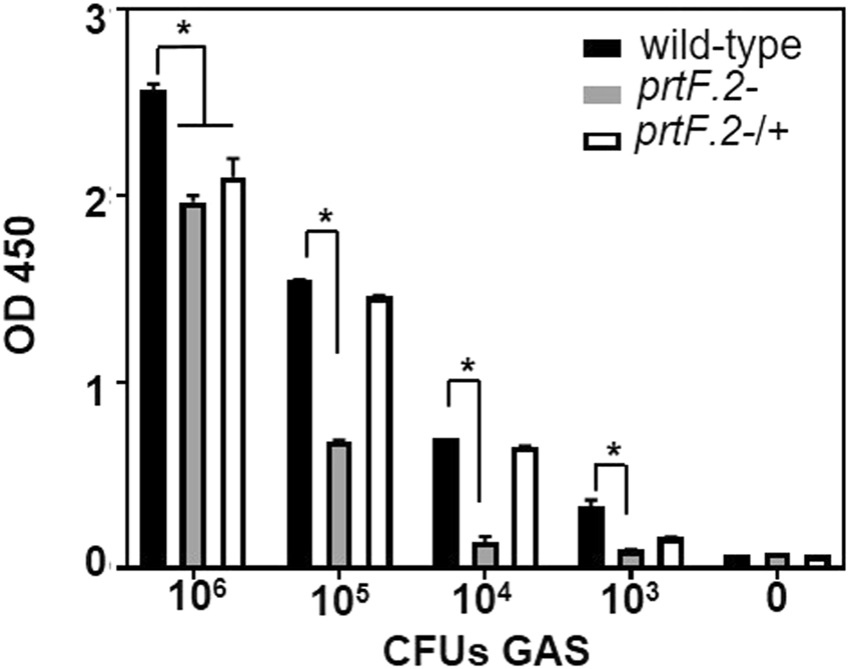
Inactivation of *prtF.2* decreased GAS binding to immobilized TNC. The wild-type (wt), *prtF.2* mutant (*prtF.2*^−^), or the complemented *prtF.2* mutant strain (*prtF.2*^−/+^) was incubated with immobilized TNC. After extensive washing, bound bacteria were detected with ELISA using antibody specific to GAS. The data are reported as the means and sem. Two-way ANOVA with Tukey’s post-test was used to measure the significance (*p < 0.001) of differences among the strains.

Proteins with FnI-domains, such as Fn, bind PrtF.2^35,41^. To determine if PrtF.2 can also bind proteins composed mainly of FnIII-domains, we investigated the binding of PrtF.2 to TNC. Purified TNC was immobilized to microtiter plates prior to adding serial dilutions of either the wild-type MGAS315 strain, the *prtF.2* mutant (*prtF.2*^−^), or the complemented mutant strain (*prtF.2*^−/+^). After extensive washing, adherent bacteria were detected by ELISA with an antibody specific to GAS. Wild-type GAS bound to TNC-coated plates, but not to control wells (Fig. 4), and much of the binding was dependent on PrtF.2 (Fig. 4). The binding of the *prtF.2* mutant was significantly lower than the wild-type at all concentrations of GAS tested (p > 0.001). Complementation of the *prtF.2* mutant increased GAS binding to TNC-coated plates compared to the mutant strain, although binding was not restored to levels observed with the parental strain. The results indicated that GAS bound to purified, immobilized TNC and binding was largely, but not solely, dependent on PrtF.2 (Fig. 4).

### GAS co-localizes with TNC expressed by A549 cells

**Figure 5:**
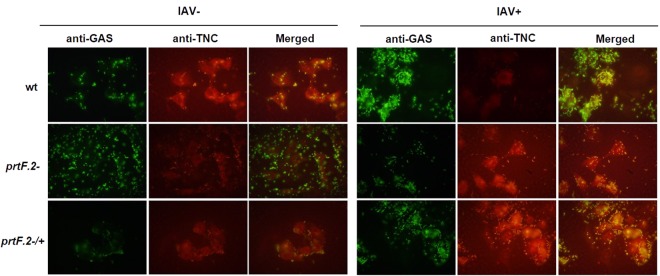
Inactivation of *prtF.2* decreased co-localization of GAS to TNC expressed by A549 cells. The co-localization of adhered MGAS315 wild-type (wt), *prtF.2* mutant (*prtF.2*^−^), or the complemented *prtF.2* mutant strain (*prtF.2*^−/+^) to TNC expressed by A549 cells infected with IAV (IAV+) or not (IAV−) was determined. Cells were fixed and indirect immunofluorescence was used to detect GAS (green) or TNC (red), with antibodies specific to each protein. Coverslips were imaged with an Olympus BX 60 fluorescent scope (60X magnification) with a Nikon DS camera. Co-localization of GAS and TNC was detected in the merged images.

To determine if GAS co-localized with TNC expressed by A549 cells, we first infected A549 cells with IAV, or not, for 24 hours and then added the wild-type MGAS315, the *prtF.2* mutant (*prtF.2*^−^), or the complemented mutant (*prtF.2*^−/+^*)* strain for 1 hour. The monolayers were extensively washed and adherent GAS and A459 cells were detected using indirect immunofluorescence with antibodies specific to either GAS or TNC, respectively. The Pearson correlation coefficient (PCC) was used to quantify the degree of co-localization between fluorophores^69^. The coefficient describes how well the red and green pixels are related by a linear equation with 1 being a perfect relationship and 0 being no co-localization relationship between the two fluorophores. Among A549 cells infected with wild-type GAS, the degree of co-localization among TNC and GAS was greater in cells infected with IAV (PCC = 0.82) compared to uninfected control cells (PCC = 0.74; Fig. 5). Interestingly, co-localization was also observed with IAV-infected (IAV+) cells incubated with the *prtF.2* mutant strain (PCC = 0.71) but less so with uninfected cells (PCC = 0.572) (Fig. 5). The results indicated that GAS proteins in addition to PrtF.2 can mediate co-localization of GAS to TNC. As observed in previous experiments, complementation of the *prtF.2* mutant partially restored the phenotype associated with the parental strain (PCC = 0.81 and 0.67 among IAV infected and uninfected cells, respectively).

### The prtF.2 mutant is less virulent in a murine model of IAV-GAS superinfection

**Figure 6:**
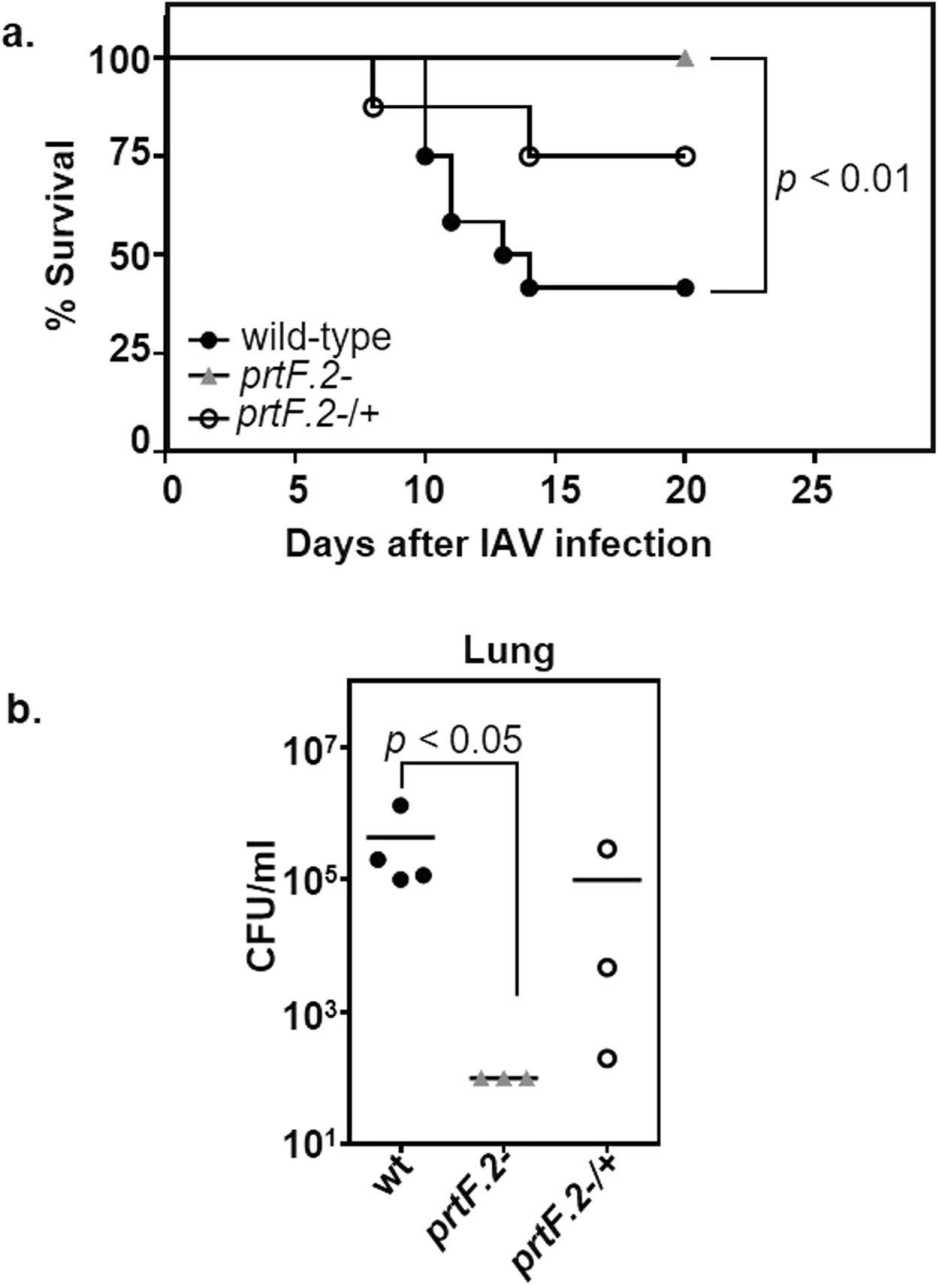
Inactivation of *prtF.2* decreased the mortality of IAV-GAS superinfected mice and decreased the abundance of GAS in the lower respiratory tract. (**a**) Mice were infected on day 0 with IAV and on day 7 with wild-type GAS (●; n = 12 mice), the *prtF.2* mutant (▲; n = 8 mice), or the complemented *prtF.2* mutant strain (○; n = 9 mice). The mortality is indicated as the percentage of mice surviving the IAV-GAS superinfection. A Kaplan Meier survival analysis was used to measure the significance of differences in mortality among the groups. (**b**) The number of viable bacteria in the lungs of mice infected on day 0 with IAV and on day 7 with GAS was determined 24 hours after inoculation with GAS by dilution plating. Groups of mice were superinfected with wild-type GAS (●; n = 4 mice), the *prtF.2* mutant (▲; n = 3 mice), or the complemented *prtF.2* mutant strain (○; n = 3 mice). The mean values are indicated. A non-parametric one-way ANOVA with Kruskal Wallis test was used to measure the significance of differences among the strains.

Our results suggested that PrtF.2 was important in GAS interactions with IAV-infected host cells *ex vivo*; however, it was difficult to ascertain the overall significance of PrtF.2 to the outcome of an IAV-GAS superinfection. This was due, in part, to the finding that the *prtF.2* mutant adhered substantially (>10^4^ CFU/ml) to both IAV-infected cells and controls (Fig. 3) and co-localized with TNC expressed by A549 cells infected with IAV (Fig. 4). Therefore, we compared the virulence of the *prtF.2* mutant strain to the isogenic parental strain using an established murine model of IAV-GAS superinfection^70–72^. Groups of mice were first inoculated with a sub-lethal dose of IAV (0.1 LD_50_). When the mice had recovered from the IAV infection (7 days after IAV inoculation), mice were inoculated with a sub-lethal dose (0.1 LD_50_) of either wild-type GAS, the *prtF.2* mutant (*prtF.2*^−^), or the complemented mutant strain (*prtF.2*^−/+^). Only 42% of mice superinfected with IAV and the wild-type strain survived (Fig. 6a). In contrast, 100% of the mice infected with IAV and the *prtF.2* mutant strain survived, which indicated that PrtF.2 contributes to the virulence of GAS in an IAV superinfection (*p* < 0.01). 75% of mice superinfected with the complemented *prtF.2* mutant strain survived, again indicating that complementation partially restored the parental phenotype.

Finally, we also quantified the number of viable GAS in the lungs of superinfected mice 24 hours after GAS inoculation (8 days after IAV inoculation) using groups of 3 to 4 mice, which was previously shown to be a sufficient number to obtain statistically significant results^71^. A sizeable number of wild-type GAS were recovered from the IAV superinfected mice (4.3 × 10^5^ CFU/ml; Fig. 6b). In contrast, less than 100 CFU/ml (the limit of detection) were recovered from the lungs of mice inoculated with IAV and the *prtF.2* mutant strain (*p* < 0.05). An average of 0.98 × 10^5^ CFU/ml were recovered from the lungs of mice inoculated with IAV and the complemented strain. The results support the idea that PrtF.2 contributes to the ability of GAS to colonize and increase mortality in a host previously infected with IAV.

## Discussion

IAV infections create permissive conditions for subsequent bacterial superinfections, which significantly increase the mortality associated with both IAV epidemics and pandemics^73^. To investigate the host responses to IAV infection that may contribute to this phenomenon, we screened IAV-infected A549 cells for changes in gene expression likely to promote GAS colonization. The most pronounced changes included increased expression of genes encoding proteins with FnIII domains such as *tnn, tnc, fank1*, and *fndc6*. Here, we focused on *tnc* and showed that TNC expressed by A549 cells co-localized with adherent GAS. Furthermore, GAS expressing PrtF.2 bound more efficiently to immobilized TNC compared to an isogenic *prtF.2* mutant and inactivation of *prtF.2* significantly decreased the virulence of GAS, as determined using a murine model of IAV superinfection. Together, the results suggest that the increased production of TNC in response to IAV may enhance GAS colonization and the severity of infection and that the surface localized protein PrtF.2 contributes to the process.

Elevated TNC expression occurs during inflammation, often in conjunction with Fn production^74^. This is prominent in the inflamed bronchi of patients with interstitial lung disease or pneumonia^75–79^. Increased TNC expression also occurs in response to tissue damage, such as that associated with IAV infection^80–82^. TNC and Fn are both damage-associated molecular patterns (DAMPs), which can potentiate tissue damage by increasing the synthesis of proinflammatory cytokines^83^. Thus, increased TNC expression in response to IAV infection is likely to both enhance bacterial colonization and increase immune-mediated tissue damage, thereby exacerbating disease^2,49^.

A number of studies using RNA*seq*, microarrays, and quantitative proteomics identified changes in gene or protein expression following IAV infection (reviewed in^84^). Often these studies focused on changes among genes associated with the immune response and not those likely to directly affect host-cell interactions with bacteria^56,57,85–89^. However, a study by Lietzen *et al*. characterized changes in protein abundance among primary human macrophages following IAV infection using H3N2 strains (A/Udorn/1972 and A/Beijing/353/89)^90^ and showed that IAV infection is associated with a robust increase in the expression of various DAMPs, including Fn^90^. The results, and ours, are consistent with several other reports of increased Fn expression following IAV infection^12,16–18,25,91^.

In addition to Fn and TNC, transcripts encoding other GAS ligands were more abundant after IAV infection (Table S1). These included alpha-2-macroglobulin (30-fold increase), which is a plasma protein that inhibits a variety of mammalian proteases. Alpha-2-macroglobulin binds to a GAS surface-localized protein known as GRAB (protein G-related alpha-2-macroglobulin-binding protein)^92,93^, thereby inhibiting proteolytic degradation of various GAS adhesins and other surface-localized proteins^92,93^. In addition, transcripts encoding proteins with various collagen domains were more abundant following IAV infection of A549 cells compared to uninfected controls. GAS encodes several different collagen-binding proteins and binding to host collagen types I and IV is an important mechanism of adherence^94,95^. IAV infection also increased (218-fold) the expression of bone marrow stromal cell antigen 2 (BST2/tetherin). BST2 blocks the release of IAV from host cells by binding, or tethering, nascent virions to the membranes of infected cells^96^. Since GAS (and *S. pneumonia*)^16,17,25,97^ bind directly to IAV neuraminidase, progeny virus tethered to the host cell membrane can also promote GAS adherence^2,25^. Thus, while we focused on TNC interactions with PrtF.2, the results indicated that additional proteins that are also likely to facilitate GAS adherence were more abundant following IAV infection.

Several studies have reported an increase in bacterial adherence to IAV-infected cells compared to uninfected cells. In particular, the increased abundance of Fg and Fn following IAV infection clearly enhances GAS adherence in a manner thought to be largely mediated by the surface localized M protein^13,68,98–100^. Similar to many bacterial pathogens, GAS adheres to host cells and tissues by utilizing a variety of receptors and adhesins. Many GAS ligands are ECM proteins including Fn, Fg, collagen, and laminin^101^. Our results contribute to this model and show that another ECM protein, TNC, also facilitates GAS adherence, in part, by binding to PrtF.2.

Our results build on a theme whereby the host response to IAV infection, including the production of a variety of ECM proteins, creates an environment rich in host ligands for GAS adherence. In addition, the increase in soluble ECM proteins can also increase GAS virulence following the recruitment, or binding, of the proteins to the GAS cell surface. We recently showed that the binding of Fg and albumin, which are more abundant following IAV infection, to the surface GAS increased the mortality of mice in an IAV-GAS superinfection^71^. Thus, the host response to IAV infection is likely to result in increased GAS colonization and persistence through bacterial interactions with multiple host proteins that are elevated in response to viral infection.

Our profile of the transcriptome response of A549 cells to IAV infection showed an increase in the expression of transcripts encoding several proteins known, or suspected, to be ligands for GAS adherence. We focused on the significance of the host ECM protein TNC and the GAS surface-localized protein PrtF.2. Our results highlight the manner in which an increase in the production of ECM proteins, such as TNC, in response to IAV infection is likely to promote bacterial colonization and contribute to the mortality associated with IAV superinfection.

## Materials and Methods

### E. coli and GAS culture conditions

*S. pyogenes* strain MGAS315, serotype M3 (ATCC) was grown statically with Todd-Hewitt broth (BD Biosciences, San Jose, CA) supplemented with 0.2% yeast extract (THY) at 37 °C in 5% CO_2_. Routinely, GAS was grown from frozen stocks overnight with THY agar plates at 37 °C with 5% CO_2_. Colonies were inoculated into pre-warmed THY medium and grown to mid-exponential phase (OD_600_ = 0.5). When appropriate, the *prtF.2* mutant, or complemented mutant strains, were grown with THY containing erythromycin (2.5 μg/ml) or kanamycin (500 μg/ml), respectively. *Escherichia coli* strain DH5α (Gibco; Gaithersburg, MD) was grown with Luria Bertani (LB) medium. When appropriate, erythromycin (200 μg/ml) was added to LB broth.

### GAS mutagenesis

The *prtF.2* gene was insertionally inactivated, essentially as previously described^102^. The *prtF.2* gene was amplified using the primers PrtF2_F1 (5′-GCTTGAATTCAAACGACAGTTCCGGCTGAT-3′) and PrtF2_R1 (5′-GCTTAAGCTTTCAGGTCATCCACTGCTAC-3′). The PCR product was digested with *EcoRI* and *HindIII* resulting in a 350 bp internal fragment that was gel purified and cloned into the vector pVA981-2, which replicates episomally in *E. coli* but not in *S. pyogenes*^103^. Following transformation of *E. coli* strain DH5α (Gibco-BRL, USA), the recombinant plasmid, designated pVA981-2*::prtF.2*, was isolated and electroporated into strain MGAS315. Transformants were selected with THY agar plates containing erythromycin (2.5 μg/ml) and insertional inactivation of *prtF.2* was verified with PCR. The ORF downstream of *prtF.2* is transcribed in the opposite direction compared to *prtF.2* and the ORFs are separated by 264 bp of non-coding DNA. The *prtF.2* mutant strain was complemented with the shuttle plasmid pAH1, which was derived from the streptococcal expression plasmid pMNN23^65^. To do so, the *ropB* ORF in pMNN23 was excised with *BamH*1 and *Pst*1. In its place, a DNA fragment consisting of the *prtF.2* ORF was cloned. The resulting shuttle plasmid consisted of the *prtF.2* ORF cloned adjacent, and downstream, of the *rofA* promoter. The native, or chromosomal *rofA* promoter controls the expression of the surface localized fibronectin protein Protein F or PrtF^104^. The recombinant plasmid construct was confirmed by DNA sequencing. pAH1 was purified and electroporated into the MGAS315 *prtF.2* mutant strain. Transformants were selected with THY agar plates containing kanamycin. The growth curves of the MGAS315 wild-type, *prtF.2* mutant, and the complemented mutant strains were identical (Supplementary Fig. 1).

### IAV virus strain

Influenza virus (A/Hong Kong/1/68-H3N2; HK68) was created using the PR8 reverse genetic system as described^105^ and propagated at 35 °C for 72 hours in the allantoic cavities of 10-day-old embryonated chicken eggs^72,105^. As described previously^72^, virus were sequenced and titers were determined by TCID_50_ (50% tissue culture infectious dose) using MDCK (Madin-Darby Canine Kidney Epithelial Cells).

### Total RNA isolation

Monolayers of human adenocarcinomic alveolar epithelial (A549) cells (6 × 10^5^ cells/ml) were infected with IAV at 5 × 10^2^ TCID_50,_ or 10^5^ TCID_50_ IAV. After one hour, the inoculum was removed and the cells were washed twice with PBS and incubated with media containing 1 µg/ml TPEC trypsin for 24 hours at 37 °C and 5% CO_2_. Total RNA was extracted from control A549 cells and IAV-infected A549 cells at 24 hours post-IAV infection using Trizol reagent (Invitrogen Life Technologies, Carlsbad, CA, USA). RNA was purified with the RNeasy kit (Qiagen, Valencia, CA). RNA purity and quantity was evaluated with Agilent chips (Agilent RNA 600 Nano kit).

### Microarray data analysis

Three independent microarray experiments were done with Codelink Whole Human Genome Bioarrays from Applied Microarrays (Tempe, AZ), as previously described^106^. Briefly, semi-confluent A549 monolayers were infected with 5 × 10^2^ TCID_50_, (low dose)_,_ 10^5^ TCID_50_ IAV (high dose), or mock infected (no virus) for 24 hours and RNA was extracted. To identify differently expressed genes, we normalized data (test/reference) of IAV-infected cells (test) and control treated A549 cells (reference). Differentially expressed genes at the cut-off of fold change ≥2 are listed in Table S1.

### Quantitative reverse transcriptase - PCR

Quantitative reverse transcriptase-PCR (qRT- PCR) was done using Power SYBR Green PCR master mix (Applied Biosystems) and an ABI 7300 thermocycler (Applied Biosystems). The comparative threshold cycle (*C*_*t*_) method was used to quantitate transcript abundance. The ΔΔ*C*_*t*_ value was calculated by difference in normalized *C*_*t*_ value (Δ*C*_*t*_) from infected (IAV+) samples to the Δ*C*_*t*_ from non-infected (IAV−) samples. The ΔΔ*C*_*t*_ value was transformed into 2^ΔΔ*Ct*^ value as the estimated gene expression fold change value. Glyceraldehyde 3-phosphate dehydrogenase (*gapdh)* was used as an internal control. qRT- PCR was done in duplicate with 5 independently isolated RNA samples.

### Enzyme linked immunosorbent assays (ELISA)

Enzyme linked immunosorbent assays (ELISA) were completed as described previously^107^. 96-well plates were coated with 5 μg/mL TNC diluted with 0.1 M sodium carbonate (pH 9.8). Plates were blocked with 1% BSA, washed using PBS containing 0.05% Tween 20 (PBS-T), and serial dilutions of GAS cells (wild-type MGAS315, the *prtF.2* mutant, or the complemented mutant strain) were added and incubated for 2 hours at 37 °C. Plates were washed again with PBS-T, and anti-GAS antibody (ThermoFisher; 1:1000 dilution) added and incubated for 1 hour at room temperature. After washing with PBS-T, HRP-conjugated goat anti-rabbit IgG (H + L) (1:10,000 dilution) (Sigma, St. Louis, MO) was added to each well and incubated for 1 hour at room temp. After washing, One-Step-TMB Turbo substrate (Thermo Scientific, Rockford, IL) was added and the OD measured at 450 nm using a Biotek EL808 plate reader (Biotek, Winooski, VT). End-point titers are presented as the reciprocal serum dilution corresponding with the last well demonstrating an OD_450_ of 0.1 in the titration curve.

To quantify TNC expression in A549 cell-culture supernatants, mouse BALF and plasma, an ELISA was used according to the manufactures instructions (Abcam; Cambridge, United Kingdom). Briefly, A549 cells were infected with 5 × 10^2^ TCID_50_ IAV or mock infected (no virus) for 24 hours and whole-cell lysates were collected. For animal fluids, mice were infected with 0.1 LD_50_ IAV or allantoic fluid for 8 days and fluids were collected. 100 µl of purified TNC or 100 µl (diluted 1:10 or 1:100) of each sample was added to anti-TNC pre-coated microtiter plates and incubated for 90 minutes at 37 °C. Biotinylated-TNC antibodies (diluted 1:100) were then added for 60 minutes at 37 °C; plates were washed 3 times with PBS-T and avidin-biotin-peroxidase complex (diluted 1:100) was added. Plates were again washed thoroughly and 90 µl TMB substrate was added and color allowed to develop for 20 minutes. OD was measured at 450 nm using a Biotek EL808 plate reader (Biotek, Winooski, VT).

### GAS attachment to A549 cells

A549 cell monolayers (6 × 10^5^ cells/ml) were washed thoroughly with PBS and infected with 5 × 10^2^ TCID_50_ IAV. After 1 hour the viral inoculum was removed, the cells were washed, and media containing TPEC trypsin (1 µg/ml) added and the cells incubated at 37 °C with 5% CO_2_. After 24 hours, cells were again washed and GAS diluted with culture media to a multiplicity of infection (MOI) of 100 (6 × 10^7^ CFU’s) added. Monolayers were centrifuged at 200 × *g* for 10 minutes to facilitate contact between GAS and the A549 cells and incubated for 60 minutes at 37 °C with 5% CO_2_. Monolayers were then washed to remove non-adherent bacteria. The number of adherent and intracellular bacteria was enumerated after detachment of cells with 0.25% trypsin-EDTA for 1–2 minutes at 37 °C with 5% CO_2_ followed by serial dilution plating with THY agar plates. The results from 3 independent experiments, each done in duplicate, are shown.

### Microscopy

A549 cell monolayers (6 × 10^5^ cells/ml) were infected with 5 × 10^2^ TCID_50_ IAV for 24 hours and then fixed with 100% ethanol for 20 minutes at −20 °C. Coverslips were incubated with primary antibodies against TNC (ThermoFisher; dilution 1:1000), Fn (Abcam; Cambridge, United Kingdom; dilution 1:1000), or GAS (ThermoFisher; dilution 1:5000), followed by incubation with the appropriate secondary antibody conjugated to DyLight fluors (Jackson ImmunoResearch Laboratories; West Grove, PA; dilution 1:10,000). Coverslips were mounted onto glass slides and imaged with an Olympus BX 60 fluorescent scope (60X magnification) with a Nikon DS camera. The integrated density was calculated from the fluorescent intensity and area of the A549 cells which was determined with ImageJ v1.48 (National Institutes of Health, Bethesda, MD). The integrated density of the background (shape similar in size to A549 cells) was measured and subtracted from cell measurements. A total of 17–25 fields of view were imaged from 3 independent experiments each done with duplicate coverslips.

### Murine model of infection

All experiments were conducted in conformity with the recommendations in the Guide for the Care and Use of Laboratory Animals of the National Institutes of Health. Experiments completed under animal protocol number 11-07015-18E were approved by the local animal care committee (Institutional Animal Care and Use Committee) of the University of South Dakota. Female (6 to 8-week-old) BALB/c mice were purchased from Harlan Laboratories (Indianapolis, IN) and housed in groups of four with 24-hour access to food and water. The IAV-GAS superinfection model was previously described^70^. Briefly, mice were anesthetized with isoflurane and inoculated intranasally with 100 µl of a sub-lethal dose [0.1 LD_50_ (10^4.75^ TCID_50_)] of IAV at day 0. Seven days later anesthetized mice were inoculated intranasally with 100 µl of a sub-lethal dose [0.03 LD_50_ (10^6^ CFU)] of GAS. Experiments were completed using 8–12 mice per group. BALF and plasma were collected from mice as described previously^71^.

### GAS quantification in the lungs of mice

To enumerate viable GAS present in the lungs of mice, groups of 4 mice were euthanized 24 hours after inoculation with MGAS315 and lungs collected. GAS were enumerated by dilution plating on blood agar (TSA with 5% sheep blood) plates as previously described^70^.

### Statistics and image production

All quantification and statistical analyses of data was performed with GraphPad Prism 6 software. Statistical analyses included a one-way or two-way analysis of variance (ANOVA) with a Tukey’s multiple comparisons post-hoc test, Kaplan Meier survival analysis, or a Student’s t-test, as appropriate.

### Data availability

Datasets (Raw and processed data) generated and analyzed during the current study are available from NCBI Gene Expression Omnibus (GEO) under accession number GSE112215.

## Electronic supplementary material

Supplementary Fig. 1

Supplementary Table 1
