# Supplementary material for: The Streptococcus pyogenes fibronectin/tenascin-binding protein PrtF.2 contributes to virulence in an influenza superinfection
Source: Sci Rep. 2018 Aug 14;8:12126. doi: 10.1038/s41598-018-29714-x (PMC6092322; doi:10.1038/s41598-018-29714-x)

The *Streptococcus pyogenes* fibronectin/tenascin-binding protein PrtF.2 contributes to virulence in an influenza superinfection

Andrea L. Herrera, Haddy Faal, Danielle Moss, Leslie Addengast, Lauren Fanta, Kathleen Eyster, Victor C. Huber, and Michael S. Chaussee*

**Supplementary Figure 1. Inactivation of *prtF.2* does not affect GAS growth.**

Strains were cultured with THY broth with or without 2 µg/ml erythromycin (ERM) or 500 µg/ml kanamycin (KAN). Growth was measured by determining the absorbance at 600 nm at various time points. The data are the means two independent experiments.


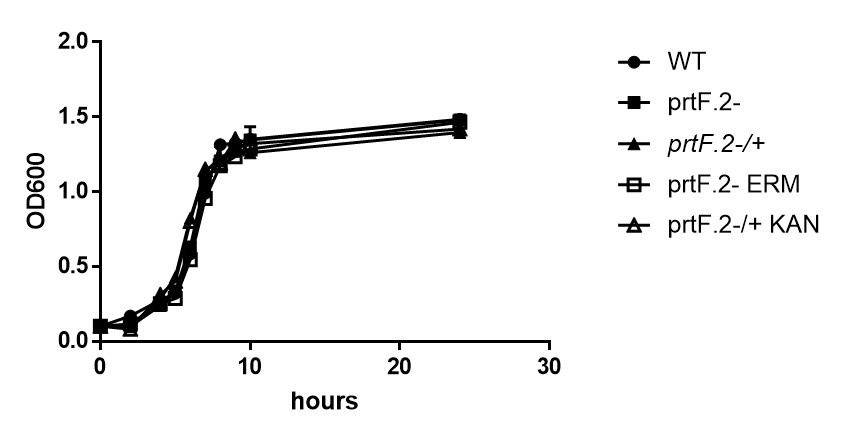

Supplement: Supplementary file 1 — Supplementary Fig. 1 [file 41598_2018_29714_MOESM1_ESM.docx]
