# Supplementary material for: The Streptococcus pyogenes fibronectin/tenascin-binding protein PrtF.2 contributes to virulence in an influenza superinfection
Source: Sci Rep. 2018 Aug 14;8:12126. doi: 10.1038/s41598-018-29714-x (PMC6092322; doi:10.1038/s41598-018-29714-x)
Supplement: Supplementary file 2 — Supplementary Table 1 [file 41598_2018_29714_MOESM2_ESM.docx]

The *Streptococcus pyogenes* fibronectin/tenascin-binding protein PrtF.2 contributes to virulence in an influenza superinfection

Andrea L. Herrera, Haddy Faal, Danielle Moss, Leslie Addengast, Lauren Fanta, Kathleen Eyster, Victor C. Huber, and Michael S. Chaussee*

**Supplementary Table 1:** Differentially expressed genes (> 2 fold) in response to infection with HK68 IAV in A549 cell lines at 24 hours post infection. Results are sorted from the highest to lowest increased expression per experiment. (FC= fold change)

| Experiment 1  5 X 10^2^ TCID_50_ IAV | | Experiment 2  5 X 10^2^ TCID_50_ IAV | | Experiment 2  10^5^ TCID_50_ IAV | |
| --- | --- | --- | --- | --- | --- |
| Description | **FC** | **Description** | **FC** | **Description** | **FC** |
| tenascin N (TNN) | 423 | interferon, alpha 14 (IFNA14) | 151 | interferon-induced protein with tetratricopeptide repeats 1 (IFIT1), transcript variant 2 | 237 |
| cyclic nucleotide binding domain containing 1 (CNBD1), mRNA | 199 | family with sequence similarity 71, member A (FAM71A) | 65 | bone marrow stromal cell antigen 2 (BST2) | 219 |
| TNFAIP3 interacting protein 3 (TNIP3) | 154 | small proline-rich protein 2A (SPRR2A) | 52 | N-acetyltransferase 8 (camello like) (NAT8) | 190 |
| tryptophan hydroxylase 1 (tryptophan 5-monooxygenase) (TPH1) | 70 | N-acetylated alpha-linked acidic dipeptidase-like 1 (NAALADL1) | 51 | tripartite motif-containing 22 (TRIM22) | 190 |
| spectrin, beta, non-erythrocytic 2 (SPTBN2) | 40 | FSHD region gene 2 protein (FRG2) | 46 | 2'-5'-oligoadenylate synthetase-like (OASL), transcript variant 1 | 177 |
| carbonic anhydrase XIV (CA14) | 31 | myosin binding protein C, cardiac (MYBPC3) | 37 | interferon-induced protein with tetratricopeptide repeats 3 (IFIT3) | 146 |
| ataxin 2-binding protein 1 (A2BP1), transcript variant 3 | 31 | regulator of G-protein signalling 18 (RGS18) | 36 | interferon-induced protein 44 (IFI44) | 131 |
| alpha-2-macroglobulin (A2M) | 30 | centrosome spindle pole associated protein (CSPP) | 35 | interferon, alpha-inducible protein (clone IFI-15K) (G1P2) | 99 |
| GTPase, IMAP family member 2 (GIMAP2) | 29 | F-box and leucine-rich repeat protein 19 (FBXL19) | 33 | chemokine (C-C motif) ligand 5 (CCL5) | 90 |
| zinc finger protein 482 (ZNF482) | 29 | sialic acid binding Ig-like lectin 12 (SIGLEC12), transcript variant 2 | 30 | interleukin 29 (interferon, lambda 1) (IL29) | 86 |
| 26 serine protease (P11) | 24 | cold autoinflammatory syndrome 1 (CIAS1), transcript variant 1 | 30 | interferon, alpha 1 (IFNA1) | 80 |
| proteasome (prosome, macropain) subunit, alpha type, 8 (PSMA8), transcript variant 1 | 21 | leucine rich repeat containing 2 (LRRC2) | 28 | interferon, alpha-inducible protein 27 (IFI27), transcript variant a | 70 |
| olfactory receptor, family 6, subfamily A, member 2 (OR6A2) | 20 | tyrosine aminotransferase (TAT), nuclear gene encoding mitochondrial protein | 28 | interferon regulatory factor 7 (IRF7), transcript variant d | 66 |
| partial mRNA for keratin associated protein 43 (KRTAP4.3 gene) | 20 | fibronectin type III domain containing 6 (FNDC6) | 26 | lectin, galactoside-binding, soluble, 9 (galectin 9) (LGALS9), transcript variant long | 66 |
| sulfatase 1 (SULF1) | 18 | ankyrin repeat and SOCS box-containing 17 (ASB17) | 26 | chemokine (C-X-C motif) ligand 10 (CXCL10) | 62 |
| dystonin (DST), transcript variant 1 | 17 | erythrocyte membrane protein band 41 like 4A (EPB41L4A) | 24 | myxovirus (influenza virus) resistance 1, interferon-inducible protein p78 (mouse) (MX1) | 61 |
| hyaluronidase pseudogene 1 (HYALP1) | 15 | C-type lectin superfamily 4, member G (CLEC4G) | 23 | interferon-induced protein 44-like (IFI44L) | 60 |
| corticotropin releasing hormone receptor 1 (CRHR1) | 15 | protein kinase C, beta 1 (PRKCB1), transcript variant 2 | 22 | interferon induced with helicase C domain 1 (IFIH1) | 53 |
| immunoglobulin-like domain containing receptor 1 (ILDR1) | 13 | DEAD (Asp-Glu-Ala-Asp) box polypeptide 25 (DDX25) | 22 | DEAD (Asp-Glu-Ala-Asp) box polypeptide 58 (DDX58) | 52 |
| selenophosphate synthetase 1 (SEPHS1) | 13 | ubiquitin specific protease 51 (USP51) | 21 | interferon induced transmembrane protein 1 (9-27) (IFITM1) | 52 |
| ATP-binding cassette, sub-family A (ABC1), member 6, mRNA (cDNA clone IMAGE:5172925), with apparent retained intron | 12 | keratin 1 (epidermolytic hyperkeratosis) (KRT1) | 16 | N-acetylated alpha-linked acidic dipeptidase-like 1 (NAALADL1) | 47 |
| Kruppel-like factor 8 (KLF8) | 10 | tumor necrosis factor (ligand) superfamily, member 11 (TNFSF11), transcript variant 1 | 16 | PRH2 locus salivary proline-rich protein mRNA (Pr1 allele) | 47 |
| sterile alpha motif domain containing 9-like (SAMD9L) | 10 | C1q and tumor necrosis factor related protein 7 (C1QTNF7) | 16 | clone DNA188146 VCRP9432 (UNQ9432) mRNA | 46 |
| non-classical cadherin XB31alpha1 (XB31alpha) mRNA | 9 | glutathione S-transferase theta 1 (GSTT1) | 14 | peroxisomal proliferator-activated receptor A interacting complex 285 (PRIC285) | 46 |
| guanylate binding protein 1, interferon-inducible, 67kDa (GBP1) | 9 | myosin, light polypeptide 4, alkali; atrial, embryonic (MYL4), transcript variant 2 | 14 | family with sequence similarity 71, member A (FAM71A) | 43 |
| microseminoprotein, beta- (MSMB), transcript variant PSP57 | 9 | thyroid hormone receptor associated protein 1 (THRAP1) | 13 | interleukin 28A (interferon, lambda 2) (IL28A) | 42 |
| zinc finger protein 138 (ZNF138) | 8 | zinc finger protein 179 (ZNF179) | 12 | interferon induced transmembrane protein 1 (9-27) (IFITM1) | 42 |
| pannexin 3 (PANX3) | 8 | Down syndrome critical region gene 8 (DSCR8), transcript variant 1 | 12 | interferon, gamma-inducible protein 16 (IFI16) | 42 |
| transition protein 1 (during histone to protamine replacement) (TNP1) | 8 | activin A receptor, type IC (ACVR1C) | 11 | C21orf22 protein (C21orf22) mRNA, partial sequence | 41 |
| caspase recruitment domain family, member 15 (CARD15) | 8 | interferon, alpha 1 (IFNA1) | 11 | indoleamine-pyrrole 2,3 dioxygenase (INDO) | 40 |
| semenogelin II (SEMG2) | 8 | involucrin (IVL) | 11 | tumor necrosis factor (ligand) superfamily, member 10 (TNFSF10) | 39 |
| CD1D antigen, d polypeptide (CD1D) | 7 | transmembrane channel-like 2 (TMC2) | 10 | apolipoprotein L, 1 (APOL1), transcript variant 1 | 33 |
| amyotrophic lateral sclerosis 2 (juvenile) chromosome region, candidate 16 (ALS2CR16) | 7 | H-rev107-like protein 5 (HRLP5) | 10 | thyroid hormone receptor associated protein 1 (THRAP1) | 32 |
| N-acylsphingosine amidohydrolase (alkaline ceramidase) 3 (ASAH3) | 7 | Jak and microtubule interacting protein 2 (KIAA0555) | 10 | apolipoprotein B (including Ag(x) antigen) (APOB) | 31 |
| sialidase 4 (NEU4) | 7 | protein tyrosine phosphatase, receptor type, N precursor variant protein | 10 | MEGF10 protein (MEGF10) | 30 |
| Cas-Br-M (murine) ecotropic retroviral transforming sequence c (CBLC) | 7 | synaptosomal-associated protein, 91kDa homolog (mouse) (SNAP91) | 10 | transmembrane protease, serine 13 (TMPRSS13) | 30 |
| ankyrin repeat and SOCS box-containing 11 (ASB11), transcript variant 1 | 7 | uroplakin 3A (UPK3A) | 10 | chemokine (C-C motif) ligand 20 (CCL20) | 30 |
| synaptotagmin 1 (SYT14 gene), splice variant 2 | 6 | kinase suppressor of ras 2 (KSR2) | 10 | erythrocyte membrane protein band 41 like 4A (EPB41L4A) | 29 |
| complement component 6 (C6) | 6 | cyclic nucleotide gated channel alpha 1 (CNGA1), mRNA | 9 | ubiquitin-activating enzyme E1-like (UBE1L) | 29 |
| MHC class II HLA-DRA mRNA | 6 | gamma-aminobutyric acid (GABA) receptor, rho 2 (GABRR2) | 9 | ets variant gene 7 (TEL2 oncogene) (ETV7) | 27 |
| RGM domain family, member A (RGMA) | 6 | Krueppel-related zinc finger protein (H-plk) | 9 | guanylate binding protein 3 (GBP3) | 27 |
| intelectin 1 (galactofuranose binding) (ITLN1) | 6 | MEGF10 protein (MEGF10) | 8 | serine (or cysteine) proteinase inhibitor, clade G (C1 inhibitor), member 1, (angioedema, hereditary) (SERPING1) | 27 |
| calpain 3, (p94) (CAPN3), transcript variant 1 | 6 | GTPase, IMAP family member 1 (GIMAP1) | 8 | proteasome (prosome, macropain) subunit, beta type, 9 (large multifunctional protease 2) (PSMB9), transcript variant 1 | 25 |
| A kinase (PRKA) anchor protein 11 (AKAP11), transcript variant 2 | 6 | zinc finger protein 214 (ZNF214) | 8 | potassium channel, subfamily K, member 17 (KCNK17) | 23 |
| prostaglandin I2 (prostacyclin) synthase (PTGIS) | 6 | cytochrome P450, family 4, subfamily F, polypeptide 2 (CYP4F2) | 8 | interferon, alpha 14 (IFNA14) | 23 |
| melanoma antigen family B, 1 (MAGEB1), transcript variant 1 | 6 | deubiquitinating enzyme 3 (DUB3) | 8 | ubiquitin specific protease 51 (USP51) | 22 |
| small proline-rich protein 2A (SPRR2A) | 6 | folate receptor 2 (fetal) (FOLR2) | 8 | zinc finger protein 179 (ZNF179) | 22 |
| junctional adhesion molecule 2 (JAM2) | 6 | complement component 1, q subcomponent, gamma polypeptide (C1QG) | 8 | chemokine (C-C motif) receptor-like 1 (CCRL1), transcript variant 2 | 22 |
| catenin (cadherin-associated protein), alpha 2 (CTNNA2) | 6 | dickkopf-like 1 (soggy) (DKKL1) | 8 | cytochrome P450, family 4, subfamily F, polypeptide 2 (CYP4F2) | 21 |
| leucine rich repeat containing 2 (LRRC2) | 6 | CD5 antigen-like (scavenger receptor cysteine rich family) (CD5L) | 8 | tumor necrosis factor, alpha-induced protein 6 (TNFAIP6) | 21 |
| TNNI3 interacting kinase (TNNI3K) | 6 | Spi-C transcription factor (Spi-1/PU1 related) (SPIC) | 8 | sterile alpha motif domain containing 9 (SAMD9) | 21 |
| synleurin (SLRN) | 6 | transcription elongation regulator 1-like (TCERG1L) | 7 | interferon regulatory factor 1 (IRF1) | 20 |
| mitogen-activated protein kinase 10 (MAPK10), transcript variant 1 | 5 | sulfotransferase family, cytosolic, 1C, member 3 (SULT1C3) | 7 | coagulation factor V (proaccelerin, labile factor) (F5) | 20 |
| zinc finger, CCHC domain containing 13 (ZCCHC13) | 5 | fibronectin type III and ankyrin repeat domains 1 (FANK1) | 7 | cold autoinflammatory syndrome 1 (CIAS1), transcript variant 1 | 19 |
| ets homologous factor (EHF) | 5 | keratin 6E (KRT6E) | 7 | uroplakin 3A (UPK3A) | 19 |
| tumor protein p73 (TP73) | 5 | LIM and senescent cell antigen-like domains 2 (LIMS2) | 7 | apolipoprotein L, 3 (APOL3), transcript variant alpha/a | 19 |
| testis transcript Y 11 (TTY11) mRNA | 5 | N-acetyltransferase 8 (camello like) (NAT8) | 7 | protein phosphatase 1K (PP2C domain containing) (PPM1K) | 19 |
| cerebellin 4 precursor (CBLN4) | 5 | prostaglandin I2 (prostacyclin) synthase (PTGIS) | 7 | CD28 antigen (Tp44) (CD28) | 19 |
| clusterin-like 1 (retinal) (CLUL1), transcript variant 2 | 5 | thyroid hormone receptor, beta (erythroblastic leukemia viral (v-erb-a) oncogene homolog 2, avian) (THRB) | 7 | SP110 nuclear body protein (SP110), transcript variant a | 18 |
| sine oculis homeobox homolog 6 (Drosophila) (SIX6) | 5 | neurexophilin 1 (NXPH1) | 7 | hect domain and RLD 5 (HERC5) | 18 |
| START domain containing 4, sterol regulated (STARD4) | 5 | reprimo, TP53 dependant G2 arrest mediator candidate (RPRM) | 6 | transporter 1, ATP-binding cassette, sub-family B (MDR/TAP) (TAP1) | 18 |
| nuclear receptor subfamily 5, group A, member 1 (NR5A1) | 5 | Fc fragment of IgA, receptor for (FCAR), transcript variant 6 | 6 | epithelial stromal interaction 1 (breast) (EPSTI1) | 18 |
| taste receptor, type 2, member 8 (TAS2R8) | 5 | NADH dehydrogenase (ubiquinone) 1 beta subcomplex, 8, 19kDa (NDUFB8) | 6 | H-rev107-like protein 5 (HRLP5) | 18 |
| glycerophosphodiester phosphodiesterase domain containing 4 (GDPD4) | 5 | mucin 15 (MUC15) | 6 | myxovirus (influenza virus) resistance 2 (mouse) (MX2) | 18 |
| amyloid P component, serum (APCS) | 5 | growth hormone 1 (GH1), transcript variant 1 | 6 | B-factor, properdin (BF) | 18 |
| programmed cell death 1 ligand 2 (PDCD1LG2) | 5 | hypothetical protein MGC46496 (MGC46496) | 6 | normal mucosa of esophagus specific 1 (NMES1), transcript variant 1 | 18 |
| chemokine (C-X-C motif) ligand 6 (granulocyte chemotactic protein 2) (CXCL6) | 5 | granzyme A (granzyme 1, cytotoxic T-lymphocyte-associated serine esterase 3) (GZMA) | 6 | lysosomal-associated membrane protein 3 (LAMP3) | 18 |
| CUB and zona pellucida-like domains 1 (CUZD1) | 5 | potassium channel tetramerisation domain containing 4 (KCTD4) | 6 | endothelial cell growth factor 1 (platelet-derived) (ECGF1) | 17 |
| N-acetylated alpha-linked acidic dipeptidase 2 (NAALADL2) | 5 | Friend leukemia virus integration 1 (FLI1) | 6 | apolipoprotein L, 2 (APOL2), transcript variant alpha | 17 |
| hypothetical protein FLJ35880 (FLJ35880) | 5 | cell adhesion molecule with homology to L1CAM (close homolog of L1) (CHL1) | 6 | ATP-binding cassette, sub-family B (MDR/TAP), member 1 (ABCB1) | 16 |
| ATP-binding cassette, sub-family D (ALD), member 2 (ABCD2) | 4 | pancreatic lipase (PNLIP) | 6 | 2'-5'-oligoadenylate synthetase 2, 69/71kDa (OAS2), transcript variant 2 | 16 |
| EGF-like-domain, multiple 6 (EGFL6) | 4 | F1(P4)1 CFS leukocyte Homo sapiens cDNA, mRNA sequence | 6 | sterile alpha motif domain containing 9-like (SAMD9L) | 16 |
| leucine rich repeat containing 2 (LRRC2) | 4 | RNA-binding protein BRUNOL3 (BRUNOL3) mRNA | 6 | fibronectin type III domain containing 6 (FNDC6) | 16 |
| troponin I, skeletal, fast (TNNI2) | 4 | iroquois homeobox protein 1 (IRX1) | 6 | Krueppel-related zinc finger protein (H-plk) | 16 |
| LS1/EVB Ig V(kappa), autoantibody with RBC specifity | 4 | glial fibrillary acidic protein (GFAP) | 5 | phospholipid scramblase 1 (PLSCR1) | 15 |
| reticulon 1 (RTN1), transcript variant 1 | 4 | apolipoprotein A-V (APOA5) | 5 | SAM domain and HD domain 1 (SAMHD1) | 15 |
| fatty acid binding protein 2, intestinal (FABP2) | 4 | Cas-Br-M (murine) ecotropic retroviral transforming sequence c (CBLC) | 5 | C-type lectin superfamily 4, member G (CLEC4G) | 15 |
| serine (or cysteine) proteinase inhibitor, clade B (ovalbumin), member 11 (SERPINB11) | 4 | absent in melanoma 2 (AIM2) | 5 | glutathione S-transferase theta 1 (GSTT1) | 15 |
| protein serine kinase H2 (PSKH2) | 4 | angiotensin I converting enzyme (peptidyl-dipeptidase A) 2 (ACE2) | 5 | tumor necrosis factor (ligand) superfamily, member 13b (TNFSF13B) | 14 |
| sodium channel, voltage-gated, type II, alpha 2 (SCN2A2) | 4 | multiple C2-domains with two transmembrane regions 2, mRNA (cDNA clone IMAGE:5276211), containing frame-shift s | 5 | interferon induced transmembrane protein 3 (1-8U) (IFITM3) | 14 |
| synaptotagmin VII mRNA | 4 | matrix metalloproteinase 27 (MMP27) | 5 | transferrin (TF) | 14 |
| 5-hydroxytryptamine (serotonin) receptor 2A (HTR2A) | 4 | glutathione peroxidase 5 (epididymal androgen-related protein) (GPX5), transcript variant 2 | 5 | ubiquitin specific protease 9, Y-linked (fat facets-like, Drosophila) (USP9Y) | 14 |
| membrane-spanning 4-domains, subfamily A, member 6A (MS4A6A), transcript variant 2 | 4 | neurotensin receptor 1 (high affinity) (NTSR1) | 5 | transcription elongation regulator 1-like (TCERG1L) | 14 |
| astrotactin 2 (ASTN2), transcript variant 1 | 4 | CD28 antigen (Tp44) (CD28) | 5 | retinoic acid receptor responder (tazarotene induced) 3 (RARRES3) | 14 |
| recombination activating gene 2 (RAG2) | 4 | leucine-rich repeat-containing G protein-coupled receptor 5 (LGR5) | 5 | Z-DNA binding protein 1 (ZBP1) | 14 |
| EF-hand domain (C-terminal) containing 2 (EFHC2) | 4 | jerky homolog-like (mouse) (JRKL) | 5 | potassium voltage-gated channel, subfamily G, member 3 (KCNG3), transcript variant 1 | 14 |
| NACHT, leucine rich repeat and PYD containing 9 (NALP9) | 4 | G protein-coupled receptor 51 (GPR51) | 5 | poly (ADP-ribose) polymerase family, member 9 (PARP9) | 14 |
| tryptase alpha/beta 1 (TPSAB1) | 4 | adhesion molecule, interacts with CXADR antigen 1 (AMICA1) | 5 | hypothetical protein FLJ25660 (FLJ25660) | 14 |
| synaptojanin 1 (SYNJ1), transcript variant 2 | 4 | plexin A4, B (PLXNA4B) | 5 | programmed cell death 1 ligand 2 (PDCD1LG2) | 13 |
| zinc finger protein 396 (ZNF396) | 4 | CD48 antigen (B-cell membrane protein) (CD48) | 5 | myosin binding protein C, cardiac (MYBPC3) | 13 |
| HLH-PAS transcription factor NXF (NXF) | 4 | placenta-specific 4 (PLAC4) | 5 | interferon-induced protein 35 (IFI35) | 13 |
| glycoprotein 2 (zymogen granule membrane) (GP2), transcript variant 2 | 4 | cysteine-rich secretory protein 1 (CRISP1), transcript variant 1 | 5 | poly (ADP-ribose) polymerase family, member 10 (PARP10) | 13 |
| calmodulin-like 5 (CALML5) | 4 | histone 1, H4g (HIST1H4G) | 5 | transporter 2, ATP-binding cassette, sub-family B (MDR/TAP) (TAP2), transcript variant 2 | 12 |
| DEAD (Asp-Glu-Ala-Asp) box polypeptide 4 (DDX4), transcript variant 2 | 4 | oxytocin, prepro- (neurophysin I) (OXT) | 5 | oxytocin, prepro- (neurophysin I) (OXT) | 12 |
| melanoma antigen family A, 8 (MAGEA8) | 4 | Cas-Br-M (murine) ecotropic retroviral transforming sequence c (CBLC) | 5 | ubiquitin-conjugating enzyme E2L 6 (UBE2L6), transcript variant 2 | 12 |
| resistin like beta (RETNLB) | 4 | EPH receptor B6 (EPHB6) | 5 | keratin 1 (epidermolytic hyperkeratosis) (KRT1) | 12 |
| taste receptor, type 2, member 10 (TAS2R10) | 4 | histidine decarboxylase (HDC) | 5 | apolipoprotein L, 6 (APOL6) | 11 |
| chloride channel, calcium activated, family member 2 (CLCA2) | 4 | inactivation escape 1 (INE1) | 5 | cytochrome P450, family 2, subfamily J, polypeptide 2 (CYP2J2) | 11 |
| guanylate cyclase 2C (heat stable enterotoxin receptor) (GUCY2C) | 4 | NACHT, leucine rich repeat and PYD containing 10 (NALP10) | 5 | similar to KIAA1680 protein (MGC48628) | 11 |
| bactericidal/permeability-increasing protein-like 3 (BPIL3) | 4 | chemokine (C-C motif) ligand 8 (CCL8) | 5 | poly (ADP-ribose) polymerase family, member 14 (PARP14) | 11 |
| protein tyrosine phosphatase, non-receptor type 22 (lymphoid) (PTPN22), transcript variant 2 | 4 | neuroligin 1 (NLGN1) | 5 | leucine aminopeptidase 3 (LAP3) | 11 |
| WD repeat domain 78 (WDR78), transcript variant 2 | 4 | delta-like 1 (Drosophila) (DLL1) | 4 | zinc finger and BTB domain containing 37 (ZBTB37) | 11 |
| RAS guanyl releasing protein 1 (calcium and DAG-regulated) (RASGRP1) | 4 | zinc finger protein 626 (ZNF626) | 4 | guanine nucleotide binding protein (G protein), gamma transducing activity polypeptide 2 (GNGT2) | 11 |
| glutamate receptor, metabotropic 3 (GRM3) | 4 | dehydrogenase/reductase (SDR family) member 6 (DHRS6) | 4 | activin A receptor, type IC (ACVR1C) | 10 |
| interleukin 1 family, member 10 (theta) (IL1F10), transcript variant 1 | 4 | TBC1 domain family, member 10C (TBC1D10C) | 4 | interferon-stimulated transcription factor 3, gamma 48kDa (ISGF3G) | 10 |
| zinc finger protein 100, mRNA (cDNA clone IMAGE:3344728), with apparent retained intron | 4 | histone 1, H2bc (HIST1H2BC) | 4 | hypothetical protein FLJ37538 (FLJ37538) | 10 |
| glial fibrillary acidic protein (GFAP) | 4 | CD300 antigen like family member G (CD300LG) | 4 | tumor necrosis factor (ligand) superfamily, member 11 (TNFSF11), transcript variant 1 | 10 |
| fructose-1,6-bisphosphatase 1 (FBP1) | 4 | Rho guanine nucleotide exchange factor (GEF) 7 (ARHGEF7), transcript variant 2 | 4 | tumor protein p53 inducible protein 5 (TP53I5) | 10 |
| butyrophilin-like 3 (BTNL3), transcript variant 2 | 4 | zinc finger protein 433 (ZNF433) | 4 | baculoviral IAP repeat-containing 3 (BIRC3), transcript variant 1 | 10 |
| interleukin 8 receptor, alpha (IL8RA) | 4 | flavin containing monooxygenase 3 (FMO3), transcript variant 1 | 4 | pleckstrin homology domain containing, family A (phosphoinositide binding specific) member 4 (PLEKHA4) | 9 |
| high affinity choline transporter (CHT1 gene) | 4 | gamma-aminobutyric acid (GABA) A receptor, pi (GABRP) | 4 | cytochrome P450, family 2, subfamily C, polypeptide 8 (CYP2C8), transcript variant Hp1-2 | 9 |
| serine (or cysteine) proteinase inhibitor, clade G (C1 inhibitor), member 1, (angioedema, hereditary) (SERPING1) | 4 | chemokine (C-C motif) receptor 5 (CCR5) | 4 | involucrin (IVL) | 9 |
| differentially expressed in FDCP 6 homolog (mouse) (DEF6) | 4 | sperm associated antigen 11 (SPAG11), transcript variant E | 4 | hematopoietic SH2 domain containing (HSH2D) | 9 |
| SH3 and multiple ankyrin repeat domains 3, mRNA (cDNA clone IMAGE:5572993) | 4 | transferrin (TF) | 4 | cathepsin S (CTSS) mRNA | 9 |
| lysozyme-like 4 (LYZL4) | 3 | phytanoyl-CoA dioxygenase domain containing 1 (PHYHD1) | 4 | interleukin 15 receptor, alpha (IL15RA), transcript variant 1 | 9 |
| MAGE-like 2 (MAGEL2) | 3 | neuritin 1 (NRN1) | 4 | ankyrin repeat and SOCS box-containing 17 (ASB17) | 9 |
| cylicin, basic protein of sperm head cytoskeleton 2 (CYLC2) | 3 | ring finger protein 148 (RNF148) | 4 | nuclear antigen Sp100 (SP100) | 9 |
| Fc fragment of IgG, low affinity IIb, receptor (CD32) (FCGR2B), transcript variant 4 | 3 | steroidogenic acute regulator (STAR), nuclear gene encoding mitochondrial protein, transcript variant 2 | 4 | fibroblast activation protein, alpha (FAP) | 9 |
| leukocyte-derived arginine aminopeptidase (LRAP) | 3 | type-1 protein phosphatase inhibitor | 4 | leucine-rich repeats and calponin homology (CH) domain containing 2 (LRCH2) | 9 |
| S100 calcium binding protein, beta (neural) (S100B) | 3 | ATPase, Na+/K+ transporting, alpha 4 polypeptide (ATP1A4), transcript variant 1 | 4 | proteasome (prosome, macropain) subunit, beta type, 10 (PSMB10) | 9 |
| toll-like receptor 10 (TLR10), transcript variant 1 | 3 | semenogelin II (SEMG2) | 4 | delta-like 1 (Drosophila) (DLL1) | 9 |
| zinc finger protein 418 (ZNF418) | 3 | beta-carotene dioxygenase 2 (BCDO2) | 4 | interleukin 18 binding protein (IL18BP), transcript variant C | 9 |
| HIRA interacting protein 3 (HIRIP3) | 3 | apolipoprotein B48 receptor (APOB48R) | 4 | proteasome (prosome, macropain) subunit, beta type, 8 (large multifunctional protease 7) (PSMB8), transcript variant 2 | 9 |
| polycystic kidney and hepatic disease 1 (PKHD1) mRNA | 3 | integrin, alpha 2b (platelet glycoprotein IIb of IIb/IIIa complex, antigen CD41B) (ITGA2B) | 4 | chemokine (C-X-C motif) ligand 2 (CXCL2) | 9 |
| glycoprotein hormones, alpha polypeptide (CGA) | 3 | ST6 (alpha-N-acetyl-neuraminyl-2,3-beta-galactosyl-1,3)-N-acetylgalactosaminide alpha-2,6-sialyltransferase 1 (ST6GALNAC1) | 4 | radical S-adenosyl methionine domain containing 2 (RSAD2) | 9 |
| a disintegrin and metalloproteinase domain 22 (ADAM22), transcript variant 4 | 3 | protease, serine, 8 (prostasin) (PRSS8) | 4 | interleukin-1 precursor (pre IL-1) | 8 |
| fibroblast growth factor 19 (FGF19) | 3 | T CELLS (JURKAT CELL LINE) COT 10-NORMALIZED cDNA clone CS0DJ014YH16 3-PRIME | 4 | major histocompatibility complex, class I, F (HLA-F) | 8 |
| cytochrome P450, family 3, subfamily A, polypeptide 5 (CYP3A5) | 3 | bone morphogenetic protein 3 (osteogenic) (BMP3) | 4 | transmembrane channel-like 2 (TMC2) | 8 |
| SH2 domain containing 1B (SH2D1B) | 3 | cysteine-rich secretory protein 3 (CRISP3) | 4 | HLA class I histocompatibility antigen, E alpha chain precursor variant protein | 8 |
| CD36 antigen (collagen type I receptor, thrombospondin receptor) (CD36), transcript variant 2 | 3 | hypothetical protein FLJ36046 (FLJ36046) | 4 | B-box and SPRY domain containing (BSPRY) | 8 |
| chemokine-like factor superfamily 2 (CKLFSF2) | 3 | PDZ and LIM domain 3 (PDLIM3) | 4 | GTPase, IMAP family member 2 (GIMAP2) | 8 |
| opioid binding protein/cell adhesion molecule-like (OPCML), transcript variant 2 | 3 | calcyphosine 2 (CAPS2) | 4 | G protein-coupled receptor 151 (GPR151) | 8 |
| neurofilament, heavy polypeptide 200kDa (NEFH) | 3 | IQ motif containing with AAA domain (IQCA) | 4 | matrix metalloproteinase 27 (MMP27) | 8 |
| phytanoyl-CoA dioxygenase domain containing 1 (PHYHD1) | 3 | chemokine (C-C motif) ligand 1 (CCL1) | 4 | TAP binding protein-like (TAPBPL) | 8 |
| high density lipoprotein-binding protein (LOC338328) | 3 | guanine nucleotide binding protein (G protein), alpha transducing activity polypeptide 1 (GNAT1), transcript variant 1 | 4 | tripartite motif-containing 21 (TRIM21) | 8 |
| P antigen family, member 3 (prostate associated) (PAGE3) | 3 | myeloid/lymphoid or mixed-lineage leukemia (trithorax homolog, Drosophila) (MLL) | 4 | Down syndrome critical region gene 8 (DSCR8), transcript variant 1 | 8 |
| glutamate receptor, metabotropic 4 (GRM4) | 3 | ADP-ribosylation factor 7 (ARF7) | 4 | inter-alpha (globulin) inhibitor H3 (ITIH3) | 8 |
| protease, serine, 2 (trypsin 2) (PRSS2), transcript variant 1 | 3 | 82-kD FMRP Interacting Protein (182-FIP) | 4 | angiotensin I converting enzyme (peptidyl-dipeptidase A) 2 (ACE2) | 8 |
| keratin 5 (epidermolysis bullosa simplex, Dowling-Meara/Kobner/Weber-Cockayne types) (KRT5) | 3 | X-prolyl aminopeptidase (aminopeptidase P) 2, membrane-bound (XPNPEP2) | 4 | hornerin | 8 |
| dapper, antagonist of beta-catenin, homolog 1 (Xenopus laevis) (DACT1) | 3 | TAR DNA binding protein (TARDBP) | 4 | complement component 4A (C4A) | 8 |
| trans-golgi network protein 2 (TGOLN2) | 3 | kallikrein 5 (KLK5) | 4 | signal transducer and activator of transcription 2, 113kDa (STAT2) | 7 |
| potassium voltage-gated channel, Shab-related subfamily, member 2 (KCNB2) | 3 | Kruppel-like factor 1 (erythroid) (KLF1) | 4 | 2',5'-oligoadenylate synthetase 1, 40/46kDa (OAS1), transcript variant E18 | 7 |
| synaptotagmin 16 (SYT16 gene), splice variant 1 | 3 | orosomucoid 1 (ORM1) | 4 | 5'-nucleotidase, cytosolic III (NT5C3), transcript variant 3 | 7 |
| golgi SNAP receptor complex member 2 (GOSR2), transcript variant A | 3 | chondroitin sulfate synthase 3 (CSS3) | 4 | 5-hydroxytryptamine (serotonin) receptor 2A (HTR2A) | 7 |
| signaling lymphocytic activation molecule family member 1 (SLAMF1) | 3 | PDZ and LIM domain 4 (PDLIM4) | 4 | phospholipase C, beta 1 (phosphoinositide-specific) (PLCB1), transcript variant 1 | 7 |
| mRNA; cDNA DKFZp686L18111 (from clone DKFZp686L18111) | 3 | thyroid peroxidase (TPO), transcript variant 1 | 4 | polyribonucleotide nucleotidyltransferase 1 (PNPT1) | 7 |
| FXYD domain containing ion transport regulator 7 (FXYD7) | 3 | olfactory receptor, family 2, subfamily M, member 4 (OR2M4) | 4 | protocadherin beta 4 (PCDHB4) | 7 |
| three prime repair exonuclease 2 (TREX2) | 3 | actin-related protein T1 (RP1-203C21) | 4 | complement component 1, r subcomponent (C1R) | 7 |
| kinesin family member 25 (KIF25), transcript variant 2 | 3 | T-cell library (Sugita Y) cDNA clone B02467-025, mRNA sequence | 4 | H2B histone family, member W, testis-specific (H2BFWT) | 7 |
| selenophosphate synthetase 1 (SEPHS1) | 3 | keratin associated protein 3-3 (KRTAP3-3) | 4 | thyroid hormone receptor, beta (erythroblastic leukemia viral (v-erb-a) oncogene homolog 2, avian) (THRB) | 7 |
| keratin 12 (Meesmann corneal dystrophy) (KRT12) | 3 | cytochrome P450, family 2, subfamily U, polypeptide 1, mRNA (cDNA clone IMAGE:4706746) | 4 | tryptophanyl-tRNA synthetase (WARS), transcript variant 1 | 7 |
| G protein-coupled receptor 23 (GPR23) | 3 | similar to RIKEN cDNA 2010001P08 gene (LOC345062), mRNA | 4 | interleukin 8 (IL8) | 7 |
| zinc finger protein 267 (ZNF267), transcript variant 498723 | 3 | pygopus homolog 2 (Drosophila) (PYGO2) | 4 | interferon stimulated exonuclease gene 20kDa (ISG20) | 7 |
| Krueppel-related zinc finger protein (H-plk) | 3 | interleukin 26 (IL26) | 3 | otoraplin (OTOR) | 7 |
| interferon, alpha 14 (IFNA14) | 3 | leucine-rich repeats and calponin homology (CH) domain containing 2 (LRCH2) | 3 | Rho guanine nucleotide exchange factor (GEF) 7 (ARHGEF7), transcript variant 2 | 7 |
| GTPase, IMAP family member 1 (GIMAP1) | 3 | serine (or cysteine) proteinase inhibitor, clade A (alpha-1 antiproteinase, antitrypsin), member 4 (SERPINA4) | 3 | olfactory receptor, family 2, subfamily M, member 4 (OR2M4) | 7 |
| neuritin 1 (NRN1) | 3 | serine protease inhibitor-like, with Kunitz and WAP domains 1 (eppin) (SPINLW1), transcript variant 1 | 3 | C1q and tumor necrosis factor related protein 7 (C1QTNF7) | 7 |
| RIM binding protein 2 (KIAA0318) | 3 | arginyltransferase 1 (ATE1), transcript variant 1 | 3 | nuclear antigen Sp100 (SP100) | 7 |
| membrane metallo-endopeptidase (neutral endopeptidase, enkephalinase, CALLA, CD10) (MME), transcript variant 2b | 3 | cadherin 7, type 2 (CDH7), transcript variant b | 3 | phytanoyl-CoA dioxygenase domain containing 1 (PHYHD1) | 7 |
| allograft inflammatory factor 1 (AIF1), transcript variant 1 | 3 | interleukin 7 receptor (IL7R) | 3 | iroquois homeobox protein 1 (IRX1) | 7 |
| tyrosinase (oculocutaneous albinism IA) (TYR) | 3 | inducible T-cell co-stimulator ligand (ICOSLG) | 3 | growth arrest-specific 7 (GAS7), transcript variant b | 7 |
| frizzled homolog 3 (Drosophila) (FZD3) | 3 | cerebellin 4 precursor (CBLN4) | 3 | interferon regulatory factor 1 variant protein | 7 |
| megakaryocyte-associated tyrosine kinase (MATK), transcript variant 1 | 3 | junctional adhesion molecule 2 (JAM2) | 3 | CD5 antigen-like (scavenger receptor cysteine rich family) (CD5L) | 7 |
| MCF2 cell line derived transforming sequence (MCF2) | 3 | glycoprotein hormones, alpha polypeptide (CGA) | 3 | ubiquitin specific protease 18 (USP18) | 7 |
| 602855753F1 NIH_MGC_10 cDNA clone IMAGE:4997101 5' | 3 | DNA-damage inducible protein 1 (DDI1) | 3 | G protein-coupled receptor 43 (GPR43) | 7 |
| DEP domain containing 1 (DEPDC1) | 3 | protocadherin 12 (PCDH12) | 3 | interferon, alpha-inducible protein (clone IFI-6-16) (G1P3), transcript variant 3 | 7 |
| zymogen granule protein 16 (ZG16) | 3 | phosphatidylinositol-4-phosphate 5-kinase, type I, beta (PIP5K1B) | 3 | colony stimulating factor 1 (macrophage) (CSF1), transcript variant 1 | 7 |
| zinc finger, matrin type 1 (ZMAT1), transcript variant 2 | 3 | myogenic factor 5 (MYF5) | 3 | lectin, galactoside-binding, soluble, 3 binding protein (LGALS3BP) | 7 |
| tumor necrosis factor (ligand) superfamily, member 14 (TNFSF14), transcript variant 1 | 3 | glutamate/aspartate transporter II mRNA | 3 | leukocyte-derived arginine aminopeptidase (LRAP) | 7 |
| histone 1, H2ad (HIST1H2AD) | 3 | serine (or cysteine) proteinase inhibitor, clade I (pancpin), member 2 (SERPINI2), transcript variant 2 | 3 | steroidogenic acute regulator (STAR), nuclear gene encoding mitochondrial protein, transcript variant 2 | 7 |
| naked cuticle homolog 1 (Drosophila) (NKD1) | 3 | histone 1, H2aa (HIST1H2AA) | 3 | tachykinin 3 (neuromedin K, neurokinin beta) (TAC3) | 7 |
| zinc finger protein 483 (ZNF483), transcript variant 1 | 3 | protocadherin 1 (cadherin-like 1) (PCDH1), transcript variant 2 | 3 | ubiquitin specific protease 16 (USP16), transcript variant 2 | 7 |
| proopiomelanocortin (adrenocorticotropin/ beta-lipotropin/ alpha-melanocyte stimulating hormone/ beta-melanocyte stimulating hormone/ beta-endorphin) (POMC) | 3 | DIRAS family, GTP-binding RAS-like 2 (DIRAS2) | 3 | activating transcription factor 3 (ATF3), transcript variant 2, mRNA | 7 |
| interphotoreceptor matrix proteoglycan 1 (IMPG1) | 3 | T-cell leukemia, homeobox 2, mRNA (cDNA clone IMAGE:4135413) | 3 | neurexophilin 1 (NXPH1) | 7 |
| protocadherin beta 3 (PCDHB3) | 3 | colony stimulating factor 2 (granulocyte-macrophage) (CSF2) | 3 | CD68 antigen (CD68) | 6 |
| potassium voltage-gated channel, KQT-like subfamily, member 2 (KCNQ2), transcript variant 3 | 3 | NACHT, leucine rich repeat and PYD containing 11 (NALP11) | 3 | zinc finger protein 14 (KOX 6) (ZNF14) | 6 |
| zinc finger protein 521 (ZNF521) | 3 | calpain 11 (CAPN11) | 3 | tumor necrosis factor, alpha-induced protein 3 (TNFAIP3) | 6 |
| purinergic receptor P2X, ligand-gated ion channel, 7 (P2RX7), transcript variant 1 | 3 | matrix metalloproteinase 8 (neutrophil collagenase) (MMP8) | 3 | guanine nucleotide binding protein (G protein), alpha transducing activity polypeptide 1 (GNAT1), transcript variant 1 | 6 |
| T-box 15 (TBX15) | 3 | defensin, beta 104A (DEFB104A) | 3 | jerky homolog-like (mouse) (JRKL) | 6 |
| calcium binding and coiled-coil domain 1 (CALCOCO1) | 3 | Fc fragment of IgG, low affinity IIIb, receptor (CD16b) (FCGR3B) | 3 | synaptosomal-associated protein, 91kDa homolog (mouse) (SNAP91) | 6 |
| nuclear RNA export factor 3 (NXF3) | 3 | inter-alpha (globulin) inhibitor H5 (ITIH5), transcript variant 2 | 3 | TAR DNA binding protein (TARDBP) | 6 |
| crystallin, gamma D (CRYGD) | 3 | zinc finger protein 598 (ZNF598) | 3 | phospholipase A1 member A (PLA1A) | 6 |
| tudor domain containing 5 (TDRD5) | 3 | zinc finger protein 85 (HPF4, HTF1) (ZNF85) | 3 | cell adhesion molecule with homology to L1CAM (close homolog of L1) (CHL1) | 6 |
| 5-hydroxytryptamine (serotonin) receptor 1E (HTR1E) | 3 | 5-hydroxytryptamine (serotonin) receptor 2A (HTR2A) | 3 | ubiquitin D (UBD) | 6 |
| regulator of G-protein signalling 18 (RGS18) | 3 | gamma-aminobutyric acid (GABA) A receptor, alpha 2 (GABRA2) | 3 | chemokine (C-X-C motif) ligand 11 (CXCL11) | 6 |
| TBC1 (tre-2/USP6, BUB2, cdc16) domain family, member 1, mRNA (cDNA clone IMAGE:5211948), with apparent retained intron | 3 | kinase non-catalytic C-lobe domain (KIND) containing 1 (KNDC1), transcript variant 1 | 3 | carcinoembryonic antigen-related cell adhesion molecule 1 (biliary glycoprotein) (CEACAM1), transcript variant 1 | 6 |
| CD4 antigen (p55) (CD4) | 3 | Neural cell adhesion molecule 1, 120 kDa isoform precursor variant protein | 3 | deubiquitinating enzyme 3 (DUB3) | 6 |
| syntaxin 3 mRNA | 3 | Rhesus blood group-associated glycoprotein (RHAG) | 3 | teratocarcinoma-derived growth factor 1 (TDGF1) | 6 |
| ring finger protein 186 (RNF186) | 3 | multiple coiled-coil GABABR1-binding protein (MARLIN1) | 3 | hect domain and RLD 6 (HERC6), transcript variant 1 | 6 |
| histone 1, H2ae (HIST1H2AE) | 3 | claudin 8 (CLDN8) | 3 | protein kinase D2 (PRKD2) | 6 |
| baculoviral IAP repeat-containing 1 (BIRC1) | 3 | spermatogenesis associated 9 (SPATA9), transcript variant 1 | 3 | fibronectin type III and ankyrin repeat domains 1 (FANK1) | 6 |
| interferon, alpha 5 (IFNA5) | 3 | zinc finger protein 20 (KOX 13) (ZNF20) | 3 | clone 18 (HL-18), dynein heavy chain (Dnahc14) mRNA | 6 |
| Rhesus blood group, D antigen (RHD), transcript variant 1 | 3 | EPH receptor A3 (EPHA3), transcript variant 1 | 3 | SCA8 mRNA, repeat region | 6 |
| crystallin, beta A1 (CRYBA1) | 3 | tripartite motif-containing 2 (TRIM2) | 3 | stannin (SNN) | 6 |
| small proline-rich protein 2A (SPRR2A) | 3 | Kruppel-like factor 8 (KLF8) | 3 | PHD finger protein 11 (PHF11) | 6 |
| cat eye syndrome chromosome region, candidate 1 (CECR1), transcript variant 1 | 3 | histone 2, H2ab (HIST2H2AB) | 3 | beta-2-microglobulin (B2M) | 6 |
| distal-less homeo box 2 (DLX2) | 3 | fascin homolog 3, actin-bundling protein, testicular (Strongylocentrotus purpuratus) (FSCN3) | 3 | family with sequence similarity 46, member A (FAM46A) | 6 |
| dendritic cell-associated lectin-1 (DCAL1) | 3 | zinc finger, SWIM-type containing 2 (ZSWIM2) | 3 | complement component 3 (C3) | 6 |
| developmental pluripotency associated 3 (DPPA3) | 3 | DC-STAMP domain containing 1 (DCST1) | 3 | deiodinase, iodothyronine, type I (DIO1), transcript variant 2 | 6 |
| pleiomorphic adenoma gene 1 (PLAG1) | 3 | zinc finger protein 253 (ZNF253) | 3 | myotubularin related protein 7 mRNA | 6 |
| killer cell immunoglobulin-like receptor, two domains, long cytoplasmic tail, 4 (KIR2DL4) | 3 | Rap guanine nucleotide exchange factor (GEF) 5 (RAPGEF5) | 3 | signal transducer and activator of transcription 1, 91kDa (STAT1), transcript variant alpha | 6 |
| a disintegrin and metalloproteinase domain 32 (ADAM32) | 3 | guanylate cyclase 1, soluble, alpha 3 (GUCY1A3) | 3 | U2AF homology motif (UHM) kinase 1 (UHMK1) | 6 |
| cytochrome P450, family 39, subfamily A, polypeptide 1 (CYP39A1) | 3 | FLJ46419 protein (FLJ46419) | 3 | mixed lineage kinase domain-like (MLKL) | 6 |
| cullin 3 (CUL3) | 3 | dickkopf homolog 2 (Xenopus laevis) (DKK2) | 3 | B-lymphoma and BAL associated protein (BBAP) mRNA | 5 |
| RNA binding motif protein 11 (RBM11) | 3 | glutamate decarboxylase 2 (pancreatic islets and brain, 65kDa) (GAD2) | 3 | crystallin, gamma B (CRYGB) | 5 |
| retinol binding protein 3, interstitial (RBP3) | 3 | heparan sulfate 6-O-sulfotransferase 3 (HS6ST3) | 3 | elongation of very long chain fatty acids (FEN1/Elo2, SUR4/Elo3, yeast)-like 3 (ELOVL3) | 5 |
| kallikrein 5 (KLK5) | 3 | cullin 3 (CUL3) | 3 | caspase 4, apoptosis-related cysteine protease (CASP4), transcript variant alpha | 5 |
| Kallmann syndrome 1 sequence (KAL1) | 3 | mel transforming oncogene-like 1 (MELL1) | 3 | calcyphosine 2 (CAPS2) | 5 |
| inositol hexaphosphate kinase 3 (IHPK3) | 3 | transmembrane protein 46 (TMEM46) | 3 | interleukin 7 (IL7) | 5 |
| chondrolectin (CHODL) | 3 | TBC1 (tre-2/USP6, BUB2, cdc16) domain family, member 1, mRNA (cDNA clone IMAGE:5211948), with apparent retained intron | 3 | sulfotransferase family, cytosolic, 1C, member 3 (SULT1C3) | 5 |
| cyclic nucleotide gated channel alpha 2 (CNGA2) | 3 | selenoprotein V (SELV) | 3 | protein kinase C, beta 1 (PRKCB1), transcript variant 2 | 5 |
| purinergic receptor P2X, ligand-gated ion channel, 3 (P2RX3) | 3 | ring finger protein 150 (RNF150) | 3 | junctional adhesion molecule 2 (JAM2) | 5 |
| glutamate receptor, ionotropic, N-methyl D-aspartate 2D (GRIN2D) | 3 | serine (or cysteine) proteinase inhibitor, clade B (ovalbumin), member 2 (SERPINB2) | 3 | HLA-G histocompatibility antigen, class I, G, mRNA (cDNA clone IMAGE:4694038) | 5 |
| fibroblast growth factor-5 (FGF-5) mRNA | 3 | calpain 5 (CAPN5) | 3 | myeloid differentiation primary response gene (88) (MYD88) | 5 |
| cystatin-like 1 (CSTL1) | 3 | proenkephalin (PENK) | 3 | keratin 6E (KRT6E) | 5 |
| SH3-domain GRB2-like 2 (SH3GL2) | 3 | intelectin 1 (galactofuranose binding) (ITLN1) | 3 | hepcidin antimicrobial peptide (HAMP) | 5 |
| RAX-like homeobox (RAXLX) | 3 | CD3D antigen, delta polypeptide (TiT3 complex) (CD3D) | 3 | sialic acid binding Ig-like lectin 12 (SIGLEC12), transcript variant 2 | 5 |
| hepatocellular carcinoma-associated gene TD26 (LOC55908) | 3 | chemokine (C-C motif) ligand 13 (CCL13) | 3 | complement factor H (CFH), transcript variant 2 | 5 |
| fucosyltransferase 6 (alpha (1,3) fucosyltransferase) (FUT6) | 3 | lysyl oxidase (LOX) | 3 | tripartite motif-containing 34 (TRIM34), transcript variant 1 | 5 |
| pappalysin 2 (PAPPA2), transcript variant 2 | 3 | retinoic acid induced 2 (RAI2) | 3 | regulator of G-protein signalling 18 (RGS18) | 5 |
| lactalbumin, alpha- (LALBA) | 3 | calpain 3, (p94) (CAPN3), transcript variant 1 | 3 | potassium channel tetramerisation domain containing 4 (KCTD4) | 5 |
| T-cell leukemia, homeobox 3 (TLX3) | 3 | otoraplin (OTOR) | 3 | lipase, endothelial (LIPG) | 5 |
| cholinergic receptor, muscarinic 2 (CHRM2), transcript variant 4 | 2 | tetratricopeptide repeat domain 13 (TTC13) | 3 | zinc finger CCCH-type, antiviral 1 (ZC3HAV1), transcript variant 2 | 5 |
| DnaJ (Hsp40) homolog, subfamily C, member 15 (DNAJC15) | 2 | inositol hexaphosphate kinase 3 (IHPK3) | 3 | glial cell derived neurotrophic factor (GDNF), transcript variant 3 | 5 |
| Rap guanine nucleotide exchange factor (GEF) 5 (RAPGEF5) | 2 | ATP-binding cassette, sub-family B (MDR/TAP), member 5, mRNA (cDNA clone IMAGE:5764845) | 3 | nuclear factor of kappa light polypeptide gene enhancer in B-cells 2 (p49/p100) (NFKB2) | 5 |
| testis specific, 10 (TSGA10) | 2 | leucine rich repeat containing 34 (LRRC34) | 3 | CD274 antigen (CD274) | 5 |
| repetin (RPTN) mRNA | 2 | selenophosphate synthetase 1 (SEPHS1) | 3 | tumor necrosis factor (ligand) superfamily, member 14 (TNFSF14), transcript variant 1 | 5 |
| disrupted in renal carcinoma 1 (DIRC1) | 2 | ATP-binding cassette, sub-family A (ABC1), member 6, mRNA (cDNA clone IMAGE:5172925), with apparent retained intron | 3 | inhibitor of growth family, member 2 (ING2) | 5 |
| asporin (LRR class 1) (ASPN) | 2 | eomesodermin homolog (Xenopus laevis) (EOMES) | 3 | spectrin, beta, non-erythrocytic 5 (SPTBN5) | 5 |
| protocadherin alpha 2 (PCDHA2), transcript variant 2 | 2 | H2B histone family, member W, testis-specific (H2BFWT) | 3 | ephrin-A1 (EFNA1), transcript variant 1 | 5 |
| similar to developmental pluripotency associated 5; embryonal stem cell specific gene 1 (LOC340168) | 2 | hypothetical MGC50722 (MGC50722) | 3 | pygopus homolog 2 (Drosophila) (PYGO2) | 5 |
| progestagen-associated endometrial protein (placental protein 14, pregnancy-associated endometrial alpha-2-globulin, alpha uterine protein) (PAEP), transcript variant 2 | 2 | hydroxysteroid (17-beta) dehydrogenase 1 (HSD17B1) | 3 | apolipoprotein B48 receptor (APOB48R) | 5 |
| taste receptor, type 2, member 13 (TAS2R13) | 2 | protocadherin beta 14 (PCDHB14) | 3 | calpain 9 (CAPN9), transcript variant 1 | 5 |
| kinesin family member 2B (KIF2B) | 2 | glutathione S-transferase M3 (brain) (GSTM3) | 3 | nuclear factor (erythroid-derived 2)-like 3 (NFE2L3) | 5 |
| transcription elongation regulator 1-like (TCERG1L) | 2 | flavin containing monooxygenase 1 (FMO1) | 3 | nuclear factor of kappa light polypeptide gene enhancer in B-cells inhibitor, zeta (NFKBIZ), transcript variant 1 | 5 |
| Soares fetal liver spleen 1NFLS cDNA clone IMAGp998I15411 ; IMAGE:210518, mRNA sequence | 2 | indoleamine-pyrrole 2,3 dioxygenase (INDO) | 3 | grainyhead-like 1 (Drosophila) (GRHL1), transcript variant 2 | 5 |
| cisplatin resistance-associated overexpressed protein (CROP), transcript variant 1 | 2 | ropporin, rhophilin associated protein 1 (ROPN1) | 3 | cytokine-like 1 (CYTL1) | 5 |
| a disintegrin-like and metalloprotease (reprolysin type) with thrombospondin type 1 motif, 10 (ADAMTS10) | 2 | thymic stromal co-transporter (TSCOT) | 3 | spermatogenesis associated 9 (SPATA9), transcript variant 1 | 5 |
| sarcoma antigen 1 (SAGE1) | 2 | G protein-coupled receptor 43 (GPR43) | 3 | superoxide dismutase 2, mitochondrial (SOD2), nuclear gene encoding mitochondrial protein, transcript variant 1 | 5 |
| integral membrane protein 2A (ITM2A) | 2 | chondroitin polymerizing factor (CHPF) | 3 | suppressor of cytokine signaling 1 (SOCS1) | 5 |
| G protein-coupled receptor kinase 7 (GRK7) | 2 | cadherin 9, type 2 (T1-cadherin) (CDH9) | 3 | dimethylarginine dimethylaminohydrolase 1, mRNA (cDNA clone MGC:45161 IMAGE:5189970) | 5 |
| vitrin (VIT) | 2 | 5-hydroxytryptamine (serotonin) receptor 1E (HTR1E) | 3 | neuritin 1 (NRN1) | 5 |
| zinc finger protein 638 (ZNF638), transcript variant 1 | 2 | ATP-binding cassette, sub-family A (ABC1), member 13 (ABCA13) | 3 | KIAA1404 protein (KIAA1404) | 5 |
| CSAG family, member 2 (CSAG2) | 2 | histone deacetylase 9 (HDAC9), transcript variant 5 | 3 | Spi-C transcription factor (Spi-1/PU1 related) (SPIC) | 5 |
| transmembrane protein 20 (TMEM20) | 2 | FK506 binding protein 6, 36kDa (FKBP6) | 3 | bromodomain, testis-specific (BRDT), transcript variant 1 | 5 |
| ribonuclease, RNase A family, 9 (non-active) (RNASE9) | 2 | potassium large conductance calcium-activated channel, subfamily M, beta member 2 (KCNMB2), transcript variant 2 | 3 | RNA-binding protein BRUNOL3 (BRUNOL3) mRNA | 5 |
| methyltransferase like 4 (METTL4) | 2 | nuclear receptor co-repressor 2 (NCOR2) | 3 | secreted and transmembrane 1 (SECTM1) | 5 |
| nucleosome assembly protein 1-like 2 (NAP1L2) | 2 | defensin, alpha 6, Paneth cell-specific (DEFA6) | 3 | arrestin domain containing 3, mRNA (cDNA clone MGC:8773 IMAGE:3908916) | 5 |
| DnaJ (Hsp40) homolog, subfamily C, member 6 (DNAJC6) | 2 | PAR-6 beta | 3 | ES cell associated trasnsript 1 (ECAT1) | 5 |
| tachykinin 3 (neuromedin K, neurokinin beta) (TAC3) | 2 | leukocyte cell derived chemotaxin 1 (LECT1), transcript variant 1 | 3 | serine (or cysteine) proteinase inhibitor, clade A (alpha-1 antiproteinase, antitrypsin), member 3 (SERPINA3) | 5 |
| N-acetyltransferase 8 (camello like) (NAT8) | 2 | DnaJ (Hsp40) homolog, subfamily C, member 11 (DNAJC11) | 3 | guanylate cyclase activator 2B (uroguanylin) (GUCA2B) | 5 |
| forkhead box P2 (FOXP2), transcript variant 4 | 2 | melanoma antigen family A, 10 (MAGEA10), transcript variant 2 | 3 | keratin associated protein 3-3 (KRTAP3-3) | 5 |
| olfactory receptor, family 2, subfamily W, member 1 (OR2W1) | 2 | lectin, galactoside-binding, soluble, 9 (galectin 9) (LGALS9), transcript variant long | 3 | DNA-damage inducible protein 1 (DDI1) | 5 |
| transcription elongation factor B polypeptide 3C (elongin A3) (TCEB3C) | 2 | SHQ1 homolog (S cerevisiae) (SHQ1) | 3 | DIRAS family, GTP-binding RAS-like 2 (DIRAS2) | 5 |
| protein expressed in prostate, ovary, testis, and placenta 2 (POTE2), transcript variant POTE-2B | 2 | killer cell lectin-like receptor subfamily A, member 1 (KLRA1) | 3 | cysteine-rich secretory protein 1 (CRISP1), transcript variant 1 | 5 |
| Neural cell adhesion molecule 1, 120 kDa isoform precursor variant protein | 2 | nuclear RNA export factor 3 (NXF3) | 3 | eukaryotic translation initiation factor 2-alpha kinase 2 (EIF2AK2) | 5 |
| serine (or cysteine) proteinase inhibitor, clade B (ovalbumin), member 2 (SERPINB2) | 2 | ficolin (collagen/fibrinogen domain containing lectin) 2 (hucolin) (FCN2), transcript variant SV1 | 3 | zinc finger protein 365 (ZNF365), transcript variant A | 5 |
| leucine rich repeat containing 36 (LRRC36) | 2 | potassium voltage-gated channel, Shal-related subfamily, member 2 (KCND2) | 3 | tumor necrosis factor receptor superfamily, member 14 (herpesvirus entry mediator) (TNFRSF14) | 5 |
| actin-like 7A (ACTL7A) | 2 | selectin ligand interactor cytoplasmic-1 (SLIC1), transcript variant 1 | 3 | lymphotoxin beta (TNF superfamily, member 3) (LTB), transcript variant 2 | 5 |
| cholecystokinin B receptor (CCKBR) | 2 | C-type lectin domain family 3, member A (CLEC3A) | 2 | leucine rich repeat containing 36 (LRRC36) | 5 |
| clathrin, heavy polypeptide-like 1 (CLTCL1), transcript variant 2 | 2 | TCR gamma alternate reading frame protein (TARP), nuclear gene encoding mitochondrial protein, transcript variant 2 | 2 | NADH dehydrogenase (ubiquinone) 1 beta subcomplex, 8, 19kDa (NDUFB8) | 5 |
| keratin associated protein 19-3 (KRTAP19-3) | 2 | leucine rich repeat containing 36 (LRRC36) | 2 | plasminogen activator, urokinase receptor (PLAUR), transcript variant 1 | 4 |
| sodium channel, voltage-gated, type X, alpha (SCN10A) | 2 | carcinoembryonic antigen-related cell adhesion molecule 3 (CEACAM3) | 2 | TRAF-type zinc finger domain containing 1 (TRAFD1) | 4 |
| regulator of G-protein signalling 1 (RGS1) | 2 | sperm acrosome associated 4 (SPACA4) | 2 | myosin, heavy polypeptide 13, skeletal muscle (MYH13) | 4 |
| jun D proto-oncogene (JUND) | 2 | pannexin 3 (PANX3) | 2 | LIM homeobox 1 (LHX1) | 4 |
| H2A histone family, member B3 (H2AFB3) | 2 | TGF-beta induced apotosis protein 2 (TAIP-2) | 2 | Fc fragment of IgE, high affinity I, receptor for; alpha polypeptide (FCER1A) | 4 |
| defensin, beta 105A (DEFB105A) | 2 | ATP-binding cassette, sub-family B (MDR/TAP), member 5 (ABCB5) | 2 | SPARC related modular calcium binding 2 (SMOC2) | 4 |
| chemokine (C-X-C motif) ligand 10 (CXCL10) | 2 | collagen, type III, alpha 1 (Ehlers-Danlos syndrome type IV, autosomal dominant) (COL3A1) | 2 | cytochrome P450, family 27, subfamily A, polypeptide 1 (CYP27A1), nuclear gene encoding mitochondrial protein | 4 |
| myeloid/lymphoid or mixed-lineage leukemia 4 (MLL4) | 2 | cartilage oligomeric matrix protein (COMP) | 2 | dickkopf-like 1 (soggy) (DKKL1) | 4 |
| phytanoyl-CoA hydroxylase interacting protein (PHYHIP) | 2 | G protein-coupled receptor 41 (GPR41) | 2 | tissue transglutaminase homologue {alternatively spliced} [human, erythroleukemia cell line HEL GM06141A, mRNA, 2362 nt] | 4 |
| T-cell activation kelch repeat protein (TA-KRP) | 2 | spermatogenesis associated 1 (SPATA1) | 2 | topoisomerase (DNA) III beta (TOP3B) | 4 |
| protein Z, vitamin K-dependent plasma glycoprotein (PROZ) | 2 | junctophilin 1 (JPH1) | 2 | plexin A4, B (PLXNA4B) | 4 |
| insulinoma-associated 2 (INSM2) | 2 | ATPase, Na+/K+ transporting, alpha 3 polypeptide (ATP1A3) | 2 | interleukin 32 (IL32), transcript variant 2 | 4 |
| keratin 6E (KRT6E) | 2 | lymphocyte glycoprotein T1/Leu-1 | 2 | interferon, gamma-inducible protein 30 (IFI30) | 4 |
| cullin 4B (CUL4B) | 2 | leucine rich repeat containing 7 (LRRC7) | 2 | apolipoprotein B mRNA editing enzyme, catalytic polypeptide-like 3G (APOBEC3G) | 4 |
| apolipoprotein A-V (APOA5) | 2 | retinoic acid receptor responder (tazarotene induced) 2 (RARRES2) | 2 | PDZ and LIM domain 4 (PDLIM4) | 4 |
| protocadherin beta 14 (PCDHB14) | 2 | sucrase-isomaltase (alpha-glucosidase) (SI) | 2 | HLA-G histocompatibility antigen, class I, G (HLA-G) | 4 |
| low density lipoprotein-related protein 2 (LRP2) | 2 | retinol dehydrogenase 8 (all-trans) (RDH8) | 2 | zinc finger protein 626 (ZNF626) | 4 |
| scinderin (SCIN) | 2 | thyrotropin-releasing hormone receptor (TRHR) | 2 | EH-domain containing 4 (EHD4) | 4 |
| defensin, beta 119 (DEFB119), transcript variant 1 | 2 | collagen, type XXIII, alpha 1 (COL23A1) | 2 | synaptotagmin 16 (SYT16 gene), splice variant 1 | 4 |
| docking protein 2, 56kDa (DOK2), transcript variant 2 | 2 | V-set and immunoglobulin domain containing 2 (VSIG2) | 2 | nuclear RNA export factor 3 (NXF3) | 4 |
| protocadherin alpha 9 (PCDHA9 gene) | 2 | p10-binding protein (Cep70) | 2 | angiopoietin-like 4 (ANGPTL4), transcript variant 1 | 4 |
| attractin-like 1 (ATRNL1) | 2 | a disintegrin-like and metalloprotease (reprolysin type) with thrombospondin type 1 motif, 6 (ADAMTS6) | 2 | tripartite motif-containing 34 (TRIM34), transcript variant 1 | 4 |
| zinc finger and BTB domain containing 37 (ZBTB37) | 2 | likely ortholog of kinesin light chain 2 (KLC2) | 2 | prostaglandin I2 (prostacyclin) synthase (PTGIS) | 4 |
| Janus kinase 2 (a protein tyrosine kinase) (JAK2) | 2 | cystatin F (leukocystatin) (CST7) | 2 | fibroblast growth factor 19 (FGF19) | 4 |
| delta-like 1 (Drosophila) (DLL1) | 2 | hornerin | 2 | nucleoporin 210kDa-like (NUP210L) | 4 |
| growth differentiation factor 10 (GDF10) | 2 | inter-alpha (globulin) inhibitor H5-like (ITIH5L) | 2 | G protein-coupled receptor, family C, group 5, member D (GPRC5D) | 4 |
| olfactory receptor, family 51, subfamily B, member 4 (OR51B4) | 2 | histone 1, H2ai (HIST1H2AI) | 2 | three prime repair exonuclease 1 (TREX1), transcript variant 1 | 4 |
| secreted frizzled-related protein 2 (SFRP2) | 2 | docking protein 2, 56kDa (DOK2), transcript variant 2 | 2 | tudor domain containing 7 (TDRD7) | 4 |
| amyotrophic lateral sclerosis 2 (juvenile) chromosome region, candidate 12 (ALS2CR12) | 2 | ES cell associated trasnsript 1 (ECAT1) | 2 | exostoses (multiple) 1 (EXT1) | 4 |
| prohibitin pseudogene, mRNA (cDNA clone MGC:20874 IMAGE:4547239) | 2 | thymic stromal lymphopoietin (TSLP), transcript variant 1 | 2 | transglutaminase 2 (C polypeptide, protein-glutamine-gamma-glutamyltransferase) (TGM2), transcript variant 1 | 4 |
| plasma membrane calcium ATPase isoform 2 (ATP2B2) mRNA | 2 | G-protein coupled receptor 88 (GPR88) | 2 | butyrophilin, subfamily 3, member A3 (BTN3A3), transcript variant 2 | 4 |
| serum amyloid A2 (SAA2) | 2 | p8 protein (candidate of metastasis 1) (P8) | 2 | bruno-like 5, RNA binding protein (Drosophila) (BRUNOL5), mRNA | 4 |
| phospholipase A2, group IIE (PLA2G2E) | 2 | vomeronasal 1 receptor 5 (VN1R5) | 2 | heparin-binding EGF-like growth factor (HBEGF) | 4 |
| histone 1, H4b (HIST1H4B) | 2 | CUB and Sushi multiple domains 1 (CSMD1) | 2 | complement component 1, s subcomponent (C1S), transcript variant 1 | 4 |
| caudal type homeo box transcription factor 1 (CDX1) | 2 | glutathione S-transferase A1 (GSTA1) | 2 | nuclear factor (erythroid-derived 2), 45kDa (NFE2) | 4 |
| CD20 receptor (S7) | 2 | heat shock protein, alpha-crystallin-related, B6 (HSPB6) | 2 | ATPase, Na+/K+ transporting, alpha 4 polypeptide (ATP1A4), transcript variant 1 | 4 |
| keratin 6 irs3 (K6IRS3) | 2 | lymphocyte antigen 75 (LY75) | 2 | absent in melanoma 2 (AIM2) | 4 |
| acetoacetyl-CoA synthetase-like, mRNA (cDNA clone IMAGE:3945810) | 2 | glycoprotein (transmembrane) nmb mRNA | 2 | ADP-ribosylation factor 7 (ARF7) | 4 |
| phenylalanine hydroxylase (PAH) | 2 | PALM2-AKAP2 protein (PALM2-AKAP2), transcript variant 2 | 2 | DC-STAMP domain containing 1 (DCST1) | 4 |
| taste receptor, type 2, member 9 (TAS2R9) | 2 | meprin A, beta (MEP1B) | 2 | hyaluronidase pseudogene 1 (HYALP1) | 4 |
| wingless-type MMTV integration site family, member 9B (WNT9B) | 2 | RAS-related on chromosome 22 (RRP22), transcript variant 1 | 2 | beta-carotene 15,15'-monooxygenase 1 (BCMO1) | 4 |
| myeloid/lymphoid or mixed-lineage leukemia (trithorax homolog, Drosophila) (MLL) | 2 | inversin (INVS), transcript variant 2 | 2 | nuclear factor of kappa light polypeptide gene enhancer in B-cells inhibitor, alpha (NFKBIA) | 4 |
| P antigen family, member 4 (prostate associated) (PAGE4) | 2 | mucin and cadherin-like (MUCDHL), transcript variant 4 | 2 | leukocyte immunoglobulin-like receptor, subfamily A (with TM domain), member 1 (LILRA1) | 4 |
| olfactory receptor, family 7, subfamily E, member 140 pseudogene, mRNA (cDNA clone IMAGE:5214442) | 2 | glutathione S-transferase A3 (GSTA3) | 2 | eukaryotic translation initiation factor 2C, 1 (EIF2C1) | 4 |
| protein kinase C substrate 80K-H (PRKCSH), transcript variant 2 | 2 | leucine rich repeat containing 2 (LRRC2) | 2 | G protein-coupled receptor 109B (GPR109B) | 4 |
| ryanodine receptor 2 (cardiac) (RYR2) | 2 | fatty acid binding protein 2, intestinal (FABP2) | 2 | lysyl oxidase (LOX) | 4 |
| retinoic acid induced 2 (RAI2) | 2 | S100 calcium binding protein A8 (calgranulin A) (S100A8) | 2 | cytochrome c oxidase subunit VIa polypeptide 2 (COX6A2), nuclear gene encoding mitochondrial protein | 4 |
| PDZ domain containing 3 (PDZK3), transcript variant 2 | 2 | SRY (sex determining region Y)-box 10 (SOX10) | 2 | pancreatic lipase (PNLIP) | 4 |
| cysteine-rich secretory protein 2 (CRISP2) | 2 | creatine kinase, mitochondrial 2 (sarcomeric) (CKMT2), nuclear gene encoding mitochondrial protein | 2 | GTP cyclohydrolase 1 (dopa-responsive dystonia) (GCH1), transcript variant 1 | 4 |
| epsin 3 (EPN3) | 2 | retinal degeneration, slow (RDS) | 2 | protocadherin 12 (PCDH12) | 4 |
| G protein-coupled receptor, family C, group 5, member D (GPRC5D) | 2 | colony stimulating factor 2 receptor, beta, low-affinity (granulocyte-macrophage), mRNA (cDNA clone MGC:87425 IMAGE:30344148) | 2 | dual specificity phosphatase 5 (DUSP5) | 4 |
| vitelliform macular dystrophy 2 (Best disease, bestrophin) (VMD2) | 2 | 4-aminobutyrate aminotransferase (GABAT) mRNA | 2 | chemokine (C-C motif) ligand 8 (CCL8) | 4 |
| AF4/FMR2 family, member 3 (AFF3), transcript variant 1 | 2 | colony stimulating factor 1 (macrophage) (CSF1), transcript variant 1 | 2 | major histocompatibility complex, class I, E (HLA-E) | 4 |
| clone IMAGE:5754998 | 2 | pregnancy-zone protein (PZP) | 2 | SH3-domain GRB2-like 2 (SH3GL2) | 4 |
| protein kinase, cGMP-dependent, type II (PRKG2) | 2 | tachykinin receptor 2 (TACR2) | 2 | CD83 antigen (activated B lymphocytes, immunoglobulin superfamily) (CD83) | 4 |
| calcium channel, voltage-dependent, gamma subunit 8 (CACNG8) | 2 | RIM binding protein 2 (KIAA0318) | 2 | scotin (SCOTIN) | 4 |
| ankyrin repeat domain 45 (ANKRD45) | 2 | GDNF family receptor alpha 2 (GFRA2) | 2 | F-box protein 6 (FBXO6) | 4 |
| G protein-coupled receptor 18 (GPR18) | 2 | proline-serine-threonine phosphatase interacting protein 1 (PSTPIP1) | 2 | ring finger protein 148 (RNF148) | 4 |
| adrenergic, beta-1-, receptor (ADRB1) | 2 | guanylate binding protein family, member 6 (GBP6) | 2 | orosomucoid 1 (ORM1) | 4 |
| angiopoietin 1 (ANGPT1), transcript variant 1 | 2 | methylthioadenosine phosphorylase (MTAP) mRNA | 2 | lipopolysaccharide binding protein (LBP) | 4 |
| interleukin 1 family, member 9 (IL1F9) | 2 | glutaminase 2 (liver, mitochondrial) (GLS2), nuclear gene encoding mitochondrial protein, transcript variant 1 | 2 | N-ethylmaleimide-sensitive factor attachment protein, alpha (NAPA) | 4 |
| t-complex-associated-testis-expressed 3 (TCTE3) | 2 | myelin-associated oligodendrocyte basic protein (MOBP), transcript variant 1 | 2 | fms-related tyrosine kinase 3 ligand (FLT3LG) | 4 |
| RAS-like, family 11, member B (RASL11B) | 2 | cardiac-MyBP-C associated Ca/CaM kinase (MLCK) | 2 | glutathione peroxidase 5 (epididymal androgen-related protein) (GPX5), transcript variant 2 | 4 |
| armadillo repeat containing, X-linked 2 (ARMCX2) | 2 | apolipoprotein B mRNA editing enzyme, catalytic polypeptide-like 3A (APOBEC3A) | 2 | CD300 antigen like family member G (CD300LG) | 4 |
| zinc finger protein 117 (HPF9) (ZNF117) | 2 | PHD finger protein 7 (PHF7), transcript variant 1 | 2 | cyclic nucleotide gated channel alpha 1 (CNGA1), mRNA | 4 |
| microtubule associated monoxygenase, calponin and LIM domain containing 2 (MICAL2) | 2 | hyaluronidase pseudogene 1 (HYALP1) | 2 | olfactory receptor, family 2, subfamily A, member 4 (OR2A4) | 4 |
| EPS8-like 3 (EPS8L3), transcript variant 1 | 2 | formyl peptide receptor 1 (FPR1) | 2 | phosphatidylinositol 4-kinase type-II beta (PI4K2B) | 4 |
| DnaJ (Hsp40) homolog, subfamily B, member 7 (DNAJB7) | 2 | cancer antigen 1 (CAGE1), transcript variant 2 | 2 | zinc metalloprotease ADAMTS7 (ADAMTS7) mRNA | 4 |
| leukocyte immunoglobulin-like receptor, subfamily A (with TM domain), member 5 (LILRA5), transcript variant 1 | 2 | GIPC PDZ domain containing family, member 2 (GIPC2) | 2 | serine (or cysteine) proteinase inhibitor, clade B (ovalbumin), member 4 (SERPINB4) | 4 |
| estrogen receptor 2 (ER beta) (ESR2) | 2 | carboxypeptidase, vitellogenic-like (CPVL), transcript variant 1 | 2 | follistatin (FST), transcript variant FST317 | 4 |
| testis-specific protein TSP-NY (TSP-NY), transcript variant 1 | 2 | chromogranin B (secretogranin 1) (CHGB) | 2 | ubiquitin specific protease 15 (USP15) | 4 |
| chemokine (C-C motif) ligand 5 (CCL5) | 2 | LIM homeobox 5 (LHX5) | 2 | papillary thyroid carcinoma-encoded protein mRNA | 4 |
| SRY (sex determining region Y)-box 10 (SOX10) | 2 | zinc finger protein 19 (KOX 12) (ZNF19) | 2 | poliovirus receptor-related 2 (herpesvirus entry mediator B) (PVRL2) | 4 |
| 5-hydroxytryptamine (serotonin) receptor 4 (HTR4), transcript variant b | 2 | zinc finger protein 15-like 1 (KOX 8) (ZNF15L1) | 2 | retinoic acid early transcript 1L (RAET1L) | 4 |
| prokineticin 2 (PROK2) | 2 | CD14 antigen (CD14) | 2 | calcium binding protein 1 (calbrain) (CABP1), transcript variant 1 | 4 |
| RAB3C, member RAS oncogene family (RAB3C) | 2 | transient receptor potential cation channel, subfamily C, member 3 (TRPC3) | 2 | dipeptidylpeptidase 4 (CD26, adenosine deaminase complexing protein 2) (DPP4) | 4 |
| killer cell lectin-like receptor subfamily A, member 1 (KLRA1) | 2 | ubiquitin-conjugating enzyme E2G 2 (UBC7 homolog, yeast) (UBE2G2), transcript variant 1 | 2 | cholesterol 25-hydroxylase (CH25H) | 4 |
| myosin binding protein C, slow type (MYBPC1), transcript variant 1 | 2 | sulfotransferase family, cytosolic, 1B, member 1 (SULT1B1) | 2 | similar to autoantigen La (LOC343232), mRNA | 4 |
| mutS homolog 4 (E coli) (MSH4) | 2 | zinc finger protein 365 (ZNF365), transcript variant A | 2 | phosphoglucomutase 5 (PGM5) | 4 |
| myosin, heavy polypeptide 2, skeletal muscle, adult (MYH2) | 2 | kelch repeat and BTB (POZ) domain containing 10 (KBTBD10) | 2 | zinc finger, MYND-type containing 15 (ZMYND15) | 4 |
| interferon, gamma-inducible protein 16 (IFI16) | 2 | SRY (sex determining region Y)-box 3 (SOX3) | 2 | tumor necrosis factor, alpha-induced protein 2 (TNFAIP2) | 4 |
| C1q and tumor necrosis factor related protein 2 (C1QTNF2) | 2 | CEI protein (CEI) | 2 | early growth response 4 (EGR4) | 4 |
| RAS-like, family 11, member A (RASL11A) | 2 | UDP glucuronosyltransferase 2 family, polypeptide B28 (UGT2B28) | 2 | F-box protein 32 (FBXO32), transcript variant 1 | 4 |
| islet amyloid polypeptide (IAPP) | 2 | myeloid/lymphoid or mixed-lineage leukemia (trithorax homolog, Drosophila); translocated to, 7 (MLLT7) | 2 | claudin 23 (CLDN23) | 4 |
| zinc finger protein 268 (ZNF268) | 2 | voltage gated channel like 1 (VGCNL1) | 2 | GRB2-associated binding protein 1 (GAB1), transcript variant 2 | 4 |
| TAF6 RNA polymerase II, TATA box binding protein (TBP)-associated factor, 80kDa (TAF6), transcript variant 1 | 2 | frizzled homolog 10 (Drosophila) (FZD10) | 2 | chemokine (C-X-C motif) ligand 3 (CXCL3) | 4 |
| adenosine monophosphate deaminase 1 (isoform M) (AMPD1) | 2 | mRNA; cDNA DKFZp686F1745 (from clone DKFZp686F1745) | 2 | chemokine (C-X-C motif) ligand 1 (melanoma growth stimulating activity, alpha) (CXCL1) | 4 |
| leucine rich repeat transmembrane neuronal 4 (LRRTM4) | 2 | testes-specific protease 50 (TSP50) | 2 | zinc finger protein 100, mRNA (cDNA clone IMAGE:3344728), with apparent retained intron | 4 |
| zinc finger protein 215 (ZNF215) | 2 | protein tyrosine phosphatase, receptor type, C (PTPRC), transcript variant 1 | 2 | interferon, alpha 1 (IFNA1) | 4 |
| vestigial like 1 (Drosophila) (VGLL1) | 2 | gonadotropin-releasing hormone receptor [human, mRNA, 2160 nt] | 2 | proteasome (prosome, macropain) activator subunit 2 (PA28 beta) (PSME2) | 4 |
| chemokine (C-C motif) ligand 23 (CCL23), transcript variant CKbeta8-1 | 2 | protein tyrosine phosphatase, receptor type, E (PTPRE), transcript variant 2 | 2 | lymphocyte antigen 6 complex, locus E (LY6E) | 4 |
| CD7 antigen (p41) (CD7) | 2 | T-cell activation NFKB-like protein (TA-NFKBH) | 2 | pepsinogen 5, group I (pepsinogen A) (PGA5) | 4 |
| septin 3 (SEPT3), transcript variant A | 2 | desmin (DES) | 2 | ATPase, H+ transporting, lysosomal 42kDa, V1 subunit C isoform 2 (ATP6V1C2) | 4 |
| zinc finger protein 224 (ZNF224) | 2 | histone 1, H1t (HIST1H1T) | 2 | melanoma-derived leucine zipper, extra-nuclear factor (MLZE) | 4 |
| interleukin 1 receptor accessory protein-like 1 (IL1RAPL1) | 2 | desmocollin 1 (DSC1), transcript variant Dsc1a | 2 | zinc finger protein 577 (ZNF577) | 4 |
| X Kell blood group precursor-related, Y-linked (XKRY) | 2 | cathelicidin antimicrobial peptide (CAMP) | 2 | inactivation escape 1 (INE1) | 4 |
| growth differentiation factor 5 (cartilage-derived morphogenetic protein-1) (GDF5) | 2 | tripartite motif-containing 22 (TRIM22) | 2 | interleukin 1 receptor accessory protein (IL1RAP), transcript variant 1 | 4 |
| erythrocyte membrane protein band 41 like 4A (EPB41L4A) | 2 | thymocyte protein thy28 (THY28), transcript variant 3 | 2 | Friend leukemia virus integration 1 (FLI1) | 4 |
| transmembrane protein 28 (TMEM28) | 2 | ataxin 2-binding protein 1 (A2BP1), transcript variant 3 | 2 | killer cell lectin-like receptor subfamily A, member 1 (KLRA1) | 4 |
| AP1 gamma subunit binding protein 1 (AP1GBP1), transcript variant 2 | 2 | t-complex 11 (mouse) (TCP11) | 2 | growth hormone 1 (GH1), transcript variant 1 | 4 |
| C-type lectin domain family 2, member A (CLEC2A) | 2 | trypsin X3 (TRY1) | 2 | syntaxin 11 (STX11) | 4 |
| type I hair keratin KA36 (KA36) | 2 | methyltransferase like 4 (METTL4) | 2 | histone 1, H2bg (HIST1H2BG) | 4 |
| ELMO domain containing 1 (ELMOD1) | -2 | proteolipid protein 1 (Pelizaeus-Merzbacher disease, spastic paraplegia 2, uncomplicated) (PLP1), transcript variant 1 | 2 | neurotensin receptor 1 (high affinity) (NTSR1) | 4 |
| myelin-associated oligodendrocyte basic protein (MOBP), transcript variant 1 | -2 | serine (or cysteine) proteinase inhibitor, clade B (ovalbumin), member 4 (SERPINB4) | 2 | phosphorylase, glycogen; muscle (McArdle syndrome, glycogen storage disease type V) (PYGM) | 4 |
| pan-hematopoietic expression (PHEMX), transcript variant 3 | -2 | PRH2 locus salivary proline-rich protein mRNA (Pr1 allele) | 2 | v-jun sarcoma virus 17 oncogene homolog (avian) (JUN) | 4 |
| chemokine (C-C motif) ligand 1 (CCL1) | -2 | tropomodulin 2 (neuronal) (TMOD2) | 2 | hyaluronan and proteoglycan link protein 3 (HAPLN3) | 4 |
| G protein-coupled receptor 41 (GPR41) | -3 | agouti related protein homolog (mouse) (AGRP), transcript variant 1 | 2 | TBC1 domain family, member 10C (TBC1D10C) | 4 |
| podocan (PODN) | -3 | CD180 antigen (CD180) | 2 | tripartite motif-containing 14 (TRIM14), transcript variant 4 | 3 |
| zinc finger protein 273 (ZNF273) | -3 | DR1-associated protein 1 (negative cofactor 2 alpha) (DRAP1) | 2 | plasma glutamate carboxypeptidase (PGCP) | 3 |
| armadillo repeat containing 4 (ARMC4) | -4 | zinc finger protein 14 (KOX 6) (ZNF14) | 2 | Kruppel-like factor 4 (gut) (KLF4) | 3 |
| DEAH (Asp-Glu-Ala-Asp/His) box polypeptide 57 (DHX57), transcript variant 3 | -4 | cDNA clone IMAGE:5296456 | 2 | major histocompatibility complex, class II, DR alpha precursor variant protein | 3 |
| proprotein convertase subtilisin/kexin type 5 (PCSK5) | -4 | clone IMAGE:5404753 | 2 | cartilage oligomeric matrix protein (COMP) | 3 |
| protein phosphatase 2C, magnesium-dependent, catalytic subunit (PPM2C), nuclear gene encoding mitochondrial protein | -4 | UI-H-FT1-bka-d-03-0-UI.s1 NCI_CGAP_FT1 cDNA clone UI-H-FT1-bka-d-03-0-UI 3', mRNA sequence | 2 | chemokine (C-X-C motif) ligand 5 (CXCL5) | 3 |
| keratin associated protein 13-2 (KRTAP13-2), nuclear gene encoding mitochondrial protein | -5 | DEAD-box protein CAGE (CAGE) mRNA | 2 | signal-transducing adaptor protein-2 (STAP2), transcript variant 1 | 3 |
| Fc fragment of IgG, low affinity IIIb, receptor (CD16b) (FCGR3B) | -5 | UDP-GalNAc:polypeptide N-acetylgalactosaminyl transferase (GalNAc-T3) | 2 | myeloid/lymphoid or mixed-lineage leukemia (trithorax homolog, Drosophila); translocated to, 7 (MLLT7) | 3 |
| angiotensin I converting enzyme (peptidyl-dipeptidase A) 2 (ACE2) | -6 | homeo box (expressed in ES cells) 1 (HESX1) | 2 | zinc finger protein 541 (ZNF541) | 3 |
| SLIT and NTRK-like family, member 1 (SLITRK1) | -6 | ectonucleotide pyrophosphatase/phosphodiesterase 4 (putative function) (ENPP4) | 2 | Fc fragment of IgE, high affinity I, receptor for; gamma polypeptide (FCER1G) | 3 |
| inositol hexaphosphate kinase 1 (IHPK1), transcript variant 1 | -7 | dynein, axonemal, heavy polypeptide 9 (DNAH9), transcript variant 1 | 2 | myelin-associated oligodendrocyte basic protein (MOBP), transcript variant 1 | 3 |
| transmembrane protease, serine 12 (TMPRSS12) | -7 | ubiquitin associated and SH3 domain containing, A (UBASH3A), transcript variant 1 | 2 | pentraxin-related gene, rapidly induced by IL-1 beta (PTX3) | 3 |
| neutrophil cytosolic factor 4, 40kDa (NCF4), transcript variant 1 | -8 | G protein-coupled bile acid receptor 1 (GPBAR1) | 2 | SHQ1 homolog (S cerevisiae) (SHQ1) | 3 |
| endothelin converting enzyme 2 (ECE2) | -9 | phytanoyl-CoA hydroxylase interacting protein (PHYHIP) | 2 | 2'-5'-oligoadenylate synthetase 3, 100kDa (OAS3) | 3 |
| neuropeptide Y receptor Y5 (NPY5R) | -9 | transforming growth factor, beta 3 (TGFB3) | 2 | deleted in lymphocytic leukemia 7 (DLEU7) mRNA | 3 |
| RAS-related on chromosome 22 (RRP22), transcript variant 1 | -10 | nuclear receptor subfamily 1, group I, member 2 (NR1I2), transcript variant 3 | 2 | phorbol-12-myristate-13-acetate-induced protein 1 (PMAIP1) | 3 |
| caudal type homeo box transcription factor 4 (CDX4) | -12 | FXYD domain containing ion transport regulator 7 (FXYD7) | 2 | neutrophil cytosolic factor 1 (47kDa, chronic granulomatous disease, autosomal 1) (NCF1) | 3 |
| intramembrane protease 5 (IMP5) | -12 | activating transcription factor 7 interacting protein 2 (ATF7IP2) | 2 | proteasome (prosome, macropain) activator subunit 2 (PA28 beta) (PSME2) | 3 |
| putative peroxisome microbody protein 175.1 (LOC51051), mRNA | -14 | defensin, beta 119 (DEFB119), transcript variant 3 | 2 | ST6 (alpha-N-acetyl-neuraminyl-2,3-beta-galactosyl-1,3)-N-acetylgalactosaminide alpha-2,6-sialyltransferase 1 (ST6GALNAC1) | 3 |
| transmembrane emp24 protein transport domain containing 6 (TMED6) | -14 | transmembrane protease, serine 11B (TMPRSS11B) | 2 | mel transforming oncogene-like 1 (MELL1) | 3 |
| roundabout, axon guidance receptor, homolog 2 (Drosophila) (ROBO2) | -15 | myosin binding protein C, fast type (MYBPC2) | 2 | phospholipase A2, group IVC (cytosolic, calcium-independent) (PLA2G4C) | 3 |
| striated muscle activator of Rho-dependent signaling (STARS) | -19 | olfactomedin-like 3 (OLFML3) | 2 | histone 1, H4g (HIST1H4G) | 3 |
| UDP-Gal:betaGlcNAc beta 1,3-galactosyltransferase, polypeptide 1 (B3GALT1) | -20 | kin of IRRE like 2 (Drosophila) (KIRREL2), transcript variant 3 | 2 | leukocyte cell derived chemotaxin 1 (LECT1), transcript variant 1 | 3 |
| dynein, axonemal, heavy polypeptide 9 (DNAH9), transcript variant 1 | -20 | caspase 1, apoptosis-related cysteine protease (interleukin 1, beta, convertase) (CASP1), transcript variant alpha | 2 | HCV F-transactivated protein 1 (LOC401152) | 3 |
| Fas apoptotic inhibitory molecule 3 (FAIM3) | -21 | U2AF homology motif (UHM) kinase 1 (UHMK1) | 2 | transmembrane protein 27 (TMEM27) | 3 |
| zinc finger and BTB domain containing 41 (ZBTB41) | -27 | replication factor C (activator 1) 3, 38kDa (RFC3), transcript variant 1 | 2 | fibroblast growth factor 14 (FGF14), transcript variant 1 | 3 |
| catenin (cadherin-associated protein), alpha 3 (CTNNA3) | -35 | chemokine (C-C motif) receptor 3 (CCR3), transcript variant 1 | 2 | tripartite motif-containing 2 (TRIM2) | 3 |
| ets variant gene 1 (ETV1) | -35 | defensin, beta 127 (DEFB127) | 2 | protein tyrosine phosphatase, receptor type, E (PTPRE), transcript variant 2 | 3 |
| LSM11, U7 small nuclear RNA associated (LSM11) | -37 | metallothionein 1B (functional) (MT1B) | 2 | calpain 3, (p94) (CAPN3), transcript variant 1 | 3 |
| similar to immunoglobulin heavy chain variable region, clone IMAGE:5296752 | -44 | calicin (CCIN) | 2 | PDLIM1 interacting kinase 1 like (PDIK1L) | 3 |
| HMT1 hnRNP methyltransferase-like 4 (S cerevisiae) (HRMT1L4) | -50 | hemopoietic cell kinase (HCK) | 2 | glioma-associated oncogene homolog 1 (zinc finger protein) (GLI1) | 3 |
| inducible T-cell co-stimulator ligand (ICOSLG) | -50 | killer cell immunoglobulin-like receptor, two domains, long cytoplasmic tail, 4 (KIR2DL4) | 2 | LSM11, U7 small nuclear RNA associated (LSM11) | 3 |
| FLJ46154 protein (FLJ46154) | -67 | transglutaminase 3 (E polypeptide, protein-glutamine-gamma-glutamyltransferase) (TGM3) | 2 | inhibitor of DNA binding 4, dominant negative helix-loop-helix protein (ID4) | 3 |
| major histocompatibility complex, class II, DR alpha precursor variant protein | -75 | zinc finger, CCHC domain containing 12 (ZCCHC12) | -2 | zinc finger protein 267 (ZNF267), transcript variant 498723 | 3 |
| zinc finger protein 229 (ZNF229) | -139 | albumin (ALB) | -2 | eomesodermin homolog (Xenopus laevis) (EOMES) | 3 |
| cytoplasmic dynein 2 heavy chain mRNA | -337 | similar to CTAGE-5B protein (LOC341689), mRNA | -3 | tetraspanin 2, mRNA (cDNA clone MGC:22863 IMAGE:4042036) | 3 |
|  |  | zinc finger protein 610 (ZNF610) | -3 | actinin, alpha 2 (ACTN2) | 3 |
|  |  | prokineticin 2 (PROK2) | -3 | olfactomedin-like 3 (OLFML3) | 3 |
|  |  | DEAD (Asp-Glu-Ala-Asp) box polypeptide 4 (DDX4), transcript variant 2 | -3 | G protein-coupled bile acid receptor 1 (GPBAR1) | 3 |
|  |  | DEAH (Asp-Glu-Ala-Asp/His) box polypeptide 57 (DHX57), transcript variant 3 | -3 | BCL2-like 13 (apoptosis facilitator) (BCL2L13), nuclear gene encoding mitochondrial protein | 3 |
|  |  | a disintegrin-like and metalloprotease (reprolysin type) with thrombospondin type 1 motif, 20 (ADAMTS20), transcript variant 1 | -4 | tumor necrosis factor receptor superfamily, member 11a, NFKB activator (TNFRSF11A) | 3 |
|  |  | Usher syndrome 3A (USH3A), transcript variant 1 | -4 | DnaJ (Hsp40) homolog, subfamily C, member 15 (DNAJC15) | 3 |
|  |  | SPANX family, member N4 (SPANX-N4) | -4 | F1(P4)1 CFS leukocyte Homo sapiens cDNA, mRNA sequence | 3 |
|  |  | cathepsin E (CTSE), transcript variant 1 | -4 | transcobalamin II; macrocytic anemia (TCN2) | 3 |
|  |  | regulatory factor X, 4 (influences HLA class II expression) (RFX4), transcript variant 3 | -5 | REST corepressor 1 (RCOR1) | 3 |
|  |  | dermcidin (DCD) | -5 | butyrophilin, subfamily 3, member A1 (BTN3A1), transcript variant 2 | 3 |
|  |  | TU3A protein (TU3A) | -5 | angiotensin I converting enzyme (peptidyl-dipeptidase A) 1 (ACE), transcript variant 1 | 3 |
|  |  | nuclear receptor subfamily 5, group A, member 1 (NR5A1) | -6 | breast carcinoma amplified sequence 1 (BCAS1) | 3 |
|  |  | low density lipoprotein-related protein 1B (deleted in tumors) (LRP1B) | -6 | interleukin 12A (natural killer cell stimulatory factor 1, cytotoxic lymphocyte maturation factor 1, p35) (IL12A) | 3 |
|  |  | membrane-spanning 4-domains, subfamily A, member 6A (MS4A6A), transcript variant 2 | -6 | hypothetical protein FLJ25680 (FLJ25680) | 3 |
|  |  | diacylglycerol kinase, iota (DGKI) | -7 | G protein-coupled receptor 41 (GPR41) | 3 |
|  |  | homeo box C12 (HOXC12) | -7 | zinc finger protein 23 (KOX 16) (ZNF23) | 3 |
|  |  | P antigen family, member 2B (PAGE2B) | -8 | histidine decarboxylase (HDC) | 3 |
|  |  | hemicentin 1 (HMCN1) | -8 | chemokine (C-X-C motif) ligand 6 (granulocyte chemotactic protein 2) (CXCL6) | 3 |
|  |  | inter-alpha (globulin) inhibitor H4 (plasma Kallikrein-sensitive glycoprotein) (ITIH4) | -8 | mucin and cadherin-like (MUCDHL), transcript variant 4 | 3 |
|  |  | dopachrome tautomerase (dopachrome delta-isomerase, tyrosine-related protein 2) (DCT) | -9 | Rho GTPase activating protein 20 (ARHGAP20) | 3 |
|  |  | integrin, beta 6 (ITGB6) | -9 | MYH9 full length open reading frame (ORF) cDNA clone (cDNA clone C22ORF:pGEMMYH9) | 3 |
|  |  | Sp6 transcription factor (SP6) | -9 | EPH receptor B6 (EPHB6) | 3 |
|  |  | histatin 3 (HTN3) | -10 | sphingosine kinase 1 (SPHK1), transcript variant 1 | 3 |
|  |  | chordin-like 1 (CHRDL1) | -11 | epiregulin (EREG) | 3 |
|  |  | vasoactive intestinal peptide receptor 2 (VIPR2) | -11 | H1 histone family, member 0 (H1F0) | 3 |
|  |  | guanylate binding protein 1, interferon-inducible, 67kDa (GBP1) | -12 | inducible T-cell co-stimulator ligand (ICOSLG) | 3 |
|  |  | lipase, member I (LIPI) | -12 | ankyrin repeat, SAM and basic leucine zipper domain containing 1 (ASZ1) | 3 |
|  |  | purine-rich element binding protein G (PURG), transcript variant A | -13 | fetal brain adenylyl cyclase mRNA, 3' end | 3 |
|  |  | transmembrane protease, serine 12 (TMPRSS12) | -14 | armadillo repeat containing, X-linked 1 (ARMCX1) | 3 |
|  |  | zinc finger protein 560 (ZNF560) | -17 | lectin, galactoside-binding, soluble, 9 (galectin 9) pseudogene, mRNA (cDNA clone MGC:90361 IMAGE:5222492) | 3 |
|  |  | distal-less homeo box 3 (DLX3) | -17 | leucine rich repeat containing 34 (LRRC34) | 3 |
|  |  | interleukin 9 (IL9) | -18 | collagen, type III, alpha 1 (Ehlers-Danlos syndrome type IV, autosomal dominant) (COL3A1) | 3 |
|  |  | RGM domain family, member A (RGMA) | -18 | dickkopf homolog 3 (Xenopus laevis) (DKK3), transcript variant 1 | 3 |
|  |  | microseminoprotein, beta- (MSMB), transcript variant PSP57 | -19 | zinc finger protein 229 (ZNF229) | 3 |
|  |  | casein alpha s1 (CSN1S1), transcript variant 1 | -21 | semenogelin II (SEMG2) | 3 |
|  |  | nucleosome assembly protein 1-like 2 (NAP1L2) | -26 | G protein-coupled receptor 51 (GPR51) | 3 |
|  |  | interleukin 1, alpha (IL1A) | -85 | TNFAIP3 interacting protein 1 (TNIP1) | 3 |
|  |  | gamma-aminobutyric acid (GABA) A receptor, alpha 1 (GABRA1) | -107 | myeloid/lymphoid or mixed-lineage leukemia (trithorax homolog, Drosophila) (MLL) | 3 |
|  |  |  |  | brain secretory protein hSec10p (HSEC10) mRNA | 3 |
|  |  |  |  | butyrophilin, subfamily 3, member A2 (BTN3A2) | 3 |
|  |  |  |  | basic leucine zipper nuclear factor 1 (JEM-1) (BLZF1) | 3 |
|  |  |  |  | 5'-nucleotidase, ecto (CD73) (NT5E) | 3 |
|  |  |  |  | transducin-like enhancer of split 4 (E(sp1) homolog, Drosophila) (TLE4) | 3 |
|  |  |  |  | dehydrogenase/reductase (SDR family) member 6 (DHRS6) | 3 |
|  |  |  |  | Cas-Br-M (murine) ecotropic retroviral transforming sequence c (CBLC) | 3 |
|  |  |  |  | caspase recruitment domain family, member 15 (CARD15) | 3 |
|  |  |  |  | Fc fragment of IgA, receptor for (FCAR), transcript variant 6 | 3 |
|  |  |  |  | kinase suppressor of ras 2 (KSR2) | 3 |
|  |  |  |  | alanine-glyoxylate aminotransferase 2-like 1 (AGXT2L1) | 3 |
|  |  |  |  | chloride intracellular channel 4 (CLIC4) | 3 |
|  |  |  |  | nuclear localized factor 1 (NLF1) | 3 |
|  |  |  |  | zinc finger protein 20 (KOX 13) (ZNF20) | 3 |
|  |  |  |  | NK3 transcription factor related, locus 1 (Drosophila) (NKX3-1) | 3 |
|  |  |  |  | GTP binding protein 1 (GTPBP1) | 3 |
|  |  |  |  | T-cell leukemia/lymphoma 6 (TCL6), transcript variant TCL6a1 | 3 |
|  |  |  |  | tripartite motif-containing 5 (TRIM5), transcript variant alpha | 3 |
|  |  |  |  | zinc finger protein 272 (ZNF272) | 3 |
|  |  |  |  | FERM domain containing 5 (FRMD5) | 3 |
|  |  |  |  | complement component 1, q subcomponent, gamma polypeptide (C1QG) | 3 |
|  |  |  |  | DnaJ (Hsp40) homolog, subfamily C, member 6 (DNAJC6) | 3 |
|  |  |  |  | ectonucleotide pyrophosphatase/phosphodiesterase 1 (ENPP1) | 3 |
|  |  |  |  | butyrophilin, subfamily 2, member A1 (BTN2A1), transcript variant 1 | 3 |
|  |  |  |  | hairy and enhancer of split 4 (Drosophila) (HES4) | 3 |
|  |  |  |  | microtubule associated serine/threonine kinase-like (MASTL) | 3 |
|  |  |  |  | ATPase, Na+/K+ transporting, alpha 3 polypeptide (ATP1A3) | 3 |
|  |  |  |  | chemokine (C-X-C motif) receptor 4 (CXCR4), transcript variant 2 | 3 |
|  |  |  |  | apoptosis-associated nuclear protein PHLDA1 (PHLDA1) mRNA | 3 |
|  |  |  |  | zinc finger protein 483 (ZNF483), transcript variant 1 | 3 |
|  |  |  |  | lymphocyte antigen 86 (LY86) | 3 |
|  |  |  |  | glial fibrillary acidic protein (GFAP) | 3 |
|  |  |  |  | UDP-GlcNAc:betaGal beta-1,3-N-acetylglucosaminyltransferase 1 (B3GNT1), transcript variant 2 | 3 |
|  |  |  |  | nuclear factor of kappa light polypeptide gene enhancer in B-cells inhibitor, epsilon (NFKBIE), mRNA | 3 |
|  |  |  |  | similar to Dynein heavy chain at 16F (LOC200383) | 3 |
|  |  |  |  | putative peroxisome microbody protein 175.1 (LOC51051), mRNA | 3 |
|  |  |  |  | zinc finger protein 276 homolog (mouse) (ZFP276) | 3 |
|  |  |  |  | ADP-ribosylation-like factor 8 mRNA | 3 |
|  |  |  |  | CCAAT/enhancer binding protein (C/EBP), delta (CEBPD) | 3 |
|  |  |  |  | ubiquitin-conjugating enzyme E2G 2 (UBC7 homolog, yeast) (UBE2G2), transcript variant 1 | 3 |
|  |  |  |  | tubulin, beta polypeptide 4, member Q (TUBB4Q) | 3 |
|  |  |  |  | selenophosphate synthetase 1 (SEPHS1) | 3 |
|  |  |  |  | a disintegrin and metalloproteinase domain 19 (meltrin beta) (ADAM19), transcript variant 2 | 3 |
|  |  |  |  | hyaluronan synthase 2 (HAS2) | 3 |
|  |  |  |  | DnaJ (Hsp40) homolog, subfamily A, member 1 (DNAJA1) | 3 |
|  |  |  |  | ATP-binding cassette, sub-family B (MDR/TAP), member 9 (ABCB9), transcript variant 4 | 3 |
|  |  |  |  | GTPase, IMAP family member 1 (GIMAP1) | 3 |
|  |  |  |  | serine (or cysteine) proteinase inhibitor, clade E (nexin, plasminogen activator inhibitor type 1), member 1 (SERPINE1) | 3 |
|  |  |  |  | leucyl/cystinyl aminopeptidase (LNPEP), transcript variant 1 | 3 |
|  |  |  |  | guanylate cyclase 2F, retinal (GUCY2F) | 3 |
|  |  |  |  | syntrophin, gamma 2 (SNTG2) | 3 |
|  |  |  |  | WD repeat and FYVE domain containing 2 (WDFY2) | 3 |
|  |  |  |  | interferon-induced protein with tetratricopeptide repeats 5 (IFIT5) | 3 |
|  |  |  |  | nuclear factor, interleukin 3 regulated (NFIL3) | 3 |
|  |  |  |  | opioid growth factor receptor (OGFR) | 3 |
|  |  |  |  | BCL2-like 10 (apoptosis facilitator) (BCL2L10) | 3 |
|  |  |  |  | protein phosphatase 1, regulatory (inhibitor) subunit 15A (PPP1R15A) | 3 |
|  |  |  |  | optineurin (OPTN), transcript variant 2 | 3 |
|  |  |  |  | flavin containing monooxygenase 3 (FMO3), transcript variant 1 | 3 |
|  |  |  |  | cDNA FLJ37853 fis, clone BRSSN2014573 | 3 |
|  |  |  |  | cAMP responsive element modulator (CREM), transcript variant 9 | 3 |
|  |  |  |  | myeloid cell leukemia sequence 1 (BCL2-related), transcript variant 1, mRNA (cDNA clone MGC:88576 IMAGE:6586159) | 3 |
|  |  |  |  | potassium voltage-gated channel, Shaw-related subfamily, member 1 (KCNC1) | 3 |
|  |  |  |  | lipoyltransferase 1 (LIPT1), transcript variant 1 | 3 |
|  |  |  |  | collagen, type VIII, alpha 2 (COL8A2) | 3 |
|  |  |  |  | Rhesus blood group-associated glycoprotein (RHAG) | 3 |
|  |  |  |  | Kruppel-like factor 4 (gut) (KLF4) | 3 |
|  |  |  |  | follistatin (FST), transcript variant FST317 | 3 |
|  |  |  |  | DEAD (Asp-Glu-Ala-Asp) box polypeptide 39 (DDX39), transcript variant 1 | 3 |
|  |  |  |  | glucose-6-phosphate dehydrogenase | 3 |
|  |  |  |  | transmembrane protease, serine 12 (TMPRSS12) | 3 |
|  |  |  |  | D-aspartate oxidase (DDO), transcript variant 2 | 3 |
|  |  |  |  | docking protein 5 (DOK5), transcript variant 1 | 3 |
|  |  |  |  | IL3-CT0220-150200-068-F02 CT0220 Homo sapiens cDNA | 3 |
|  |  |  |  | ES cell expressed Ras (ERAS) | 3 |
|  |  |  |  | prostaglandin-endoperoxide synthase 2 (prostaglandin G/H synthase and cyclooxygenase) (PTGS2) | 3 |
|  |  |  |  | DIRAS family, GTP-binding RAS-like 3 (DIRAS3) | 3 |
|  |  |  |  | tumor protein p53 inducible nuclear protein 2 (TP53INP2) | 3 |
|  |  |  |  | RNA binding motif, single stranded interacting protein 2 (RBMS2) | 3 |
|  |  |  |  | CASP8 and FADD-like apoptosis regulator (CFLAR) | 3 |
|  |  |  |  | dynein, axonemal, heavy polypeptide 7 (DNAH7) | 3 |
|  |  |  |  | fms-related tyrosine kinase 4 (FLT4), transcript variant 2 | 3 |
|  |  |  |  | zinc finger protein 313 (ZNF313) | 3 |
|  |  |  |  | activating transcription factor 7 interacting protein 2 (ATF7IP2) | 3 |
|  |  |  |  | p21 (CDKN1A)-activated kinase 3 (PAK3) | 3 |
|  |  |  |  | pre-B-cell colony enhancing factor 1 (PBEF1), transcript variant 1 | 3 |
|  |  |  |  | frizzled homolog 10 (Drosophila) (FZD10) | 3 |
|  |  |  |  | interleukin 3 receptor, alpha (low affinity) (IL3RA) | 3 |
|  |  |  |  | platelet-derived growth factor receptor-like (PDGFRL) | 3 |
|  |  |  |  | zinc finger, CCHC domain containing 2 (ZCCHC2) | 3 |
|  |  |  |  | CD3D antigen, delta polypeptide (TiT3 complex) (CD3D) | 3 |
|  |  |  |  | folate receptor 2 (fetal) (FOLR2) | 3 |
|  |  |  |  | hypothetical protein FLJ36046 (FLJ36046) | 3 |
|  |  |  |  | matrilin 4 (MATN4), transcript variant 1 | 3 |
|  |  |  |  | PHD finger protein 5A (PHF5A) | 3 |
|  |  |  |  | cullin 3 (CUL3) | 3 |
|  |  |  |  | promyelocytic leukemia (PML), transcript variant 1 | 3 |
|  |  |  |  | GTP binding protein 2 (GTPBP2) | 3 |
|  |  |  |  | enhancer of zeste homolog 1 (Drosophila) (EZH1) | 3 |
|  |  |  |  | potassium voltage-gated channel, Shal-related subfamily, member 2 (KCND2) | 3 |
|  |  |  |  | lysozyme-like 1 (LYZL1) | 3 |
|  |  |  |  | purinergic receptor P2Y, G-protein coupled, 5 (P2RY5) | 3 |
|  |  |  |  | CD40 antigen (TNF receptor superfamily member 5) (CD40), transcript variant 1 | 3 |
|  |  |  |  | PDZ domain containing 3 (PDZK3), transcript variant 2 | 3 |
|  |  |  |  | zinc finger protein 433 (ZNF433) | 3 |
|  |  |  |  | adenosine deaminase, RNA-specific (ADAR), transcript variant 3 | 3 |
|  |  |  |  | midkine (neurite growth-promoting factor 2) (MDK), transcript variant 3 | 3 |
|  |  |  |  | carbonic anhydrase VIII (CA8) | 3 |
|  |  |  |  | serine (or cysteine) proteinase inhibitor, clade A (alpha-1 antiproteinase, antitrypsin), member 4 (SERPINA4) | 3 |
|  |  |  |  | placenta-specific 4 (PLAC4) | 3 |
|  |  |  |  | interferon induced transmembrane protein 2 (1-8D) (IFITM2) | 3 |
|  |  |  |  | folate receptor 1 (adult) (FOLR1), transcript variant 2 | 3 |
|  |  |  |  | zinc finger protein 85 (HPF4, HTF1) (ZNF85) | 3 |
|  |  |  |  | ceruloplasmin (ferroxidase) (CP) | 3 |
|  |  |  |  | retinoic acid receptor responder (tazarotene induced) 1 (RARRES1), transcript variant 1 | 3 |
|  |  |  |  | glycerophosphodiester phosphodiesterase domain containing 4 (GDPD4) | 3 |
|  |  |  |  | serine/threonine kinase 17b (apoptosis-inducing) (STK17B) | 3 |
|  |  |  |  | helicase (DNA) B (HELB) | 3 |
|  |  |  |  | likely ortholog of mouse D11lgp2 (LGP2) | 3 |
|  |  |  |  | enamelin (ENAM) | 3 |
|  |  |  |  | carbohydrate (chondroitin 4) sulfotransferase 11 (CHST11) | 3 |
|  |  |  |  | zinc finger protein 37 homolog (mouse) (ZFP37) | 3 |
|  |  |  |  | GATA binding protein 3 (GATA3), transcript variant 1 | 3 |
|  |  |  |  | serine proteinase inhibitor (P19) mRNA | 3 |
|  |  |  |  | TAP binding protein (tapasin) (TAPBP), transcript variant 1 | 3 |
|  |  |  |  | methylthioadenosine phosphorylase (MTAP) mRNA | 3 |
|  |  |  |  | A kinase (PRKA) anchor protein 11 (AKAP11), transcript variant 2 | 3 |
|  |  |  |  | Ras homolog enriched in brain like 1 (RHEBL1) | 3 |
|  |  |  |  | ribosome binding protein 1 homolog 180kDa (dog) (RRBP1) | 3 |
|  |  |  |  | similar to RIKEN cDNA 1700016G05 (LOC136242) | 3 |
|  |  |  |  | HSPC072 protein (HSPC072) | 3 |
|  |  |  |  | inhibitor of kappa light polypeptide gene enhancer in B-cells, kinase epsilon (IKBKE) | 3 |
|  |  |  |  | karyopherin alpha 4 (importin alpha 3) (KPNA4) | 3 |
|  |  |  |  | agouti related protein homolog (mouse) (AGRP), transcript variant 1 | 3 |
|  |  |  |  | RAS guanyl releasing protein 3 (calcium and DAG-regulated) (RASGRP3) | 3 |
|  |  |  |  | TBC1 (tre-2/USP6, BUB2, cdc16) domain family, member 1, mRNA (cDNA clone IMAGE:5211948), with apparent retained intron | 3 |
|  |  |  |  | BCL2-related protein A1 (BCL2A1) | 3 |
|  |  |  |  | gasdermin domain containing 1 (GSDMDC1) | 3 |
|  |  |  |  | t-complex 10 (mouse) (TCP10) | 3 |
|  |  |  |  | synaptotagmin XIII (SYT13) | 3 |
|  |  |  |  | sulfide quinone reductase-like (yeast) (SQRDL) | 3 |
|  |  |  |  | hepatocyte nuclear factor 4, gamma (HNF4G) | 3 |
|  |  |  |  | G protein-coupled receptor 34 (GPR34) | 3 |
|  |  |  |  | RelA associated inhibitor (RAI) mRNA | 3 |
|  |  |  |  | serine (or cysteine) proteinase inhibitor, clade I (pancpin), member 2 (SERPINI2), transcript variant 2 | 3 |
|  |  |  |  | Rhesus blood group, C glycoprotein (RHCG) | 3 |
|  |  |  |  | claudin 4 (CLDN4) | 3 |
|  |  |  |  | UDP-N-acetyl-alpha-D-galactosamine:polypeptide N-acetylgalactosaminyltransferase 4 (GalNAc-T4) (GALNT4) | 3 |
|  |  |  |  | torsin family 1, member B (torsin B) (TOR1B) | 3 |
|  |  |  |  | AXIN1 up-regulated 1 (AXUD1) | 3 |
|  |  |  |  | insulin-like growth factor binding protein 1 (IGFBP1), transcript variant 1 | 3 |
|  |  |  |  | pregnancy specific beta-1-glycoprotein 11 (PSG11), transcript variant 1 | 3 |
|  |  |  |  | C-type lectin domain family 2, member B (CLEC2B) | 3 |
|  |  |  |  | protocadherin 20 (PCDH20) | 3 |
|  |  |  |  | KM-HN-1 protein (KM-HN-1) | 3 |
|  |  |  |  | Ras and Rab interactor 2 (RIN2) | 3 |
|  |  |  |  | proliferating cell nuclear antigen (PCNA), transcript variant 1 | 3 |
|  |  |  |  | MyoD family inhibitor (MDFI) | 3 |
|  |  |  |  | plasminogen activator, urokinase (PLAU) | 3 |
|  |  |  |  | BCL2-like 14 (apoptosis facilitator) (BCL2L14), transcript variant 2 | 3 |
|  |  |  |  | olfactory receptor, family 52, subfamily B, member 4 (OR52B4) | 3 |
|  |  |  |  | lysyl-tRNA synthetase (KARS) | 3 |
|  |  |  |  | SWI/SNF related, matrix associated, actin dependent regulator of chromatin, subfamily a, member 5 (SMARCA5) | 3 |
|  |  |  |  | angiopoietin-like 1 (ANGPTL1) | 3 |
|  |  |  |  | Cas-Br-M (murine) ecotropic retroviral transforming sequence c (CBLC) | 3 |
|  |  |  |  | thioredoxin domain containing (TXNDC) | 3 |
|  |  |  |  | Fas (TNF receptor superfamily, member 6) (FAS), transcript variant 1 | 3 |
|  |  |  |  | apolipoprotein A-V (APOA5) | 3 |
|  |  |  |  | diencephalon/mesencephalon homeobox 1 (DMBX1), transcript variant 1 | 3 |
|  |  |  |  | zinc finger protein 15-like 1 (KOX 8) (ZNF15L1) | 3 |
|  |  |  |  | WD repeat domain 49 (WDR49) | 3 |
|  |  |  |  | zinc finger protein 36, C3H type, homolog (mouse) (ZFP36) | 3 |
|  |  |  |  | CD38 antigen (p45) (CD38) | 3 |
|  |  |  |  | melanoma antigen family A, 10 (MAGEA10), transcript variant 2 | 3 |
|  |  |  |  | Down syndrome critical region gene 1 (DSCR1), transcript variant 3 | 3 |
|  |  |  |  | collagen, type XXIV, alpha 1 (COL24A1) | 3 |
|  |  |  |  | Ras-induced senescence 1 (RIS1) | 2 |
|  |  |  |  | lymphocyte transmembrane adaptor 1 (LAX1) | 2 |
|  |  |  |  | proteasome (prosome, macropain) activator subunit 1 (PA28 alpha) (PSME1), transcript variant 1 | 2 |
|  |  |  |  | hydroxysteroid (17-beta) dehydrogenase 1 (HSD17B1) | 2 |
|  |  |  |  | polycomb group ring finger 5 (PCGF5) | 2 |
|  |  |  |  | chordin-like 2 (CHRDL2) | 2 |
|  |  |  |  | elaC homolog 1 (E coli) (ELAC1) | 2 |
|  |  |  |  | distal-less homeo box 5 (DLX5) | 2 |
|  |  |  |  | LIM domain only 2 (rhombotin-like 1) (LMO2) | 2 |
|  |  |  |  | zinc finger and BTB domain containing 20 (ZBTB20) | 2 |
|  |  |  |  | heat shock 70kDa protein 5 (glucose-regulated protein, 78kDa) (HSPA5) | 2 |
|  |  |  |  | TCR gamma alternate reading frame protein (TARP), nuclear gene encoding mitochondrial protein, transcript variant 2 | 2 |
|  |  |  |  | sema domain, immunoglobulin domain (Ig), transmembrane domain (TM) and short cytoplasmic domain, (semaphorin) 4B (SEMA4B), transcript variant 1 | 2 |
|  |  |  |  | calpain 13 (CAPN13) | 2 |
|  |  |  |  | alcohol dehydrogenase 4 (class II), pi polypeptide (ADH4), mRNA | 2 |
|  |  |  |  | secretogranin II (chromogranin C) (SCG2) | 2 |
|  |  |  |  | CD14 antigen (CD14) | 2 |
|  |  |  |  | protocadherin 1 (cadherin-like 1) (PCDH1), transcript variant 1 | 2 |
|  |  |  |  | oncostatin M receptor (OSMR) | 2 |
|  |  |  |  | complement component 1, q subcomponent-like 1 (C1QL1) | 2 |
|  |  |  |  | syndecan 4 (amphiglycan, ryudocan) (SDC4) | 2 |
|  |  |  |  | coagulation factor III (thromboplastin, tissue factor) (F3) | 2 |
|  |  |  |  | multimerin 1 (MMRN1) | 2 |
|  |  |  |  | alpha-2,8-polysialyltransferase (PST) gene | 2 |
|  |  |  |  | RAB3A interacting protein (rabin3) (RAB3IP), transcript variant alpha 1 | 2 |
|  |  |  |  | secretogranin III (SCG3) | 2 |
|  |  |  |  | leptin (obesity homolog, mouse) (LEP) | 2 |
|  |  |  |  | huntingtin interacting protein-1-related (HIP1R) | 2 |
|  |  |  |  | hepatitis A virus cellular receptor 1 (HAVCR1) | 2 |
|  |  |  |  | ficolin (collagen/fibrinogen domain containing lectin) 2 (hucolin) (FCN2), transcript variant SV1 | 2 |
|  |  |  |  | zinc finger CCCH-type containing 12A (ZC3H12A) | 2 |
|  |  |  |  | spermatogenesis associated 1 (SPATA1) | 2 |
|  |  |  |  | tribbles homolog 1 (Drosophila) (TRIB1) | 2 |
|  |  |  |  | Janus kinase 2 (a protein tyrosine kinase) (JAK2) | 2 |
|  |  |  |  | zinc finger protein 253 (ZNF253) | 2 |
|  |  |  |  | CUB and Sushi multiple domains 1 (CSMD1) | 2 |
|  |  |  |  | peptidylprolyl isomerase (cyclophilin)-like 2 (PPIL2), transcript variant 1 | 2 |
|  |  |  |  | guanylate binding protein 5 (GBP5) | 2 |
|  |  |  |  | golgi associated, gamma adaptin ear containing, ARF binding protein 3 (GGA3), transcript variant short | 2 |
|  |  |  |  | fibronectin type III domain containing 3A (FNDC3A) | 2 |
|  |  |  |  | BCL2 binding component 3 (BBC3) | 2 |
|  |  |  |  | signal transducer and activator of transcription 5A (STAT5A) | 2 |
|  |  |  |  | toll-like receptor adaptor molecule 1 (TICAM1), transcript variant 2 | 2 |
|  |  |  |  | collagen, type IV, alpha 1 (COL4A1) | 2 |
|  |  |  |  | TORC2-specific protein AVO3 (AVO3) | 2 |
|  |  |  |  | musculin (activated B-cell factor-1) (MSC) | 2 |
|  |  |  |  | replication factor C (activator 1) 3, 38kDa (RFC3), transcript variant 1 | 2 |
|  |  |  |  | TPA-induced transmembrane protein (TTMP) | 2 |
|  |  |  |  | B-cell scaffold protein with ankyrin repeats 1 (BANK1) | 2 |
|  |  |  |  | NACHT, leucine rich repeat and PYD containing 10 (NALP10) | 2 |
|  |  |  |  | actin-related protein T1 (RP1-203C21) | 2 |
|  |  |  |  | insulin-like growth factor binding protein 1 (IGFBP1), transcript variant 1 | 2 |
|  |  |  |  | protease, serine, 8 (prostasin) (PRSS8) | 2 |
|  |  |  |  | basic helix-loop-helix domain containing, class B, 3 (BHLHB3) | 2 |
|  |  |  |  | vomeronasal 1 receptor 5 (VN1R5) | 2 |
|  |  |  |  | kin of IRRE like 2 (Drosophila) (KIRREL2), transcript variant 3 | 2 |
|  |  |  |  | meprin A, beta (MEP1B) | 2 |
|  |  |  |  | 28kD interferon responsive protein (IFRG28) | 2 |
|  |  |  |  | opioid receptor, delta 1 (OPRD1) | 2 |
|  |  |  |  | keratin, hair, acidic, 4 (KRTHA4) | 2 |
|  |  |  |  | cleavage stimulation factor, 3' pre-RNA, subunit 3, 77kDa (CSTF3) | 2 |
|  |  |  |  | WNT1 inducible signaling pathway protein 3 (WISP3), transcript variant 1 | 2 |
|  |  |  |  | nuclear receptor co-repressor 2 (NCOR2) | 2 |
|  |  |  |  | SH3-domain GRB2-like endophilin B1 (SH3GLB1) | 2 |
|  |  |  |  | NUAK family, SNF1-like kinase, 2 (NUAK2) | 2 |
|  |  |  |  | G protein-coupled receptor 22 (GPR22) | 2 |
|  |  |  |  | transmembrane protein 47 (TMEM47) | 2 |
|  |  |  |  | cartilage acidic protein 1 (CRTAC1) | 2 |
|  |  |  |  | AU RNA binding protein/enoyl-Coenzyme A hydratase (AUH), nuclear gene encoding mitochondrial protein | 2 |
|  |  |  |  | zinc finger protein 175 (ZNF175) | 2 |
|  |  |  |  | growth factor independent 1 (GFI1) | 2 |
|  |  |  |  | H2A histone family, member J (H2AFJ), transcript variant 1 | 2 |
|  |  |  |  | ADAMTS-like 3 (ADAMTSL3) | 2 |
|  |  |  |  | keratin associated protein 13-1 (KRTAP13-1) | 2 |
|  |  |  |  | serine (or cysteine) proteinase inhibitor, clade B (ovalbumin), member 8 (SERPINB8), transcript variant 2 | 2 |
|  |  |  |  | sarcoma antigen 1 (SAGE1) | 2 |
|  |  |  |  | GATA binding protein 6 (GATA6) | 2 |
|  |  |  |  | thyroid hormone receptor interactor 12 (TRIP12) | 2 |
|  |  |  |  | Rho family GTPase 1 (RND1) | 2 |
|  |  |  |  | Rap guanine nucleotide exchange factor (GEF) 5 (RAPGEF5) | 2 |
|  |  |  |  | RalBP1 associated Eps domain containing protein 2 variant protein | 2 |
|  |  |  |  | msh homeo box homolog 1 (Drosophila) (MSX1) | 2 |
|  |  |  |  | thioredoxin domain containing 8 (TXNDC8) | 2 |
|  |  |  |  | pappalysin 2 (PAPPA2), transcript variant 2 | 2 |
|  |  |  |  | P450 (cytochrome) oxidoreductase (POR) | 2 |
|  |  |  |  | napsin B aspartic peptidase pseudogene, mRNA (cDNA clone IMAGE:5209672) | 2 |
|  |  |  |  | proline rich protein 5 (PRR5), transcript variant 2 | 2 |
|  |  |  |  | leucine zipper transcription regulator 2 (LZTR2) | 2 |
|  |  |  |  | retinitis pigmentosa 2 (X-linked recessive) (RP2) | 2 |
|  |  |  |  | promyelocytic leukemia (PML), transcript variant 1 | 2 |
|  |  |  |  | coagulation factor II (thrombin) receptor-like 1 (F2RL1) | 2 |
|  |  |  |  | guanylate cyclase 1, soluble, alpha 3 (GUCY1A3) | 2 |
|  |  |  |  | zinc finger homeobox 1b (ZFHX1B) | 2 |
|  |  |  |  | angiopoietin 1 (ANGPT1), transcript variant 1 | 2 |
|  |  |  |  | resistin like beta (RETNLB) | 2 |
|  |  |  |  | natural cytotoxicity triggering receptor 3 (NCR3) | 2 |
|  |  |  |  | fibrinogen alpha chain (FGA), transcript variant alpha-E | 2 |
|  |  |  |  | hippocampus abundant transcript 1 (HIAT1) | 2 |
|  |  |  |  | CCAAT/enhancer binding protein (C/EBP), beta (CEBPB) | 2 |
|  |  |  |  | tropomodulin 2 (neuronal) (TMOD2) | 2 |
|  |  |  |  | UDP-N-acetyl-alpha-D-galactosamine:polypeptide N-acetylgalactosaminyltransferase 14 (GalNAc-T14) (GALNT14) | 2 |
|  |  |  |  | epithelial protein lost in neoplasm beta (EPLIN) | 2 |
|  |  |  |  | guanine nucleotide binding protein-like 3 (nucleolar) (GNL3), transcript variant 3 | 2 |
|  |  |  |  | ring finger protein 31 (RNF31) | 2 |
|  |  |  |  | retinitis pigmentosa 1 (autosomal dominant) (RP1) | 2 |
|  |  |  |  | tripartite motif-containing 26 (TRIM26) | 2 |
|  |  |  |  | full length insert cDNA clone YU28F12 | 2 |
|  |  |  |  | sorting nexin 6 (SNX6), transcript variant 1 | 2 |
|  |  |  |  | TDP-glucose 4,6-dehydratase (TGDS) | 2 |
|  |  |  |  | hyaluronan synthase 3 (HAS3), transcript variant 1 | 2 |
|  |  |  |  | histone 1, H4h (HIST1H4H) | 2 |
|  |  |  |  | acid phosphatase 2, lysosomal (ACP2) | 2 |
|  |  |  |  | centaurin, alpha 1 (CENTA1) | 2 |
|  |  |  |  | otopetrin 3 (OTOP3) | 2 |
|  |  |  |  | carcinoembryonic antigen-related cell adhesion molecule (R29124_1) | 2 |
|  |  |  |  | v-fos FBJ murine osteosarcoma viral oncogene homolog (FOS) | 2 |
|  |  |  |  | yf74f04s1 Soares infant brain 1NIB cDNA clone IMAGE:27988 3' | 2 |
|  |  |  |  | zinc finger protein, subfamily 1A, 5 (ZNFN1A5) | 2 |
|  |  |  |  | serine/threonine kinase 17a (apoptosis-inducing) (STK17A) | 2 |
|  |  |  |  | TNF receptor-associated factor 3 (TRAF3), transcript variant 3 | 2 |
|  |  |  |  | calcium channel, voltage-dependent, gamma subunit 8 (CACNG8) | 2 |
|  |  |  |  | mucin 15 (MUC15) | 2 |
|  |  |  |  | pro-melanin-concentrating hormone (PMCH), mRNA | 2 |
|  |  |  |  | Rhesus blood group, D antigen (RHD), transcript variant 1 | 2 |
|  |  |  |  | heat shock 70kDa protein 5 (glucose-regulated protein, 78kDa) binding protein 1 (HSPA5BP1), transcript variant 1 | 2 |
|  |  |  |  | interferon, beta 1, fibroblast (IFNB1) | 2 |
|  |  |  |  | inositol hexaphosphate kinase 1 (IHPK1), transcript variant 1 | 2 |
|  |  |  |  | absent in melanoma 1 (AIM1) | 2 |
|  |  |  |  | lysyl oxidase-like 3 (LOXL3) | 2 |
|  |  |  |  | procollagen-proline, 2-oxoglutarate 4-dioxygenase (proline 4-hydroxylase), alpha polypeptide I (P4HA1), transcript variant 1 | 2 |
|  |  |  |  | 2,4-dienoyl CoA reductase 1, mitochondrial (DECR1), nuclear gene encoding mitochondrial protein | 2 |
|  |  |  |  | CCR4-NOT transcription complex, subunit 3 (CNOT3) | 2 |
|  |  |  |  | testicular soluble adenylyl cyclase (SAC) | 2 |
|  |  |  |  | RNA binding motif protein 15 (RBM15) | 2 |
|  |  |  |  | heparan sulfate 6-O-sulfotransferase 3 (HS6ST3) | 2 |
|  |  |  |  | aspartoacylase (aminocyclase) 3 (ACY3) | 2 |
|  |  |  |  | nuclear factor of kappa light polypeptide gene enhancer in B-cells 1 (p105) (NFKB1) | 2 |
|  |  |  |  | glutamine-fructose-6-phosphate transaminase 2 (GFPT2) | 2 |
|  |  |  |  | protein arginine N-methyltransferase 7 (PRMT7) | 2 |
|  |  |  |  | suppressor of variegation 3-9 homolog 2 (Drosophila) (SUV39H2) | 2 |
|  |  |  |  | zinc finger protein 274 (ZNF274), transcript variant ZNF274a | 2 |
|  |  |  |  | proline-rich nuclear receptor coactivator 1 (PNRC1) | 2 |
|  |  |  |  | leucine rich repeat neuronal 3 (LRRN3) | 2 |
|  |  |  |  | chromatin modifying protein 5 (CHMP5) | 2 |
|  |  |  |  | DnaJ (Hsp40) homolog, subfamily B, member 9 (DNAJB9) | 2 |
|  |  |  |  | sulfotransferase family, cytosolic, 1B, member 1 (SULT1B1) | 2 |
|  |  |  |  | poly (ADP-ribose) polymerase family, member 8 (PARP8) | 2 |
|  |  |  |  | complement component 2 (C2) | 2 |
|  |  |  |  | TGFB-induced factor 2-like, X-linked (TGIF2LX) | 2 |
|  |  |  |  | poly (ADP-ribose) polymerase family, member 3 (PARP3), transcript variant 2 | 2 |
|  |  |  |  | endothelin receptor type A (EDNRA) | 2 |
|  |  |  |  | kelch repeat and BTB (POZ) domain containing 10 (KBTBD10) | 2 |
|  |  |  |  | CD48 antigen (B-cell membrane protein) (CD48) | 2 |
|  |  |  |  | nipsnap homolog 3A (C elegans) (NIPSNAP3A) | 2 |
|  |  |  |  | UDP-glucose ceramide glucosyltransferase (UGCG) | 2 |
|  |  |  |  | amyloid beta (A4) precursor protein-binding, family B, member 2 (Fe65-like) (APBB2) | 2 |
|  |  |  |  | ubiquitin C (UBC) | 2 |
|  |  |  |  | glucose-fructose oxidoreductase domain containing 1 (GFOD1) | 2 |
|  |  |  |  | 5'-3' exoribonuclease 2 (XRN2) | 2 |
|  |  |  |  | argininosuccinate synthetase (ASS), transcript variant 1 | 2 |
|  |  |  |  | PDZ and LIM domain 3 (PDLIM3) | 2 |
|  |  |  |  | SCO cytochrome oxidase deficient homolog 2 (yeast) (SCO2), nuclear gene encoding mitochondrial protein | 2 |
|  |  |  |  | a disintegrin-like and metalloprotease (reprolysin type) with thrombospondin type 1 motif, 7 (ADAMTS7) | 2 |
|  |  |  |  | adipocyte-specific adhesion molecule (ASAM) | 2 |
|  |  |  |  | immunodeficiency virus type I enhancer binding protein 3 (HIVEP3) | 2 |
|  |  |  |  | ATPase, H+ transporting, lysosomal 56/58kDa, V1 subunit B, isoform 1 (Renal tubular acidosis with deafness) (ATP6V1B1) | 2 |
|  |  |  |  | histidine triad nucleotide binding protein 3 (HINT3) | 2 |
|  |  |  |  | replication initiator 1 (REPIN1), transcript variant 2 | 2 |
|  |  |  |  | DnaJ (Hsp40) homolog, subfamily A, member 2 (DNAJA2) | 2 |
|  |  |  |  | CD164 antigen, sialomucin (CD164) | 2 |
|  |  |  |  | PRO1768 protein (PRO1768), mRNA | 2 |
|  |  |  |  | inositol(myo)-1(or 4)-monophosphatase 1 (IMPA1) | 2 |
|  |  |  |  | histone 2, H2be (HIST2H2BE) | 2 |
|  |  |  |  | deoxyribonuclease I-like 3 (DNASE1L3) | 2 |
|  |  |  |  | mad protein homolog (hMAD-3) mRNA | 2 |
|  |  |  |  | olfactory receptor, family 8, subfamily B, member 8 (OR8B8) | 2 |
|  |  |  |  | BET1 homolog (S cerevisiae) (BET1) | 2 |
|  |  |  |  | DnaJ (Hsp40) homolog, subfamily B, member 6 (DNAJB6), transcript variant 2 | 2 |
|  |  |  |  | nuclear autoantigenic sperm protein (histone-binding) (NASP), transcript variant 2 | 2 |
|  |  |  |  | FGFR1 oncogene partner 2 (FGFR1OP2) | 2 |
|  |  |  |  | pellino homolog 1 (Drosophila) (PELI1) | 2 |
|  |  |  |  | neuron navigator 3 (NAV3) | 2 |
|  |  |  |  | nuclear factor of activated T-cells, cytoplasmic, calcineurin-dependent 4 (NFATC4) | 2 |
|  |  |  |  | WD repeat domain 48 (WDR48) | 2 |
|  |  |  |  | wm70b10x1 NCI_CGAP_Ut2 cDNA clone IMAGE:2441275 3' | 2 |
|  |  |  |  | a disintegrin-like and metalloprotease (reprolysin type) with thrombospondin type 1 motif, 6 (ADAMTS6) | 2 |
|  |  |  |  | protein disulfide isomerase family A, member 3 (PDIA3) | 2 |
|  |  |  |  | short coiled-coil protein (SCOC) | 2 |
|  |  |  |  | GRB2-related adaptor protein 2 (GRAP2) | 2 |
|  |  |  |  | ATP-binding cassette, sub-family A (ABC1), member 13 (ABCA13) | 2 |
|  |  |  |  | synaptotagmin VII mRNA | 2 |
|  |  |  |  | cytokine receptor-like factor 2 (CRLF2), transcript variant 1 | 2 |
|  |  |  |  | caldesmon 1 (CALD1), transcript variant 4 | 2 |
|  |  |  |  | carbohydrate (N-acetylglucosamine 6-O) sulfotransferase 4 (CHST4) | 2 |
|  |  |  |  | SH3-domain kinase binding protein 1 (SH3KBP1), transcript variant 1 | 2 |
|  |  |  |  | serologically defined colon cancer antigen 10 (SDCCAG10) | 2 |
|  |  |  |  | gonadotropin inducible transcription repressor 1 (GIOT-1) | 2 |
|  |  |  |  | v-ets erythroblastosis virus E26 oncogene homolog 2 (avian) (ETS2) | 2 |
|  |  |  |  | lectin, galactoside-binding, soluble, 8 (galectin 8) (LGALS8), transcript variant 3 | 2 |
|  |  |  |  | aldolase pseudogene mRNA | 2 |
|  |  |  |  | Sec23 homolog B (S cerevisiae) (SEC23B), transcript variant 3 | 2 |
|  |  |  |  | nuclear factor I/X (CCAAT-binding transcription factor) (NFIX) | 2 |
|  |  |  |  | regulator of G-protein signalling 16 (RGS16) | 2 |
|  |  |  |  | sterile alpha motif domain containing 3 (SAMD3), transcript variant 2 | 2 |
|  |  |  |  | ring finger protein 138 (RNF138), transcript variant 2 | 2 |
|  |  |  |  | trophoblast glycoprotein (TPBG) | 2 |
|  |  |  |  | pre-T/NK cell associated protein (6H9A), mRNA | 2 |
|  |  |  |  | protocadherin beta 14 (PCDHB14) | 2 |
|  |  |  |  | protein disulfide isomerase family A, member 5 (PDIA5) | 2 |
|  |  |  |  | CDC42 effector protein (Rho GTPase binding) 2 (CDC42EP2) | 2 |
|  |  |  |  | zinc finger protein 36, C3H type-like 2 (ZFP36L2) | 2 |
|  |  |  |  | creatine kinase, mitochondrial 2 (sarcomeric) (CKMT2), nuclear gene encoding mitochondrial protein | 2 |
|  |  |  |  | jun B proto-oncogene (JUNB) | 2 |
|  |  |  |  | chaperonin containing TCP1, subunit 8 (theta) (CCT8) | 2 |
|  |  |  |  | cysteine-rich secretory protein LCCL domain containing 1 (CRISPLD1) | 2 |
|  |  |  |  | ADP-ribosylhydrolase like 2 (ADPRHL2) | 2 |
|  |  |  |  | suppressor of S cerevisiae gcr2 (HSGT1) | 2 |
|  |  |  |  | dehydrogenase/reductase (SDR family) member 9 (DHRS9), transcript variant 2 | 2 |
|  |  |  |  | FXYD domain containing ion transport regulator 1 (phospholemman) (FXYD1), transcript variant b | 2 |
|  |  |  |  | potassium voltage-gated channel, KQT-like subfamily, member 2 (KCNQ2), transcript variant 3 | 2 |
|  |  |  |  | La ribonucleoprotein domain family, member 6 (LARP6), transcript variant 1 | 2 |
|  |  |  |  | serine carboxypeptidase 1 (SCPEP1) | 2 |
|  |  |  |  | cathepsin S (CTSS) | 2 |
|  |  |  |  | sex comb on midleg-like 4 (Drosophila) (SCML4) | 2 |
|  |  |  |  | immunodeficiency virus type I enhancer binding protein 2 (HIVEP2) | 2 |
|  |  |  |  | BCL2-interacting killer (apoptosis-inducing) (BIK) | 2 |
|  |  |  |  | zinc finger protein 44 (KOX 7) (ZNF44) | 2 |
|  |  |  |  | hypothetical protein FLJ31951 (FLJ31951) | 2 |
|  |  |  |  | leukocyte specific transcript 1 (LST1), transcript variant 1 | 2 |
|  |  |  |  | BTG family, member 3 (BTG3) | 2 |
|  |  |  |  | growth arrest-specific 7, mRNA (cDNA clone MGC:1347 IMAGE:3353809) | 2 |
|  |  |  |  | heat shock protein, alpha-crystallin-related, B6 (HSPB6) | 2 |
|  |  |  |  | hypothetical protein PRO2176 (PRO2176), mRNA | 2 |
|  |  |  |  | upstream transcription factor 1 (USF1), transcript variant 1 | 2 |
|  |  |  |  | vav 1 oncogene (VAV1) | 2 |
|  |  |  |  | nuclear receptor subfamily 2, group F, member 2 (NR2F2) | 2 |
|  |  |  |  | zinc finger protein 638 (ZNF638), transcript variant 1 | 2 |
|  |  |  |  | HMG-box transcription factor 1 (HBP1) | 2 |
|  |  |  |  | coagulation factor II (thrombin) receptor-like 1 (F2RL1) | 2 |
|  |  |  |  | immediate early response 5 (IER5) | 2 |
|  |  |  |  | fibroblast growth factor receptor 2 (bacteria-expressed kinase, keratinocyte growth factor receptor, craniofacial dysostosis 1, Crouzon syndrome, Pfeiffer syndrome, Jackson-Weiss syndrome) (FGFR2), transcript variant 13 | 2 |
|  |  |  |  | 5'-3' exoribonuclease 1 (XRN1) | 2 |
|  |  |  |  | pyruvate dehydrogenase kinase, isoenzyme 4 (PDK4) | 2 |
|  |  |  |  | hydroxysteroid (17-beta) dehydrogenase 6 (HSD17B6) | 2 |
|  |  |  |  | a disintegrin and metalloproteinase domain 17 (tumor necrosis factor, alpha, converting enzyme) (ADAM17), transcript variant 1 | 2 |
|  |  |  |  | polycystic kidney disease 2-like 2 (PKD2L2) | 2 |
|  |  |  |  | olfactory receptor, family 1, subfamily L, member 3 (OR1L3) | 2 |
|  |  |  |  | CD47 antigen (Rh-related antigen, integrin-associated signal transducer) (CD47), transcript variant 1 | 2 |
|  |  |  |  | suppressor of cytokine signaling 3 (SOCS3) | 2 |
|  |  |  |  | step II splicing factor SLU7 (SLU7) | 2 |
|  |  |  |  | desert hedgehog homolog (Drosophila) (DHH) | 2 |
|  |  |  |  | dihydropyrimidine dehydrogenase (DPYD) | 2 |
|  |  |  |  | RAB35, member RAS oncogene family (RAB35) | 2 |
|  |  |  |  | arachidonate 15-lipoxygenase (ALOX15) | 2 |
|  |  |  |  | phosphatidic acid phosphatase type 2B (PPAP2B), transcript variant 1 | 2 |
|  |  |  |  | sine oculis homeobox homolog 1 (Drosophila) (SIX1) | 2 |
|  |  |  |  | fatty acid 2-hydroxylase (FA2H) | 2 |
|  |  |  |  | twisted gastrulation homolog 1 (Drosophila) (TWSG1) | 2 |
|  |  |  |  | proteasome (prosome, macropain) 26S subunit, non-ATPase, 14 (PSMD14) | 2 |
|  |  |  |  | scratch homolog 2, zinc finger protein (Drosophila) (SCRT2) | 2 |
|  |  |  |  | nuclear cap binding protein subunit 1, 80kDa (NCBP1) | 2 |
|  |  |  |  | HBS1-like (S cerevisiae) (HBS1L) | 2 |
|  |  |  |  | cysteine-rich motor neuron 1 (CRIM1) | 2 |
|  |  |  |  | bromodomain adjacent to zinc finger domain, 1A (BAZ1A), transcript variant 1 | 2 |
|  |  |  |  | similar to transketolase (DKFZP434L1717) | 2 |
|  |  |  |  | taste receptor, type 2, member 49 (TAS2R49) | 2 |
|  |  |  |  | putative nucleic acid binding protein RY-1 (RY1) | 2 |
|  |  |  |  | RAB9A, member RAS oncogene family (RAB9A) | 2 |
|  |  |  |  | Kruppel-like factor 6 (KLF6), transcript variant 2 | 2 |
|  |  |  |  | pleckstrin homology domain containing, family A (phosphoinositide binding specific) member 3 (PLEKHA3) | 2 |
|  |  |  |  | zi66d04s1 Soares_fetal_liver_spleen_1NFLS_S1 cDNA clone IMAGE:435751 3' | 2 |
|  |  |  |  | catenin, beta like 1 (CTNNBL1) | 2 |
|  |  |  |  | 4-aminobutyrate aminotransferase (GABAT) mRNA | 2 |
|  |  |  |  | DNA polymerase-transactivated protein 6 (DNAPTP6) | 2 |
|  |  |  |  | protein phosphatase 2 (formerly 2A), catalytic subunit, alpha isoform (PPP2CA) | 2 |
|  |  |  |  | potassium channel tetramerisation domain containing 16 (KCTD16) | 2 |
|  |  |  |  | splicing factor proline/glutamine rich (polypyrimidine tract binding protein associated) (SFPQ) | 2 |
|  |  |  |  | attractin (ATRN), transcript variant 1 | 2 |
|  |  |  |  | PHD finger protein 7 (PHF7), transcript variant 1 | 2 |
|  |  |  |  | signal transducer and activator of transcription 3 (acute-phase response factor) (STAT3), transcript variant 2 | 2 |
|  |  |  |  | prokineticin 1 (PROK1) | 2 |
|  |  |  |  | serine/threonine kinase 24 (STE20 homolog, yeast) (STK24) | 2 |
|  |  |  |  | DnaJ (Hsp40) homolog, subfamily B, member 11 (DNAJB11) | 2 |
|  |  |  |  | F-box protein 11 (FBXO11), transcript variant 1 | 2 |
|  |  |  |  | vomeronasal 1 receptor 2 (VN1R2) | 2 |
|  |  |  |  | retinoic acid induced 14 (RAI14) | 2 |
|  |  |  |  | major histocompatibility complex, class II, DP beta 1 (HLA-DPB1) | 2 |
|  |  |  |  | netrin G2 (NTNG2) | 2 |
|  |  |  |  | glutamate receptor, metabotropic 7 (GRM7), transcript variant 1 | 2 |
|  |  |  |  | 5-azacytidine induced 2 (AZI2) | 2 |
|  |  |  |  | mal, T-cell differentiation protein (MAL), transcript variant d | 2 |
|  |  |  |  | phosphatidylinositol-4-phosphate 5-kinase, type I, beta (PIP5K1B) | 2 |
|  |  |  |  | very low density lipoprotein receptor (VLDLR), transcript variant 1 | 2 |
|  |  |  |  | glutamate receptor, ionotropic, N-methyl D-asparate-associated protein 1 (glutamate binding) (GRINA), transcript variant 1 | 2 |
|  |  |  |  | MICAL-like 1 (MICAL-L1) | 2 |
|  |  |  |  | proline-serine-threonine phosphatase interacting protein 2 (PSTPIP2) | 2 |
|  |  |  |  | neurofilament, heavy polypeptide 200kDa (NEFH) | 2 |
|  |  |  |  | haptoglobin (HP) | 2 |
|  |  |  |  | six transmembrane epithelial antigen of the prostate 1 (STEAP1) | 2 |
|  |  |  |  | zinc finger protein 342 (ZNF342) | 2 |
|  |  |  |  | sodium channel, voltage-gated, type II, beta (SCN2B) | 2 |
|  |  |  |  | tripartite motif-containing 31 (TRIM31), transcript variant 1 | 2 |
|  |  |  |  | Bardet-Biedl syndrome 5 (BBS5) | 2 |
|  |  |  |  | myosin IB (MYO1B) | 2 |
|  |  |  |  | tubulin, delta 1 (TUBD1) | 2 |
|  |  |  |  | RNA binding protein with multiple splicing 2 (RBPMS2) | 2 |
|  |  |  |  | kinesin 2 60/70kDa (KNS2), transcript variant 1 | 2 |
|  |  |  |  | serine/threonine kinase 3 (STE20 homolog, yeast) (STK3) | 2 |
|  |  |  |  | src family associated phosphoprotein 1 (SCAP1) | 2 |
|  |  |  |  | interferon gamma receptor 2 (interferon gamma transducer 1) (IFNGR2) | 2 |
|  |  |  |  | fibronectin type III domain containing 3B (FNDC3B) | 2 |
|  |  |  |  | calcium channel, voltage-dependent, L type, alpha 1S subunit (CACNA1S) | 2 |
|  |  |  |  | claudin 2 (CLDN2) | 2 |
|  |  |  |  | similar to autoantigen La (LOC352252), mRNA | 2 |
|  |  |  |  | interleukin 13 (IL13) | 2 |
|  |  |  |  | amyotrophic lateral sclerosis 2 (juvenile) chromosome region, candidate 11 (ALS2CR11) | 2 |
|  |  |  |  | growth differentiation factor 8 (GDF8) | 2 |
|  |  |  |  | HOA10-1-C11 HOA (Human Osteoarthritic Cartilage) Homo sapiens cDNA | 2 |
|  |  |  |  | synaptic vesicle glycoprotein 2B (SV2B) | 2 |
|  |  |  |  | sushi domain containing 3 (SUSD3) | 2 |
|  |  |  |  | actin related protein 2/3 complex, subunit 4, 20kDa (ARPC4), transcript variant 1 | 2 |
|  |  |  |  | sorting nexin 4 (SNX4) | 2 |
|  |  |  |  | secretory carrier membrane protein 1 (SCAMP1), transcript variant 1 | 2 |
|  |  |  |  | betacellulin (BTC) | 2 |
|  |  |  |  | L-glycerol-3-phosphate:NAD oxidoreductase mRNA | -2 |
|  |  |  |  | p65 protein | -2 |
|  |  |  |  | albumin (ALB) | -2 |
|  |  |  |  | homeo box C12 (HOXC12) | -3 |
|  |  |  |  | Fer3-like (Drosophila) (FERD3L) | -3 |
|  |  |  |  | zinc finger protein 610 (ZNF610) | -3 |
|  |  |  |  | IL3-CT0674-060401-492-F11 CT0674 Homo sapiens cDNA, mRNA sequence | -3 |
|  |  |  |  | Sp6 transcription factor (SP6) | -3 |
|  |  |  |  | DEAH (Asp-Glu-Ala-Asp/His) box polypeptide 57 (DHX57), transcript variant 3 | -4 |
|  |  |  |  | TU3A protein (TU3A) | -5 |
|  |  |  |  | a disintegrin-like and metalloprotease (reprolysin type) with thrombospondin type 1 motif, 20 (ADAMTS20), transcript variant 1 | -6 |
|  |  |  |  | RGM domain family, member A (RGMA) | -6 |
|  |  |  |  | interleukin 9 (IL9) | -6 |
|  |  |  |  | chordin-like 1 (CHRDL1) | -7 |
|  |  |  |  | nuclear receptor subfamily 5, group A, member 1 (NR5A1) | -7 |
|  |  |  |  | similar to Ab2-183 (LOC158830) | -8 |
|  |  |  |  | zinc finger protein 560 (ZNF560) | -8 |
|  |  |  |  | vasoactive intestinal peptide receptor 2 (VIPR2) | -9 |
|  |  |  |  | dopachrome tautomerase (dopachrome delta-isomerase, tyrosine-related protein 2) (DCT) | -9 |
|  |  |  |  | casein alpha s1 (CSN1S1), transcript variant 1 | -9 |
|  |  |  |  | NK2 transcription factor related, locus 2 (Drosophila) (NKX2-2) | -10 |
|  |  |  |  | protocadherin alpha 2 (PCDHA2), transcript variant 2 | -10 |
|  |  |  |  | membrane-spanning 4-domains, subfamily A, member 6A (MS4A6A), transcript variant 2 | -10 |
|  |  |  |  | low density lipoprotein-related protein 1B (deleted in tumors) (LRP1B) | -10 |
|  |  |  |  | integrin, beta 6 (ITGB6) | -11 |
|  |  |  |  | inter-alpha (globulin) inhibitor H4 (plasma Kallikrein-sensitive glycoprotein) (ITIH4) | -12 |
|  |  |  |  | lipase, member I (LIPI) | -12 |
|  |  |  |  | distal-less homeo box 3 (DLX3) | -12 |
|  |  |  |  | prokineticin 2 (PROK2) | -12 |
|  |  |  |  | microseminoprotein, beta- (MSMB), transcript variant PSP57 | -14 |
|  |  |  |  | kallikrein 5 (KLK5) | -17 |
|  |  |  |  | nucleosome assembly protein 1-like 2 (NAP1L2) | -28 |
|  |  |  |  | Usher syndrome 3A (USH3A), transcript variant 1 | -39 |
|  |  |  |  | gamma-aminobutyric acid (GABA) A receptor, alpha 1 (GABRA1) | -99 |
|  |  |  |  | guanylate binding protein 1, interferon-inducible, 67kDa (GBP1) | -288 |
|  |  |  |  | interleukin 1, alpha (IL1A) | -421 |
